# Exploring the digital footprint of depression: a PRISMA systematic literature review of the empirical evidence

**DOI:** 10.1186/s12888-022-04013-y

**Published:** 2022-06-22

**Authors:** Daniel Zarate, Vasileios Stavropoulos, Michelle Ball, Gabriel de Sena Collier, Nicholas C. Jacobson

**Affiliations:** 1 Institute for Health and Sport, Victoria University, Melbourne, Australia; 2 Department of Psychology, University of Athens, Athens, Greece; 3 Center for Technology and Behavioral Health, Geisel School of Medicine, Dartmouth College, Hanover, USA; 4 Department of Biomedical Data Science, Geisel School of Medicine, Dartmouth College, Hanover, USA; 5 Department of Psychiatry, Geisel School of Medicine, Dartmouth College, Hanover, USA; 6 Quantitative Biomedical Sciences Program, Dartmouth College, Hanover, USA

**Keywords:** Digital phenotype, Ecological momentary assessment, Experience sampling, Passive sensing, Ambulatory assessment, Depression, PRISMA, Systematic literature review

## Abstract

**Background:**

This PRISMA systematic literature review examined the use of digital data collection methods (including ecological momentary assessment [EMA], experience sampling method [ESM], digital biomarkers, passive sensing, mobile sensing, ambulatory assessment, and time-series analysis), emphasizing on digital phenotyping (DP) to study depression. DP is defined as the use of digital data to profile health information objectively.

**Aims:**

Four distinct yet interrelated goals underpin this study: (a) to identify empirical research examining the use of DP to study depression; (b) to describe the different methods and technology employed; (c) to integrate the evidence regarding the efficacy of digital data in the examination, diagnosis, and monitoring of depression and (d) to clarify DP definitions and digital mental health records terminology.

**Results:**

Overall, 118 studies were assessed as eligible. Considering the terms employed, “EMA”, “ESM”, and “DP” were the most predominant. A variety of DP data sources were reported, including voice, language, keyboard typing kinematics, mobile phone calls and texts, geocoded activity, actigraphy sensor-related recordings (i.e., steps, sleep, circadian rhythm), and self-reported apps’ information. Reviewed studies employed subjectively and objectively recorded digital data in combination with interviews and psychometric scales.

**Conclusions:**

Findings suggest links between a person’s digital records and depression. Future research recommendations include (a) deriving consensus regarding the DP definition and (b) expanding the literature to consider a person’s broader contextual and developmental circumstances in relation to their digital data/records.

**Supplementary Information:**

The online version contains supplementary material available at 10.1186/s12888-022-04013-y.

## Introduction

The use of mobile devices, wearable technologies, and social media offers a wealth of health-related data to *objectively* assess symptoms of psychological disorders such as depression [96, 129]. In this context, research interest has emerged regarding the digital phenotyping (DP), or translation of a user’s tracing of digital data into health-related information [20, 84, 90, 95, 96, 99, 119]. However, considering the recent and rapid development of this field, conceptual clarification of specific terminology and a clear organization of depression-related evidence is needed [28]. Thus, the current research presents a systematic organization of available literature emphasizing (1) identification of empirical studies examining the use of digital data to study depression (with focus on the DP); (2) description of the different terms and digital data types employed; (3) integration of the evidence to ascertain the efficacy of digital data in the examination, diagnosis, and monitoring of depression; and (4) discussion of gaps within the field.

### The digital phenotype

A phenotype encompasses the behavioral expression(s) of a person’s predispositions under the effects of their life experiences [42]. Thus, it is assumed that a person’s behavioral phenotype/profile carries critical information about their physical and mental health conditions [42]. For example, disruptive eating or sleeping patterns could raise the possibility of depressed mood [133]. In this context, practices similar to the examination of the phenomenology of real-life behavior (i.e., behavioral phenotype) have recently migrated into the field of online behavior and digital records [5]. Specifically, past research has evaluated the extent that digital data and cyber-behavior involving a range of aspects (e.g., frequency and intensity of Internet use; applications of preference; digital records accessed via wearable devices, etc.) may encapsulate diagnostic information relevant to one’s overall health [54, 84, 107, 116, 119, 140, 145, 164, 175, 179, 195]. Accordingly, the concept of DP has been frequently applied to non-differentially describe the digital footprint of individuals’ physical and mental health conditions, as this can be inferred from their cyber-behavior and other digitally collected data (such as wearable technology and mobile device usage; [119]).

Past research also used terms such as *‘ecological momentary assessment’*, ‘*experience sampling methods’*, *‘passive sensing’*, *‘ambulatory assessment’*, *‘time-series analysis’*, *‘mobile sensing’*, *‘digital biomarkers’*, and *‘biosensing’* (among others) to encapsulate the quantification of individual behavior via digital means, and thus have been effectively employed as DP synonyms [28, 49, 74, 86, 91, 189]. While all these terms have been used to describe the capturing of individual data in a highly ecological (*‘in-situ’*) and highly granular manner, differences can be observed regarding the level of subjectivity/objectivity, the data collection means, and the nature/type of the measurements collected they reflect.

*Ecological momentary assessment* [EMA], and *experience sampling methods* [ESM] focus on the highly ecological nature of measurements, encompassing subjective (e.g., self-report surveys), independent of the nature of the data acquired (e.g., one’s emotions vs daily activities), and may/may not involve the use of digital means (e.g., paper–pencil questionnaires) [11, 49, 113]. Similarly, *ambulatory assessment* employs computer-assisted technology to capture subjective/objective data collection [80, 86, 168, 182]. Moreover, *passive sensing* appears more specific due to emphasizing data acquisition in an objective/passive manner; however, it does not differentiate the digital technology used and/or the type of data collected (e.g., mobile phone or other portable devices [38]). The type of digital data collected appears to become clearer via concepts such as *mobile sensing*, where the use of one’s mobile phone is assumed to acquire data passively/objectively (e.g., patterns/frequency and length of one’s calls/texts [38]). Furthermore, terms such as *digital biomarker* and *biosensing* describe the objective/passive collection of broader biological data (e.g., a measurement variable associated with a disease outcome) via the use of digital tools [57]. Finally, *time-series analysis* [TSA] refers to the methodological approach employed to analyse data time-patterns with high granularity [89]. Thus, considering the significant differences (Table 1) and current undifferentiated use of terms referring to active and passive data collection, a more precise taxonomy within mental health contexts is imperative.

**Table 1:** Identification of specific conditions related to each type of methodology in the available literature

	Necessary Criteria						
Methodology	Digital Technology	Biological measurements	Online Behaviour	Active data/ reporting	Passive data/ Objective sensor	Applications	Example
EMA/ ESM	No	No	No	Yes	No	Research strategy involving fine grained assessment of an individual’s immediate mental state within the context and flow of daily experience and one’s natural settings (Ben-Zeev et al.) [12]	Patient diaries
Digital Phenotype/ing	Yes	No	No	No	Yes	Overarching term, inclusive of *any* methodology involving the *objective* assessment/surveying of the digital footprint of individuals’ physical and mental health conditions in online and offline environments (Jain et al.) [96]. In this context, individual digital footprints arise as a residue of user/interface interaction	Social media use metrics
Passive sensing	Yes	No	No	No	Yes	Methodology involving digital technology capable of capturing daily activities and routines to assess multiple dimensions of human behavior (Narziev et al.) [138]	Geolocation information
Digital biomarkers	Yes	Yes	No	No	Yes	Digital biomarkers refer to quantifiable physiological information passively recorded via digital technology	Blood Pressure
Mobile sensing	Yes	No	No	No	Yes	Mobile sensing platforms enable the identification and tracking of human behavior from digital data passively collected from sensors embedded on mobile devices (Place et al.) [154]	Phone call frequency
Ambulatory assessment	Yes	No	No	No	No	This computer-assisted methodology allows researchers to obtain participant information multiple times daily while in their natural environments that may include passive and/or active data collection (Hepp et al.) [80]	Computer assisted self-reports

*EMA* Ecological Momentary Assessment, *ESM *Experience Sampling Method. Time-series analysis is defined as the analytic approach to examine rather than collecting data, and thus not included in this table

All six methodologies involve granularity as a necessary criterion. Granularity enhances the level of data detail, with smaller intervals of data collection resulting in greater detail and higher granularity (e. g. minutes compared with days)

### Digital phenotype potential

Despite the lack of conceptual clarity surrounding the DP definition, applying digital methodologies seems attractive and plausible [90, 119]. Specifically, increasing network connectivity embedded in electronic devices allows large quantities of data (i.e., big data) to be easily harvested [176]. When considering the significance of DP, ‘subjective/active data’ requires deliberate participant involvement in the collection process (e.g., questionnaire data), while ‘passive/objective’ digitalized data collection does not [177]. Thus, passive/objective sensing of data collected via digital technology has been proposed to increase the ecological validity of assessing psychopathological symptoms due to its (1) *‘*in situ*’* and (2) *‘moment-to-moment*’ characteristics [28].

Firstly, DP can be implemented ‘in situ*’* or within naturalistic environments removed from artificial settings such as psychiatric interviews with the potential to outweigh traditional clinical practice methods in reliability and validity [17, 22, 41, 55, 63, 90, 145, 161]. Indeed, scholars highlight advantages, including higher reliability of employing objective data (e.g., duration of one’s calls derived from the digital records of their smartphone) for assessing disordered behaviors compared to self-report surveys [55]. Secondly, the continuous or *‘moment-to-moment’* flow of information generates higher data granularity regarding the assessment of symptoms of psychopathology by more frequently capturing and quantifying the individual’s behavior and its variations over time [115, 179]. Research has documented the variability and instability of cognitive and affective depressive symptoms over time, suggesting the need for a method that permits the continuous assessment of symptomatology [40, 41].

DP frameworks have been implemented in a variety of mental health contexts such as bipolar disorder [26, 29] and social anxiety [19, 92]. However, the current study will focus exclusively on the use of DP to assess depressive symptoms.

### The digital footprint of depression

Depression is a pervasive disorder characterized by an overall negative affect that interferes with daily functioning [4]. Sufferers may exhibit several symptoms, including (but not limited to) reduced cognitive performance, insomnia, low mood/self-esteem, non-interactiveness/motor retardation, and reduced appetite [24, 185]. Previous research has employed digital technology to identify distinct associations between depressed participants and patterns of behavior [116, 118, 140, 169, 200]. For example, depression has been assessed via patterned psychomotor activity based on accelerometer oscillations and text/call behavior on mobile phones [93].

Depression was selected to be studied in relation to DP for its high lifetime prevalence in the general population (20.8% in the US; [101]), high comorbidity, differential diagnosis challenges [22], and its high variability within individuals [124]. For example, many individuals dealing with depressive episodes experience oscillations in suicidal ideation and thoughts of death, which may vary over their life course [4] and may be revealed by their online behavior and digital biomarkers [91]. Moreover, the nature of depressive symptoms has been reported to exert an adverse effect upon the active provision of accurate self-report and/or clinical interview-related information [19, 179]. Thus, passive/objective data collection based on DP procedures may provide a more accurate picture of the specific symptom profile of such individuals.

### The present review

Previous research has implemented the DP and related terminology to investigate depressive symptoms, and systematic reviews of the current literature have been conducted [7, 11, 34, 44]. However, existing reviews have focused on specific technologies (e.g., mobile phones exclusively [44]) or on specific theoretical frameworks (e.g., EMA exclusively [34]). Thus, comprehensive, and to an extent comparative, work focusing on the different digital technologies/methods for the study of depression is needed.

The present systematic literature review employs the PRISMA framework to address the following aims: (1) identify the common trends of empirical studies involving the use of digital data to study depression; (2) describe the different technologies and data employed; (3) integrate the evidence for this disorder; and (4) describe/clarify the use of different terms in relation to the data collected.

## Method

### Search strategy

The topic of this systematic literature review was registered on PROSPERO and received the registration number CRD42020186917 on 05/07/2020. Based on this, a computer (Boolean) search including [‘digital phenotype’ OR ‘digital phenotyping’ OR ‘passive sensing’ OR ‘digital biomarkers’ OR ‘ecological momentary assessment’ OR ‘experience sampling method’ OR ‘mobile sensing’ OR ‘ambulatory assessment’ OR ‘biosensing’ OR ‘smart sensing’ OR ‘activity recognition’ OR ‘crowdsensing’] AND [‘depression’ OR ‘depressive’ OR ‘depressed’] was conducted in Psycinfo, PubMed and Scopus databases on 01/06/2021.These databases were selected to cover a broad range of areas (such as artificial intelligence, linguistics and psychology). For example, Scopus and PubMed index the Institute of Electrical and Electronic Engineers (IEEE), Association for Computing Machinery (ACM), Multidisciplinary Digital Publishing Institute (MDPI), and bioinformatic journals (e.g., JAMIA among many others).

### Inclusion/Exclusion criteria

*Inclusion* criteria consisted of (a) empirical papers; (b) articles that included selected search terms in the title, abstract, and/or keywords; (c) papers that assessed depression severity (either at baseline to establish comparisons across depressed vs. non-depressed groups or as an outcome variable); and (d) articles that used digital technology in the assessment of one or more of variables. Considering the methodological heterogeneity of the included studies, no specifications were made in relation to time factor (i.e., longitudinal vs. cross-sectional; length of longitudinal assessment), study design (i.e., randomized control trial, etc.), and/or type of technology employed. *Exclusion* criteria consisted of: (a) articles that did not explicitly specify using digital means; (b) articles *only* using phone-call-based data collection (i.e., calling participants to complete surveys over the phone) due to limiting the ecological nature of data collection processes; and (c) papers evaluating psychometric properties of instruments measuring depression.

### Selection of studies

**Fig. 1:**
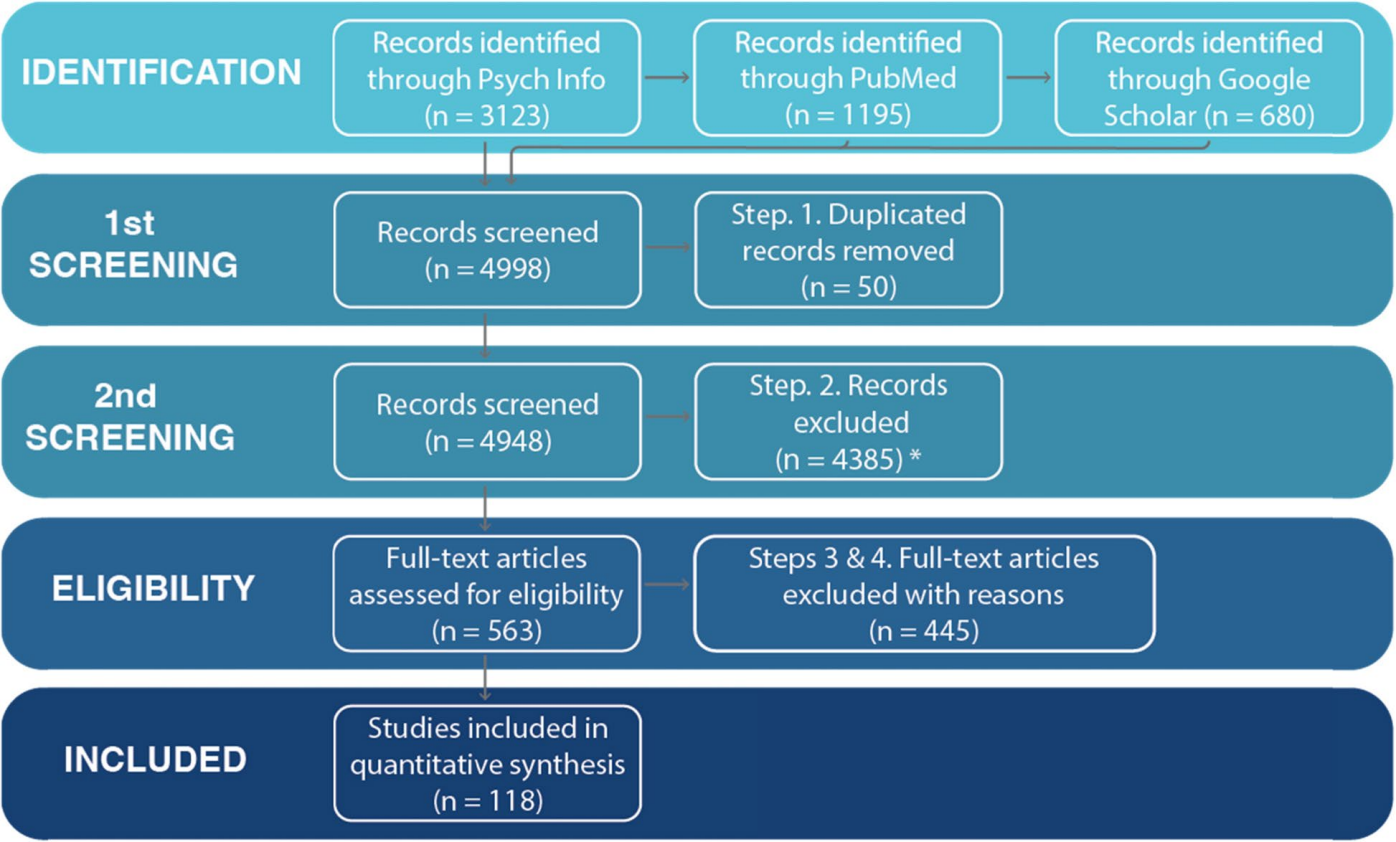
PRISMA flowchart of primary study selection. We excluded studies that exclusively called participants to conduct surveys over the phone given the limited ecological nature of such interventions. However, we have included studies that employed phone-based assessments where participants interact with pre-recorded messages. *Excluded if seacrh terms were not targeted in the article. **Excluded if study i) did not use digital technology to conduct momentary assessments, ii) conducted psychometric evaluations of questionnaires

Figure 1 depicts the PRISMA flowchart selection process to align with past relevant published reviews [3, 5, 180]. The search strategy produced 4998 relevant records overall (i.e., 3123 on Psycinfo, 1195 on PubMed, and 680 on Scopus). After removing duplicate records, an initial screening took place, excluding 4385 records that did not target selected search terms. Subsequently, two researchers (DZ and GdSC) systematically screened 563 relevant records excluding articles that did not address requirements (i.e., assessing depression and using digital technology). A third researcher (VS) resolved disagreements between researchers during the screening process. Next, a full-text assessment of these filtered articles revealed 445 records that failed to fulfill the outlined eligibility criteria. Studies excluded at this stage: (a) failed to address using digital technology to collect data; (b) conducted psychometric evaluations of questionnaires; (c) used *only* phone-call-based assessments; or (d) did not assess/presented results considering depression. Overall, 118 studies providing quantitative empirical evidence met the outlined criteria.

### Risk of bias assessment

To fulfill PRISMA framework guidelines, the Joanna Briggs Institute [98] checklist was adopted and modified to examine the risk of bias in cross-sectional and longitudinal analyses [123]. Assessed criteria included: (a) sample selection (i.e., randomization); (b) clearly stated participant eligibility criteria; (c) identification of potential confounding effects; (d) measurement bias; (e) adequate description of participant demographics; (f) follow-up time-length; (g) inclusion/explanation of participant attrition; (h) employment of appropriate and standardized measures to evaluate symptom severity; and (i) mitigation of bias in analysis by conducting multiple statistical analyses. A point was given for each criterion not addressed, with possible scores ranging from 0–9 in longitudinal studies (and 0–7 in cross-sectional studies), with higher scores representing higher risk of bias.

## Results

This section addresses the aims of the present study in the following order: (1) study characteristics (including design, demographic, and risk of bias); (2) technology used in the data collection, including the type of digital records employed (i.e., active/subjective and passive/objective); (3) depression-related empirical evidence acquired via the use of one’s digital traces; and (4) DP definitions and digital data mental health records terminology.

### Study design, demographic characteristics, and risk of bias

Most reviewed studies employed a longitudinal design (range of 1 day to 2 years), and only four studies employed a cross-sectional design [51, 162, 170, 198]. There was an even distribution between clinical (46%) and community (54%) samples, with university students (24%) as the most frequent population, followed by adults with a diagnosis of major depressive disorder (MDD; 21%) and adults from the general population (9%). Most studies utilized an adult sample (18–73 age range), with five studies (4%) using an adolescent sample (13–18 age range) and two studies (2%) using a sample of children (6–14 age range). Most studies utilized a larger proportion of female participants (81% of studies) and participants of white/Caucasian ethnic background (42% of studies). Additionally, all studies employed samples derived from developed countries (USA 57%, Netherlands 12%, Germany 6%, Belgium 6%, Canada 5%, etc.), and one study employed a combined sample including participants from developing countries (Brazil/USA). See Table 2 below (and for a more detailed account of studies including main findings see Supplementary Table 1).

**Table 2:** Summary of studies including sample type, definition employed to describe DP, type of digital technology used, and dimension of depression assessed

N	N / ref	First Author	Year	Clinical Sample	Definition	Digital technology / Type of data (active and/or passive)	Dimensions of depression
1	[1]	Abela	2007	No	ESM*	Handheld computer (A) #	Mood, cognitive style
2	[2]	Adams	2009	No	ESM*	Handheld computer (A) #	Mood, cognitive style
3	[6]	Bai	2021	Yes	DP*	Mobile app, Wrist sensor, GPS, Accelerometer, Smartphone comm logs, Screen activity (A/P)	Mood, psychomotor activity, sleep
4	[8]	Bartels	2020	No	ESM*	Handheld computer (A) #	Mood, social functioning, depression risk and protective factors (intervention)
5	[12]	Ben-Zeev	2009	Yes	ESM	Handheld computer (A) #	Mood
6	[13]	Ben-Zeev	2015	No	EMA*	Mobile app, GPS, Wi-Fi, Accelerometer, Audio recording, Ambient light (A/P)	Psychomotor activity, sleep, social functioning
7	[15]	Beute	2018	No	EMA	Mobile app (A) #	Mood, depression risk and protective factors (psychosomatic complains)
8	[16]	Bickham	2015	No	EMA*	Handheld computer (A) #	Depression risk and protective factors (media use)
9	[18]	Bos	2019	No	ESM*	Handheld computer (A) #	Mood
10	[21]	Bower	2010	Yes	ESM*	Handheld computer (A) #	Mood, sleep
11	[23]	Brose	2017	No	ESM*	Mobile app (A) #	Depression risk and protective factors (stress)
12	[24]	Brown	2011	No	ESM	Handheld computer (A) #	Mood, social functioning, cognitive performance
13	[25]	Burns	2011	No	EMA	Mobile app, GPS, Wi-Fi, Accelerometer, Smartphone comm logs, Ambient light (A/P)	Depression risk and protective factors (intervention)
14	[27]	Bylsma	2011	No	ESM	Handheld computer (A) #	Mood, social functioning
15	[29]	Cho	2019	Yes	DP	Mobile app, Wrist sensor, Accelerometer (A/P)	Mood, psychomotor activity, sleep
16	[30]	Chow	2017	No	ESM*, Mobile Sensing*	Mobile app, GPS (A/P)	Mood, psychomotor activity, social functioning
17	[31]	Chue	2017	No	EMA*	Handheld computer (A) #	Mood, social functioning
18	[33]	Clasen	2015	No	ESM*	Mobile app (A) #	Mood, cognitive style
19	[35]	Colombo	2020	No	EMA*	Mobile app (A) #	Mood
20	[37]	Cormack	2019	Yes	DP*	Mobile app, Wrist sensor, Accelerometer (A/P)	Mood, psychomotor activity, cognitive performance
21	[40]	Cushing	2018	No	EMA*	Mobile app, Accelerometer (A/P)	Mood, psychomotor activity
22	[45]	Dejonckheere	2019	No	ESM*	Mobile app (A) #	Mood, cognitive style
23	[46]	Demiralp	2012	Yes	ESM*	Handheld computer (A) #	Mood
24	[47]	Depp	2015	Yes	EMA	Mobile app (A) #	Depression risk and protective factors (intervention)
25	[49]	Di Matteo	2020	No	EMA	Mobile app, Smartphone comm logs (A/P)	Psychomotor activity, sleep, social functioning
26	[50]	Di Matteo	2021	No	Passive sensing*	Mobile app, Audio recordings (A/P)	Mood
27	[51]	Dietvorst	2021	No	ESM*	Mobile app, Online survey (A/P)	Mood
28	[52]	Difrancesco	2018	Yes	EMA*, AA*	Mobile app, Wrist sensor, Accelerometer (A/P)	Sleep, psychomotor activity
29	[53]	Eddington	2017	Yes	AA*	Mobile app (A) #	Depression risk and protective factors (intervention)
30	[56]	Elovainio	2020	No	EMA	Mobile app, Accelerometer, Online survey (A/P)	Sleep
31	[58]	Fang	2019	No	ESM	Mobile app (A) #	Cognitive style
32	[59]	Feiler	2005	Yes	Time series analysis	Handheld computer (A) #	Depression risk and protective factors (pain)
33	[65]	Gansner	2020	Yes	EMA	Mobile app (A/P)	Depression risk and protective factors (rheumatoid arthritis and pain)
34	[66]	Geyer	2018	No	EMA	Mobile app (A) #	Mood, social functioning
35	[67]	Giesbrecht	2012	No	EMA*	Handheld computer (A) #	Mood
36	[69]	Goldschmidt	2014	No	EMA	Handheld computer (A) #	Food intake
37	[71]	Graham-Engeland	2016	Yes	EMA	Handheld computer (A) #	Mood, depression risk and protective factors (rheumatoid arthritis and pain)
38	[72]	Gruber	2013	Yes	ESM	Handheld computer (A) #	Mood
39	[73]	Hahn	2021	No	DP*	Online survey (A) #	Mood
40	[74]	Hallensleben	2017	Yes	EMA	Mobile app (A) #	Suicidality
41	[76]	Hamilton	2020	No	EMA*	Mobile app (A) #	Sleep, depression risk and protective factors (social media use)
42	[77]	Hartmann	2015	Yes	ESM	Handheld computer (A) #	Mood, depression risk and protective factors (intervention)
43	[79]	Heninga	2019	Yes	ESM*	Mobile app (A) #	Mood, social functioning, psychomotor activity
44	[80]	Hepp	2019	Yes	AA	Handheld computer (A) #	Mood, social functioning
45	[82]	Hershenberg	2017	Yes	ESM*	Interactive voice recording (A) #	Mood, cognitive style
46	[83]	Holmes	2016	Yes	Time series analysis	Mobile app, Online survey (A) #	Depression risk and protective factors (intervention)
47	[85]	Huckins	2020	No	EMA	Mobile app, GPS, Accelerometer, Screen activity, Ambient light (A/P)	Depression risk and protective factors (COVID-19)
48	[86]	Huffziger (a)	2013	Yes	AA*	Handheld computer (A) #	Mood, cognitive style
49	[87]	Huffziger (b)	2013	No	AA*	Handheld computer (A) #	Mood, cognitive style
50	[88]	Hung	2016	Yes	EMA*	Mobile app (A) #	Mood, sleep, cognitive performance
51	[89]	Husky	2009	No	ESM*	Handheld computer (A) #	Mood
52	[91]	Jacobson (a)	2019	Yes	Digital biomarkers*	Wrist sensor, Accelerometer (P)	Psychomotor activity
53	[92]	Jacobson (b)	2019	Yes	DP*	Wrist sensor, Accelerometer, Ambient light (P)	Psychomotor activity
54	[93]	Jacobson (a)	2020	No	DP*	Mobile app, Accelerometer, Smartphone comm logs (A/P)	Social functioning, psychomotor activity
55	[94]	Jacobson (b)	2020	No	DP*, Passive sensing*	Mobile app, GPS, Wi-Fi, Smartphone comm logs, Ambient light (A/P)	Mood, social functioning, psychomotor activity
56	[97]	Jean	2013	Yes	EMA*	Handheld computer (A) #	Depression risk and protective factors (dep risk following stroke)
57	[100]	Kaufmann	2016	Yes	EMA	Mobile app (A) #	Mood, sleep
58	[102]	Khazanov	2019	Yes	EMA*	Handheld computer (A) #	Mood, cognitive style
59	[103]	Kim	2013	No	EMA	Handheld computer, Wrist sensor, Accelerometer (A/P)	Mood, psychomotor activity
60	[104]	Kim	2014	Yes	EMA*	Handheld computer, Wrist sensor, Accelerometer (A/P)	Mood, psychomotor activity
61	[105]	Kim	2019	No	EMA	Handheld computer, Wrist sensor, Accelerometer (A/P)	Mood, psychomotor activity, sleep
62	[106]	Kircanski	2015	No	ESM*	Handheld computer (A) #	Cognitive style
63	[109]	Koval	2013	No	ESM*	Handheld computer (A) #	Mood
64	[110]	Kramer	2014	Yes	EMA*, ESM	Handheld computer (A) #	Depression risk and protective factors (intervention)
65	[114]	Lavender	2013	Yes	EMA*	Handheld computer (A) #	Mood, food intake
66	[121]	Maher	2018	No	EMA	Mobile app (A) #	Mood
67	[122]	Mak	2020	Yes	EMA*	Handheld computer (A) #	Mood, dep risk and protective factors (rheumatoid disease and pain)
68	[125]	Mata	2012	Yes	ESM*	Handheld computer (A) #	Mood, psychomotor activity
69	[126]	McIntyre	2021	Yes	EMA	Mobile app, GPS, Smartphone comm logs (A/P)	Psychomotor activity, social functioning
70	[128]	Melcher	2021	No	DP	Mobile app, GPS, Accelerometer (A/P)	Psychomotor activity, sleep
71	[130]	Minaeva (a)	2020	Yes	ESM, AA*	Mobile app, Accelerometer (A/P)	Mood, psychomotor activity, sleep
72	[131]	Minaeva (b)	2020	No	EMA*, AA*	Wrist sensor, Accelerometer (P)	Mood, psychomotor activity
73	[134]	Moreno	2012	No	ESM	Mobile app, Online survey (A) #	Depression risk and protective factors (Internet use)
74	[135]	Moshe	2021	No	DP	Mobile app, Ring, GPS, Accelerometer, Smartphone comm logs, Screen activity (A/P)	Mood, psychomotor activity, sleep
75	[136]	Moukaddam	2019	Yes	DP*, EMA	Mobile app, GPS, Accelerometer, Smartphone comm logs, Screen activity, Ambient light (A/P)	Mood, social functioning, sleep
76	[138]	Narziev	2020	No	EMA*, Passive sensing	Mobile app, Wrist sensor, Accelerometer, Smartphone comm logs, Screen activity, Ambient light (A/P)	Mood, psychomotor activity, social functioning, sleep
77	[139]	Nelson	2018	Yes	EMA*	Mobile app (A) #	Mood
78	[141]	Nook	2021	No	ESM	Mobile app (A) #	Mood
79	[142]	Nylocks	2019	Yes	ESM*	Handheld computer (A) #	Mood, psychomotor activity
80	[143]	Odgers	2017	Yes	EMA*	Mobile app (A) #	Depression risk and protective factors (exposure to violence)
81	[144]	O’Leary	2017	Yes	ESM	Handheld computer (A) #	Mood, sleep
82	[147]	Panaite	2018	Yes	EMA*	Mobile app, Voice recording (A) #	Mood
83	[148]	Panaite	2019	No	EMA*	Handheld computer (A) #	Mood, cognitive style
84	[150]	Pasyugina	2015	No	ESM	Mobile app, Voice recording (A) #	Mood, cognitive style
85	[151]	Pe	2014	No	ESM*	Handheld computer (A) #	Mood
86	[152]	Pedrelli	2020	Yes	DP	Mobile app, Wrist sensor, GPS, Accelerometer, Smartphone comm logs (A/P)	Psychomotor activity, social functioning
87	[153]	Peterson	2020	Yes	EMA*	Mobile app (A) #	Depression risk and protective factors (intervention)
88	[154]	Place	2017	Yes	Mobile sensing	Mobile app, GPS, Wi-Fi, Accelerometer, Smartphone comm logs, Screen activity, Speech technology (A/P)	Psychomotor activity, social functioning
89	[155]	Putnam	2007	Yes	EMA*	Handheld computer (A) #	Cognitive style
90	[157]	Robbins	2011	Yes	EMA*, AA*	Audio recordings (P)	Depression risk and protective factors (rheumatoid arthritis)
91	[158]	Rodriguez	2021	No	ESM	Mobile app (A) #	Depression risk and protective factors (social media use)
92	[183]	Roekel	2016	No	ESM	Mobile app, handheld computer (A) #	Mood
93	[160]	Sagar	2016	Yes	EMA	Handheld computer (A) #	Depression risk and protective factors (marihuana use)
94	[162]	Schultebraucks	2020	Yes	DP*	Speech technology and facial recognition (A/P)	Psychomotor activity
95	[163]	Sears	2018	No	ESM*	Mobile app (A) #	Mood, social functioning
96	[166]	Sheets	2020	Yes	EMA	Handheld computer (A) #	Mood
97	[167]	Snippe	2016	Yes	ESM*	Handheld computer (A) #	Depression risk and protective factors (intervention)
98	[168]	Sperry	2018	No	ESM*, AA	Mobile app, Handheld computer, Chest patch (A/P)	Psychomotor activity, social functioning, food intake
99	[170]	Stasak	2019	Yes	DP*	Speech technology and facial recognition (A) #	Psychomotor activity, cognitive performance
100	[171]	Steenkamp	2019	Yes	ESM	Handheld computer (A) #	Depression risk and protective factors (childhood abuse)
101	[172]	Thompson	2015	No	ESM*	Handheld computer (A) #	Mood
102	[173]	Thompson	2016	Yes	ESM*	Handheld computer (A) #	Mood, cognitive style
103	[174]	Thompson	2017	Yes	ESM*	Handheld computer (A) #	Mood
104	[181]	Trull	2008	Yes	EMA	Handheld computer (A) #	Mood
105	[184]	Vansteelandt	2019	Yes	EMA	Handheld computer (A) #	Cognitive style
106	[186]	Verkuil	2015	No	EMA*	Handheld computer, Chest patch (A/P)	Mood, psychomotor activity
107	[187]	Vesel	2020	No	Digital biomarker*	Mobile app, Typing metadata (A/P)	Psychomotor activity, cognitive performance
108	[188]	Vranceanu	2009	No	EMA	Handheld computer (A) #	Mood, social functioning
109	[189]	Wahle	2016	No	Mobile sensing	Mobile app, GPS, Wi-Fi, Accelerometer, Smartphone comm logs (A/P)	Depression risk and protective factors (intervention)
110	[190]	Wang	2021	No	ESM*	Mobile app (A) #	Mood
111	[191]	Wenze	2006	No	ESM*	Handheld computer (A) #	Mood, cognitive style
112	[192]	Wenze	2009	No	ESM*	Handheld computer (A) #	Mood, cognitive style
113	[193]	Wenze	2012	No	ESM*	Handheld computer (A) #	Mood
114	[194]	Wenze	2018	No	EMA	Mobile app (A) #	Mood, social functioning
115	[196]	Worten-Chaudhari	2017	No	EMA*	Mobile app (A) #	Depression risk and protective factors (intervention)
116	[197]	Wu	2016	No	ESM*	Handheld computer (A) #	Cognitive style
117	[198]	Zhang,	2019	No	Digital biomarker*	Speech technology and facial recognition (A) #	Psychomotor activity
118	[200]	Zulueta	2018	Yes	DP	Mobile app, Accelerometer, Typing metadata (A/P)	Psychomotor activity, cognitive performance

* = Studies where methodology is mentioned without definition; # = Studies that exclusively use self-reported/clinical interview of symptoms of MDD to predict or assess the association with self-reported/clinical interview outcomes. *EMA* Ecological Momentary Assessment, *ESM* Experience Sampling Method, *DP* Digital Phenotype/ing, *AA* Ambulatory Assessment, *N/Ref*  Study as numbered in the reference list, *Smartphone comm logs* Call/SMS frequency, timing, and duration, *A *Active/subjective data collection, *P* Passive/objective data collection, *handheld computer* this term refers exclusively to portable electronic devices such as a Palm that can be used to collect self-reported information (it should be noted that mobile phones are not considered handheld computers)

Studies reviewed here represent a moderate risk of bias and acceptable quality. Observed scores ranged from 1 to 5 (higher scores represent a higher risk of bias) with a mean of 2.63 (*SD* = 0.90), 97.5% of studies < 5, and 84.7% < 4. The largest source of points given was due to not including follow-ups greater than one year (only four studies included follow-ups > one year) and the employment of a researcher-selected participant sample, with only 29 studies incorporating sample randomization (see Supplementary Table 2 for a detailed risk of bias assessment including an explanation of scoring system).

### Digital data sources and participant involvement

**Fig. 2:**
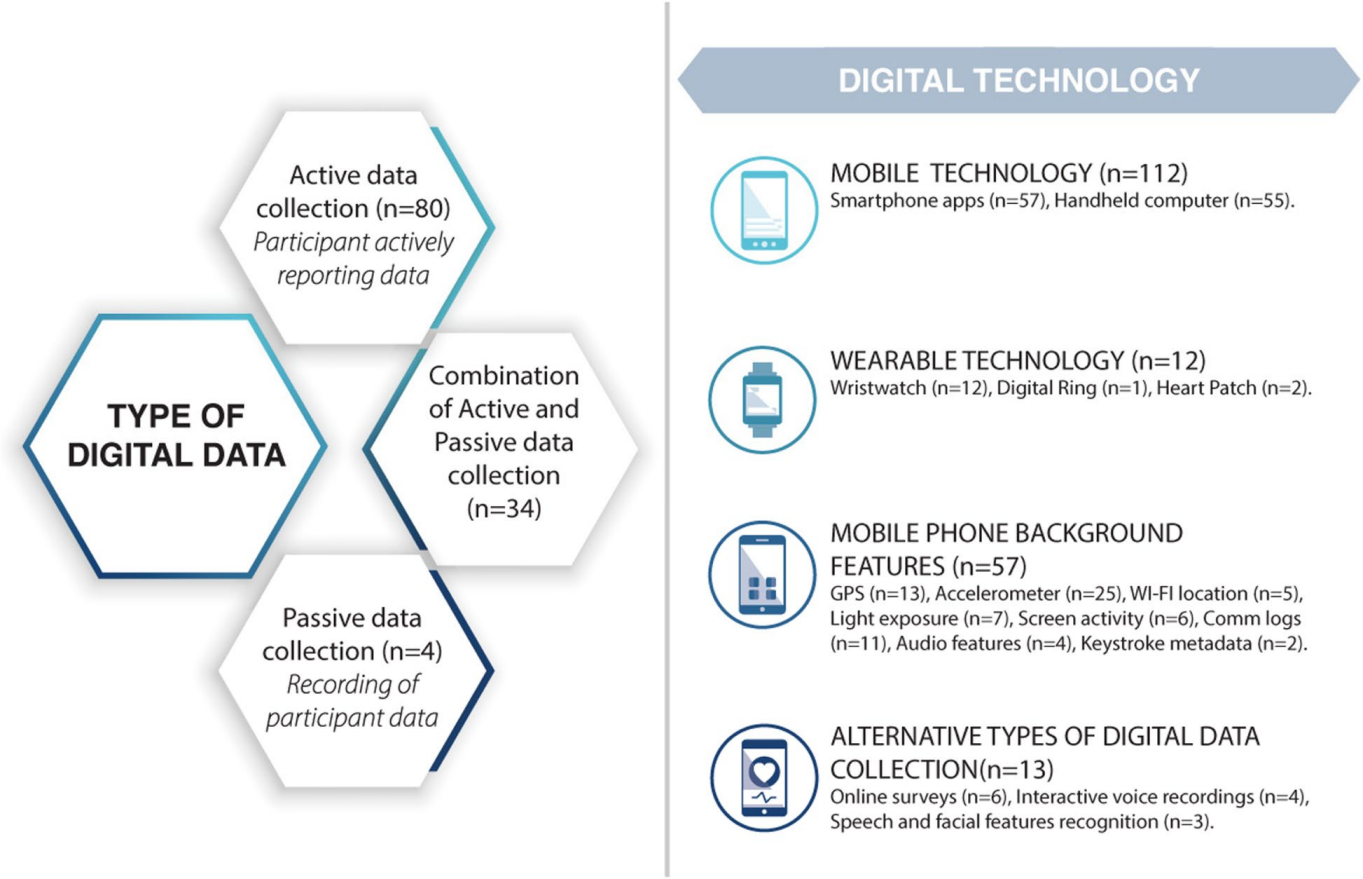
This figure illustrates the type of digital data and technology used by studies included in this review.The left panel shows how many studies active data collection, passive data collection,and a combination of both. The right panel illustrates how many studies used each type of technology. For example 13 studies employed mobile phone embedded GPS, and 11 studies used mobile phone communication logs (such as SMS, call frequency, call duration and email usage)

Studies used a range of technologies to collect active/subjective, passive/objective, and mixed (i.e., active/subjective and passive/objective) data. Studies employing passively/objectively collected data often produced predictive models with high accuracy in the detection of depression severity involving significant predictors such as geospatial movement, sleep duration, delayed sleep phase, circadian rhythm, audio features, language, accelerometer oscillation, and light exposure during bedtime [12, 29, 49, 50, 52, 56]. Considering the type of technology, reviewed studies employed mobile technology (handheld IT devices such as smartphones, palmtops, tablets, laptops, etc.; [81]), wearable, mobile phone background features, and alternative technology (see Fig. 2).

Specifically, mobile technology involved smartphone applications and/or palmtops requesting participants to complete self-reported momentary assessments providing a range of information (e.g., mood, cognitive capacity, suicidal thoughts, sleep–wake cycle, stress, physical activity, depression severity, social interaction, etc.). Mobile technology-associated features involved surveying participants’ movement/activity levels via background tracking features embedded in smartphones (GPS, accelerometer/actigraphy, Wi-Fi location, screen activity, light exposure, keystroke metadata etc.). For example, Zulueta et al. [200] observed variations in daily typing speed and frequency of backspaces (denoting errors) to assess depression. Additionally, much like background tracking features embedded in smartphones, wearable technology includes technology that can be worn (e.g., heart patch) and enables moment-to-moment passive/objective data collection, allowing for increased data granularity resulting in increased diagnosis effectivity. For example, Cho et al. [29] observed decreased regularity of sleep–wake cycle (as measured by heart rate) in participants with depressed mood, resulting in 71% accuracy in the prediction of depressive episodes (the model included other predictive variables such as light exposure).

Finally, other reviewed studies used alternative means of digital data collection, including online surveys, interactive voice recordings, and speech and facial features recognition. Much like momentary assessments completed via smartphones or handheld devices, online surveys were triggered by SMS text messages at semi-random intervals requiring participants to complete questions about their sleep habits, mood, stress, etc. Similarly, studies employing interactive voice recordings collected momentary assessments during phone calls in which participants responded to pre-recorded automated surveys. Considering speech and facial features recognition, studies evaluated: acoustic/prosodic speech features (i.e., pitch, intonation, loudness, and pause length), speech content (i.e., linguistic dimensions identified via linguistic inquiry word count, LIWC), and facial features (including facial expressivity, movement, and pupil dilation). These features were extracted from audio and video recordings to predict depression severity with acceptable accuracy. For example, Schultebraucks et al. [162] observed a predictive accuracy of AUC = 0.86, with linguistic features, voice prosody, facial features of emotion, and movement features (such as pupil dilation) as the most important predictors.

### Depressive behaviour digital traces

Regarding digital records related to one’s depressive behaviors, reviewed studies targeted different aspects of depression focusing on affective, somatic, and cognitive changes in participants. Studies employed standardized measures^1^ to establish baseline depression severity and subsequently evaluate the degree of relationship. The most investigated aspect of depression was mood^2^ (72 studies), followed by psychomotor activity^3^ (33 studies), social functioning4 (21 studies), cognitive style^4^ (19 studies), sleep quality^5^ (16 studies), cognitive performance^6^ (6 studies), food intake [69, 114, 168], and suicidality [74]. In addition, 27 studies^7^ evaluated the associations between depressive symptoms and variables of interest (for example social media use) or interventions to reduce depression severity (for example providing regular feedback vs no feedback).

Overall, most studies (including both clinical and non-clinical samples) reported associations between increased depression severity and higher negative affect, lower levels of physical activity, decreased social functioning, increased variability in sleep quality, decreased cognitive performance, and depressive cognitive styles (e.g., trait rumination, reassurance-seeking, etc.). Table 3 summarizes findings acquired via the range of technologies applied (for a more detailed account of findings, see Supplementary Table 1).

**Table 3:** Summary of depression-related empirical evidence

**Dimension of Depression**	Technology used	Consensus	Disagreement
**Mood** **(*n* = 72)**	Participants were prompted to complete questionnaires via alarms or ‘beeps’ at semi-random intervals over a range of consecutive days using handheld devices (e.g., palmtops), smartphones and online surveys	Higher negative affect (NA), lower positive affect (PA), lower interest/pleasure in activities, less emotion regulation strategies, and higher variability in affect correlated with depression severity. Additionally, depressed participants tended to overestimate prospective NA indicating a predisposition to have a pessimistic life perspective. Finally, depressed participants reported a larger decrease in dysphoria, sadness, and anxiety when exposed to a positive event compared to healthy participants	While most reviewed studies reported that depressed participants showed higher fluctuations of NA, 2 studies using clinical samples (Gruber et al. [72]; Heininga et al.) [79], and 1 study using non-clinical sample (Pe et al.) [151] observed no significant differences across depressed and non-depressed participants in PA mean values and variability
**Psychomotor Activity** **(*n* = 33)**	Active data collection via questionnaires via mobile apps, handheld devices, and online questionnaires. Passive data collection via wearable technology, GPS, accelerometer/actigraph, Wi-Fi location, smartphone usage, and typing metadata	Lower levels of physical activity were associated with increased levels of negative affect, depressive feelings, and anhedonia (e.g., reduced ability to enjoy pleasurable activities)	Two studies employing non-clinical samples and GPS-derived data found no significant associations between these variables (Chow et al. [30]; Melcher et al.) [128]
**Social Functioning** **(*n* = 21)**	Active data collection via self-reported questionnaires, and passive data collection via smartphone embedded audio features, and phone call/SMS frequency	Increased levels of depression severity associated with preference for being alone, increased social distance, reduced closeness with other individuals, increased interpersonal stress, reduced speech duration, and reduced phone call and SMS frequency Depression severity showed an association with reliance on social expression such that higher reliance on social expression of feelings (i.e., anger) predicted a decrease in depression severity over time (Chue et al.) [31]	Moukaddam et al. [136] used a clinical sample and found no correlations between depression levels and social interaction (SMS and phone call length and frequency)
**Sleep Quality** **(*n* = 16)**	Assessment of sleep quality involved self-reported questionnaires, accelerometer inferences (e.g., total steps during bedtime), GPS-derived data, actigraphy, smartphone embedded light sensors (e.g., increased light exposure during bedtime), smartphone use (screen on/off), sound features (e.g., ambient silence), and heart rate (assessed via wearable technology)	Most studies detected associations in variability of sleep quality and depression severity. Specifically, studies observed depression scores to be positively correlated with delayed sleep phase, sleep disturbance during weeknights, poor sleep quality, sleep variability, insomnia, and increased exposure to light during bedtime (Ben-Zeev et al. [12]; Di Matteo et al., [49]; Difrancesco et al., [52]; Elovainio et al., [56]; Hung et al., [88]; Kaufmann et al., [100]; Kim et al., [105]; Melcher et al.,) [128]	Two studies (1 clinical and 1 non-clinical sample) did not find significant correlations between self-reports of sleep duration and depression (Difrancesco et al., [52]; Hamilton et al.) [76]. Additionally, 2 studies using non-clinical samples found no significant associations in depression levels and sleep quality assessed via actigraph (Melcher et al.) [128] and self-reports (Hamilton et al.) [76]
**Cognitive Style** **(*n* = 19)**	Assessment of relationships between depression severity and cognitive style (including trait rumination, self-criticism, reassurance seeking, etc.) involved self-reported questionnaires collected via smartphones and digital devices	Studies observed positive associations between depression severity and fluctuations in self-assessment, reassurance seeking, emotional dependency, self-criticism, trait rumination, experiential avoidance, expressive suppression, and ‘should’ PA (i.e., the pressing feeling that they should experience positive affect)	
**Cognitive Performance** **(*n* = 6)**	Assessment of cognitive performance involved questionnaires (i.e., accordance to statements such as “I have trouble concentrating right now” (Brown et al.) [24], time spent and frequency of errors completing questionnaires (Hung et al.) [88], typing kinematic performance (Vesel et al. [187]; Zulueta et al.) [200], and cognitive tasks (Cormack et al. [37]; Stasak et al.) [170]	Studies observed that higher depression severity resulted in higher thought impairment, fewer clear thoughts, more concentration problems, and reduced cognitive performance	Hung et al. [88] observed that depressed participants did not take longer or make more mistakes than controls in completing questionnaires about mood and quality of sleep

*n* represents the number of studies assessing distinct dimensions of depression. Interestingly, no studies included in this review employed natural language processing of passively collected data via social media posts to capture mood or cognitive style

### Definitions and terminology used to describe the digital phenotype

**Fig. 3:**
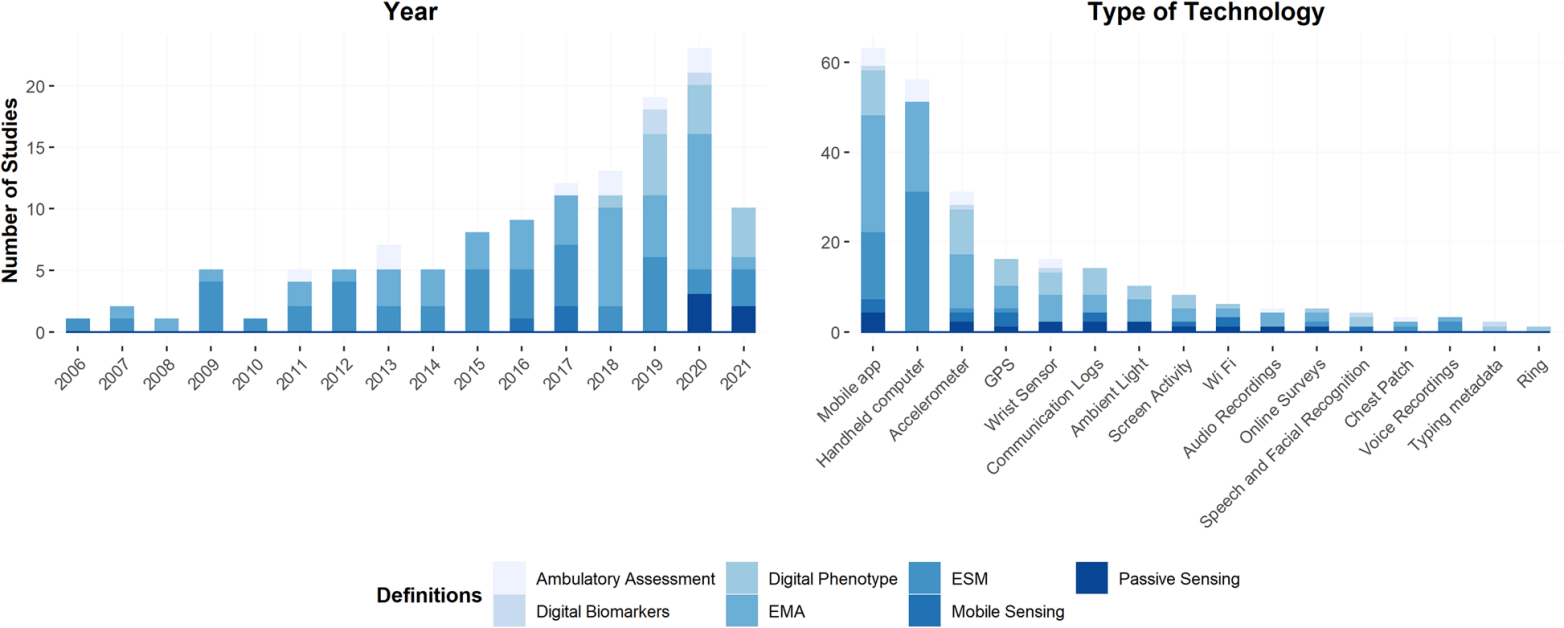
Number of studies using each definition discriminated by year (in the left panel) and by type of technology (in the right panel). Studies using the word 'digital' in their employed methodology (i.e., digital biomarkers) were published since 2018. Additionally, the majority of studies using ESM and EMA as selected methodology employed digital technology that relies on active data (i.e.,mobile apps and handheld computers)

There was a general agreement among reviewed studies that methodologies involving fine-grained observations in naturalistic settings can increase effectiveness and accuracy in evaluating psychopathology. However, alternative terms such as ecological momentary assessment (EMA, *n* = 48), experience sampling method (ESM, *n* = 44), digital phenotype/ing (DP, *n* = 14)*,* ambulatory assessment (AA, *n* = 8), passive sensing (*n* = 5), mobile sensing (*n* = 3), digital biomarkers (*n* = 3), and time-series analysis (*n* = 2) have been utilized in the studies reviewed. These terms were used in an undifferentiated manner to reflect methodologies involving high granularity and naturalistic observations (no studies employed the term biosensing, smart sensing, activity recognition, or crowdsensing). In addition, 59% of studies (*n* = 70) omit including a formal definition of the methodology used, while simply highlighting the benefits involved in the use of momentary assessments. Figure 3 shows the frequency distribution of definitions employed by studies discriminated by year and type of technology. Interestingly, terms referring exclusively to digital technology (i.e., *DP* and *Digital biomarkers*) have increased in the last five years (2016 – 2021). Additionally, most reviewed studies using technology needing active/subjective data collection (i.e., mobile apps and handheld computers) align with ESM and EMA definitions.

Reviewed studies define DP as the ability to measure phenotypical/ behavioral expressions through any digital means without consistently assuming the collection of passive/objective data [29, 128, 135, 152, 200]. This perspective includes *any* methods of quantifying an individual’s online and offline behavior via personal digital devices, *in*-*situ*, and in real-time [178]. Reviewed studies also utilized the terms *EMA* and *ESM* to conceptualize fine-grained assessments of depression severity when digital technology was used. However, considering that *EMA* and *ESM* were initially coined in the pre-digital era, confusion with studies non explicitly using digital technology occurred [112, 117].

Studies employing terms such as *mobile sensing* and *passive sensing* highlighted similar features elaborating on the ability to *passively* detect behaviors that might be related to specific mental disorders. Nonetheless, *mobile sensing* assumes the exclusive use of smartphone-derived data [154, 189], while *passive sensing* tends to involve the use of other mobile/portable and alternative types of technology (such as chest patch or digital rings) to conduct momentary assessments [168]. Interestingly, *ambulatory assessment* was used undifferentiated to capture passive and active data collection (i.e., self-reports) via computer-assisted technology [52, 80]. Reviewed studies employing the term *biomarker*, or *digital biomarker* did not provide clear definitions or explanations regarding what distinguishes this methodological approach. Nonetheless, a biomarker is a measurement variable associated with a disease outcome, usually derived from internal body function rates (e. g. blood pressure; cholesterol levels). Digital biomarkers are considered digital due to utilizing sensors and computational tools (e.g., wearable technology, smartphone sensors) to conduct passive/objective data collection [149]. Finally, *time-series analysis* has been identified as the analytic approach to evaluating data time patterns with high granularity [89].

Interestingly, most studies did not elaborate on the utilization of active/passive data collection methods, and only a handful of studies saw the need to provide clarification. Narziev et al. [138] make an explicit distinction between *EMA* and *passive sensing,* indicating that *EMA* refers to active/subjective participant involvement (i.e., tasks or questionnaires) and *passive sensing* refers to passive/objective participant involvement (i.e., actigraph, GPS or heart rate monitoring). Similarly, Di Matteo et al. [49] explicitly differentiate *EMA* from *passive EMA,* implying the need to differentiate these two concepts. This suggests that the employment of digitalized active data collection (such as digital EMA and ESM) currently encompasses a broad designation of methods, that may lack descriptive value and utility, while likely adding a convoluted interpretation/implementation to the field.

## Discussion

The use of digital records for assessing and treating depressive behaviors, has been receiving increased attention [17, 20, 90, 146]. Specifically, researchers highlighted the potentially increased ecological validity of *moment-to-moment* and *in-situ* assessments of disorder-related symptoms using digital technology [28, 177]. Considering the relatively recent emergence of this field, the current work aimed to employ a PRISMA framework to provide a comprehensive review of available literature using digital means/records to study depression with focus on the DP. In addition, it aimed to summarize the methods and types of data used in the assessment/monitoring of depressive symptoms and to identify areas of potential future research priority. Finally, it focused on identifying and differentiating the terms mostly utilized to describe how a person’s digital records could contribute to understanding their depressive mood. Findings illustrate that: (a) there is a promising potential in the use of one’s digital traces (i.e., digitally monitored/recorded data); (b) current research trends appear skewed towards specific age groups and national populations, likely restricting generalizability; and (c) inconsistencies occur regarding the meanings attached to the terminologies used (e.g., DP).

### Overview and research trends

In total, 118 eligible studies of a moderate/good quality were reviewed, revealing that research concerning the digital footprint of depression tends to be recent and skewed towards adult samples and developed countries. Additionally, there was a significant focus on assessing mood/affect compared to other clusters of symptoms related to depression (e.g., psychomotor activity, sleep quality, social functioning, etc.; Table 3). Finally, the field appears to be expanding rapidly, given that most studies included in this review were published within the most recent four years, with a progressively increasing trend per calendar year over time (no time restriction was applied in our inclusion criteria; Fig. 3). Considering these observations, future research may wish to address the current gaps in the field by assessing symptoms of depression associated with different aspects/dimensions of the disorder (e.g., psychomotor activity), using non-adult samples, and (more importantly) investigating populations from non-developed countries.

### Digital data sources and participant involvement

The reviewed studies used a wide variety of methods employing digital means to obtain phenotypical expressions of individuals’ depressive symptoms, including self-report apps, smartphone keystroke metadata, mobile phone calls and texts, online surveys, actigraphy sensor-related recordings (i.e., steps, sleep, circadian rhythm, GPS), and even digital records of voice. A common rationale to collect data via digital technology involved reflecting on the ubiquitous presence of mobile phones facilitating self-report momentary assessments and highlighting the potential to obtain otherwise elusive information on one’s social engagement behavior [19, 177]. Indeed, mobile phones in combination with wearable technology represented most instances of digital data collection methods reviewed here. This highlights the potential benefits of mobile phones and/or wearable technology sourced data within psychiatry and psychology [60, 63, 145, 164, 169, 177, 179].

Previous studies have also illustrated the potential value of social media content for understanding mental health [36, 43, 68, 112, 137]. Nonetheless, such information was not explored in depth in the studies reviewed here, indicating the need for further research. Similarly, while smartphone keystroke metadata and wearable technology (e.g., heart patch, wristwatch, etc.) show promising results, the evidence indicates that it has been employed scarcely. At this point, it should be noted that this PRISMA literature review did not include specific search terms such as ‘actigraph’ or ‘GPS’, and thus studies employing such methodology without mentioning the use of DP may not have been included.

### Depressive behaviours digital traces

Considering the empirical evidence of assessment of depression via digital technology, dimensions of depressive symptoms (i.e., mood, psychomotor activity, social functioning, sleep, and cognitive performance) were captured using a variety of digital technologies. These included smartphone-facilitated momentary assessments, mood logs, actigraphy data such as daily steps, GPS-derived activity, sleep, heart rate, light sensors recordings, SMS length and count, phone call data, keystroke meta-data, one’s geocoded activity, and speech technology.

Interestingly, studies made emphasis on positive associations between the *variability* of one’s sleep, affect and other psycho-motor aspects of depression and self-reported symptom severity [13, 49, 52, 56]. Thus, variability in depressive signs, such as depressive affect, lack of concentration, lack of motivation/pleasure for social engagement, or suicidal ideation/intention, appear to be accurately captured via digital technology. This highlights the advantageous nature of the DP when assessing within-individuals variability of depressive symptoms (e.g., over time patterns of depressive behaviors within the same person). Additionally, evidence was provided to support the viability of the DP in predicting depression and monitoring the effectiveness of targeted interventions [29, 90, 136, 187, 200]. For example, studies employed GPS-derived data to infer participant movement and thus predict depression severity with high accuracy [126]. These suggest that the DP can effectively be used to identify, monitor, and predict both between-individuals (i.e., how different individuals may experience the same symptoms), but more importantly within-individual (i.e., how different depressive manifestations may present with varying trajectories regarding the same individual) variations of depressive symptoms.

Although these observations support the rationale for employing DP in identifying depression symptom severity, lack of support was also observed. Contrary to hypothesized relationships, some reviewed studies observed no relationships between depression severity and variability in positive affect [72, 79, 151], reduced sleep quality [52, 76, 128], reduced psychomotor activity [30, 128], reduced social functioning [136], and cognitive performance [88]. This supports that while promising results are evident, further calibration/assessment in employed digital technology is required.

### Conceptual challenges

Most studies reviewed here utilized the terms ‘ecological momentary assessment [EMA]’, ‘experience sampling method [ESM]’ and ‘digital phenotype/ing [DP]’ to describe any form of in situ*,* passive, digital data/record collection. Similarly, a minority of the reviewed studies used ‘ambulatory assessment’, ‘digital biomarkers’, ‘passive sensing’, ‘mobile sensing’, and ‘time-series analysis’. However, studies reviewed employed such terms interchangeably, highlighting the occurrence of conceptual heterogeneity in their application regarding three dimensions: (a) the use of digital/non-digital technology; (b) the use of active and/or passive data; and (c) the domain/means/types of collected data employed (e.g., online/offline behaviors; mobile phone usage/ other wearable/portable devices usage data).

**Fig. 4:**
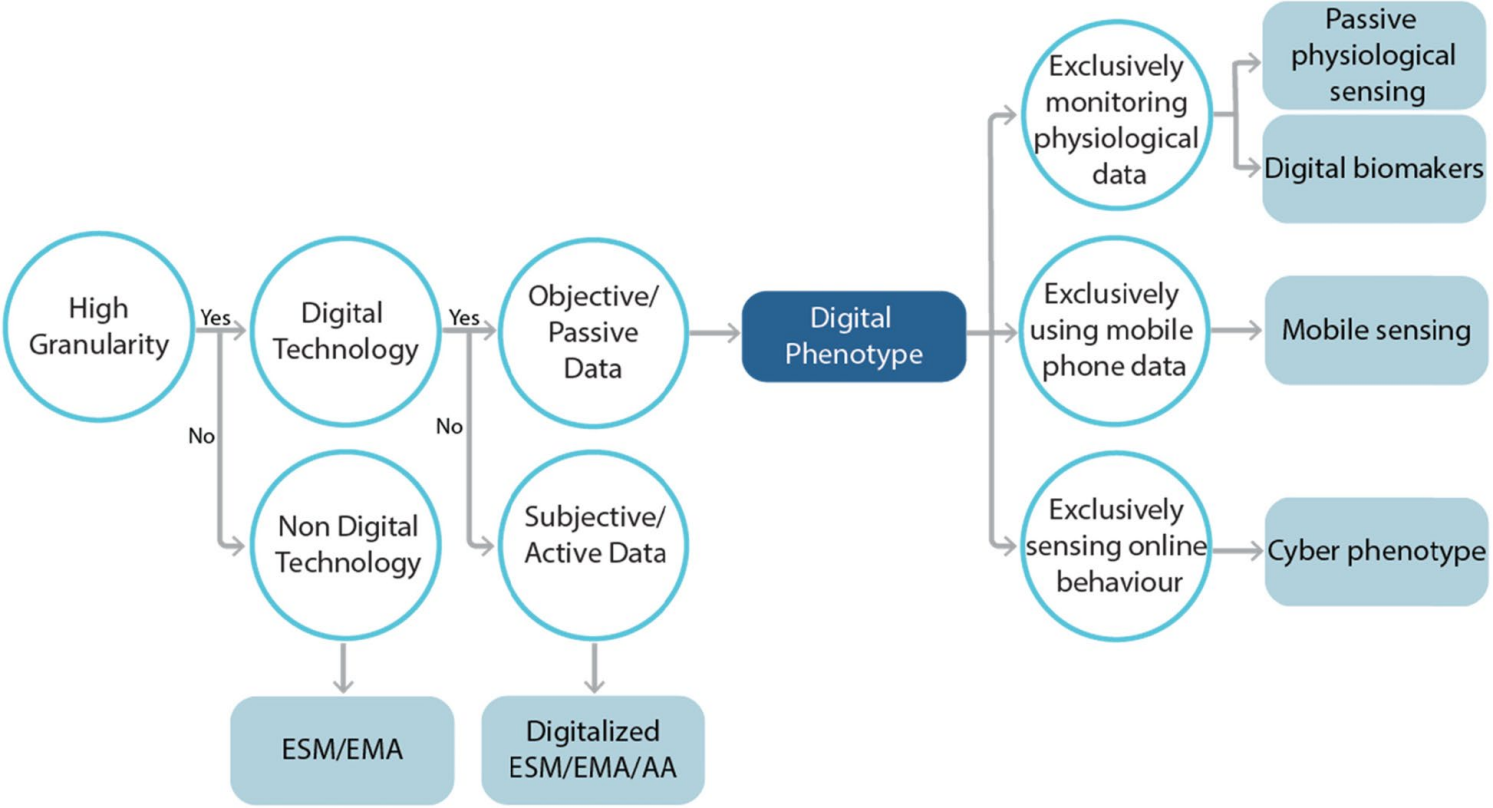
This Conceptual flowchart clarifies the current taxonomy within the field and provides guidelines suggesting how to used each related term. for example, while all these terms refer to methodologies with high granularity, some may employ digital technology, and some may not

The reviewed evidence indicates that EMA/ESM and ambulatory assessment may additionally communicate active/subjective involvement of participants through self-reported assessments and might not necessarily involve the use of digital technology (e.g., pen and paper surveys). Similarly, studies using terms such as ‘passive/mobile sensing’ referring to passive/objective data collection do not distinguish the behavior/data monitored (e.g., biological measures and online usage measures). Thus, addressing/clarifying the conceptual challenges observed in the field is compelling to: (a) improve the understanding of such methodologies; (b) increase future capacity to synthesize/integrate and compare empirical evidence; and (c) minimize the hindering of future research due to conceptual confusion. To contribute to this need, the findings of the present review suggest the concurrent consideration of aspects related to the level of granularity, objectivity/subjectivity, means, and nature of digital data collection implemented, to provide guidelines for the terms/definitions used (see Fig. 4).

All terms employed in the studies reviewed (e.g., ESM, EMA, DP, passive sensing, bio-sensing, etc.) appear to share the advantage of conducting in situ assessments in a *moment-to-moment* manner providing higher ecological validity [14, 20, 22, 60, 63, 145, 164, 179]. However, EMA, ESM and ambulatory assessment [AA] do not necessarily employ digital means (e.g., they may also use pen-and-paper), and even when they do so, they tend to *exclusively* involve active/subjective data collection (e.g., self-report questionnaires). Moreover, based on studies reviewed here, the DP necessarily assumes the use of digital means and the collection of objective/passive data without differentiating the type of technology (e.g., mobile phone/ wearable technology) or the nature of data used (e.g., biological measurements, behaviors, etc.). This aligns with the broadly understood definition of phenotype as the expression of one’s behavior or reactions objectively monitored/observed and not self-reported [42, 96]. Therefore, the inclusion of self-report measures under the umbrella of phenotyping is a contradiction and may be denominated as *active* DP, when digital means are used.

In this context, the reviewed findings suggest that DP may well conceptually operate as an *umbrella t*erm, inclusive of various methodologies, which differ on the specific means and nature of data collected (despite sharing high granularity and assuming digitally collected passive/objective data). In that context, *passive physiological sensing* may need to be introduced as a distinct form of DP emphasizing sensing/recording/monitoring one’s (externally observed) physiological activity/behavior (e.g., sleep, daily steps and distance moved). In contrast, *digital biomarkers*, as a complementary subtype of passive physiological sensing, may be considered to *exclusively* monitor internal biological measures (e.g., blood pressure; heartbeat). Similarly based on the content captured, *mobile sensing* could be viewed as *exclusively* monitoring mobile phone-derived information (e.g., SMS text, calls, GPS, actigraphy, etc.) and not any type of data collected via one’s phone. Finally, the term ‘cyber-phenotyping’ is supported to be introduced as an emerging subordinate DP theme to describe the in situ and continuous measurement/analysis of mental health digital footprints inferred *exclusively* from one’s cyber-behavior/ online usage (e.g., social media and online gaming). It is argued that although all these forms constitute passive sensing procedures, it is useful to be distinguished based on the distinct types of content they record, irrespective of the device used. Future technological progress is envisaged to generate new means of data collection that should not generate a similar inflation of terms regarding their usage in the mental health area. The proposed conceptual clarifications are expected to result in higher specificity in the use of the different terms and provide a ground for further elaboration in the field.

## Conclusion, limitations, and future directions

The present systematic literature review has significant theoretical and practical implications regarding the use of a person’s digital records to contribute to the assessment of depressive behaviors. Firstly, the integration of available empirical evidence highlights the viability and effectiveness of employing digital technology for the assessment/evaluation of depressive symptoms. Secondly, the current research trends observed in this review highlight important gaps inviting future research. Specifically, the assessment of dimensions of depression such as psychomotor activity or quality of sleep, the use of lifelong representative samples, and the validation of current knowledge in non-developed countries seem warranted. The potentially different response patterns across different population groups (e.g., individualistic vs. collectivistic cultures) may differentially relate to depressive presentations across populations compromising the comparability of findings (i.e., lack of measurement invariance [70]). Thirdly, high innovation and variability in relation to the data collection methods employed was observed. It is hoped that further opportunities will emerge with increased innovation and promotion of digitalization across different areas of every-day life.

Finally, it is suggested that refinement be made in reference to the terminology used to improve accuracy and specificity within this emerging research field. Indeed, a homogenous conceptual implementation of what is DP, and how it differs from other concepts/terms, may translate into higher unity/consistency in the field, alongside more commonly used terms that may be easier communicated to non-academic audiences. To this end, it is suggested that the term DP should be conceptualized as a superordinate term that is assumed to involve all instances where digital technology and objective passive data are used to examine a person’s health/mental health condition. Similarly, it is suggested that the term ‘active DP’ should be adopted to encompass all methodologies employing digital technology and the use of active/subjective data collection.

Despite these important findings, the present review also includes several limitations. Firstly, only studies published in English have been reviewed; thus, developments recorded in different languages may not have been captured. Secondly, only digital data-based studies related to depressive behaviors have been examined, and thus conclusions in relation to other presentations have not been addressed. Thirdly, while the use of digital technology may enable passive sensing of individual data to potentially increase the objectivity and ecological validity of depression-related symptoms, the possibility of *confirmation bias* exists [10]. For example, researchers attempting to minimize bias associated with traditional forms of assessments (such as interviews) may inadvertently favor methods enabling passive data collection. Fourthly, many of the studies included in the current review used the same or overlapping samples (e.g., [28, 130, 131] used the NESDA study); thus, caution should be exercised when interpreting findings presented herein. Additionally, the inclusion criteria utilized in the current search only required articles utilizing digital technology to assess depressive behaviors without necessarily identifying specific types of technology (e.g., GPS, actigraphy, mobile phone usage, etc.). Similarly, considering that the field of digital phenotyping is currently being published in a broad range of different journals with specific focus (e.g., informatics, medicine, medical informatics, computer science, engineering, etc.), many relevant journals may not have been covered by the databases included in the current review. Therefore, future research may benefit from a more refined search strategy to identify the use of DP related to selected types of technology, as well as targeting journals likely covering a broader collection of disciplines.

Nonetheless, in the context of these limitations, the present review constitutes a significant record considering the rapid advancement of the field, as well as the prospective opportunities and risks related to this highly promising intersection of mental health assessment with digital technology.

## Supplementary Information

**Additional file 1.** Prisma 2009 Checklist

**Additional file 2. **Supplementary Tables

## Data Availability

Studies included in the current review are publicly available, free to access (hyperlink in ‘references’ section) and included in the submission of the current manuscript.

## Footnotes

1 Standardizes measures include [9, 32, 39, 48, 61, 62, 64, 75, 78, 108, 111, 120, 127, 132, 156,159, 165, 199].

2 Studies assessing **mood** include [1, 2, 6, 8, 12, 15, 18, 21, 24, 27, 29, 30, 31, 33, 35, 37, 40, 45, 46, 50, 51, 66, 67, 71, 72. 73, 77, 79, 80, 82, 86, 87, 88, 89, 94, 100, 102, 103, 104, 105, 109, 114, 121, 122, 125, 130, 131, 135, 136, 138, 139, 141, 142, 144, 147, 148, 150, 151, 172, 173, 174, 181, 183, 186, 188, 190, 191, 192, 193, 194].

3 Studies assessing **psychomotor activity** include [6, 13, 15, 16, 29, 30, 37, 40, 49, 52, 79, 91, 92, 93, 94, 103, 104, 105, 125, 126, 128, 130, 131, 135, 135, 142, 152, 154, 162, 168, 170, 186, 187, 198, 200]. ^4^ Studies assessing **social functioning** include [8, 13, 24, 27, 30, 31, 49, 66, 79, 80, 93, 94, 126,136, 138, 152, 154, 163, 168, 188, 194].

4 Studies assessing **cognitive style** include [1, 2, 6, 33, 45, 58,82, 86, 87, 102, 106, 148, 150, 155, 173, 184, 191, 194, 197].

5 Studies assessing **sleep quality** include [6, 13, 21, 29, 49, 52, 56, 76, 88, 100, 105, 128, 131, 135, 138, 144].

6 Studies assessing **cognitive performance** include [24, 37, 88, 170, 187, 200].

7 Studies assessing **relationships between depression and variables of interest** included [8, 15, 16, 23, 25, 47, 53, 59, 65, 71, 76, 77, 83, 85, 97, 110, 122, 134, 143, 153, 157, 158, 160, 167, 171, 189, 196].
