# Correction: Exploring the digital footprint of depression: a PRISMA systematic literature review of the empirical evidence

**DOI:** 10.1186/s12888-022-04153-1

**Published:** 2022-08-05

**Authors:** Daniel Zarate, Vasileios Stavropoulos, Michelle Ball, Gabriel de Sena Collier, Nicholas C. Jacobson

**Affiliations:** 1 Institute for Health and Sport, Victoria University, Melbourne, Australia; 2 Department of Psychology, University of Athens, Athens, Greece; 3 Center for Technology and Behavioral Health, Geisel School of Medicine, Dartmouth College, Hanover, USA; 4 Department of Biomedical Data Science, Geisel School of Medicine, Dartmouth College, Hanover, USA; 5 Department of Psychiatry, Geisel School of Medicine, Dartmouth College, Hanover, USA; 6 Quantitative Biomedical Sciences Program, Dartmouth College, Hanover, USA

**Correction: BMC Psychiatry 22, 421 (2022)**

**https://doi.org/10.1186/s12888-022-04013-y**

Following the publication of the original article [1], the authors identified errors in the figures, and under the heading **Depressive behaviour digital traces***,* the footnotes and font size were incorrectly selected (Figs. 1 and 4).

**Fig. 1:**
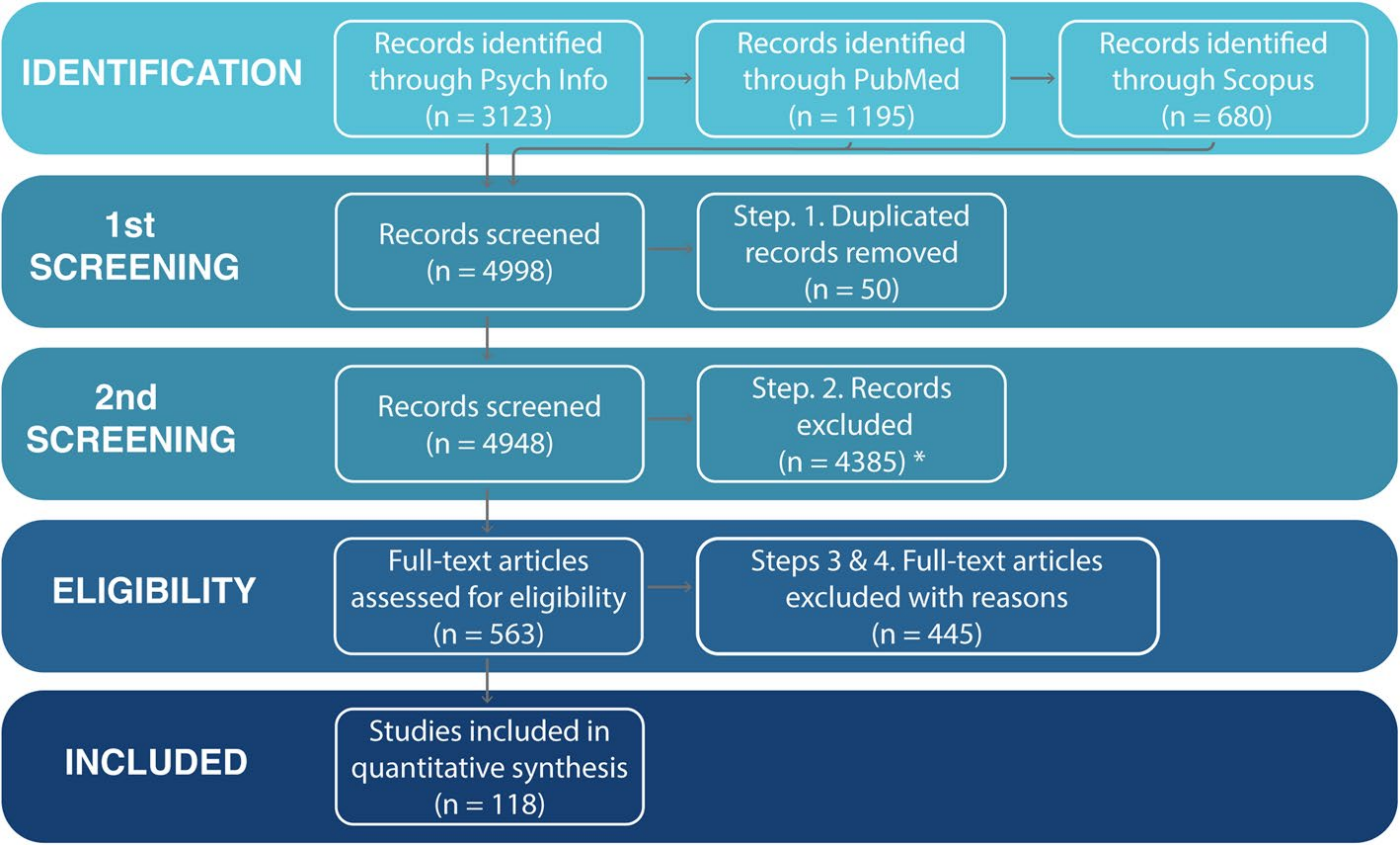
PRISMA flowchart of primary study selection. We excluded studies that exclusively called participants to conduct surveys over the phone given the limited ecological nature of such interventions. However, we have included studies that employed phone-based assessments where participants interact with pre-recorded messages. *Excluded if search terms were not targeted in the article. **Excluded if study i) did not use digital technology to conduct momentary assessments, ii) conducted psychometric evaluations of questionnaires

**Fig. 4:**
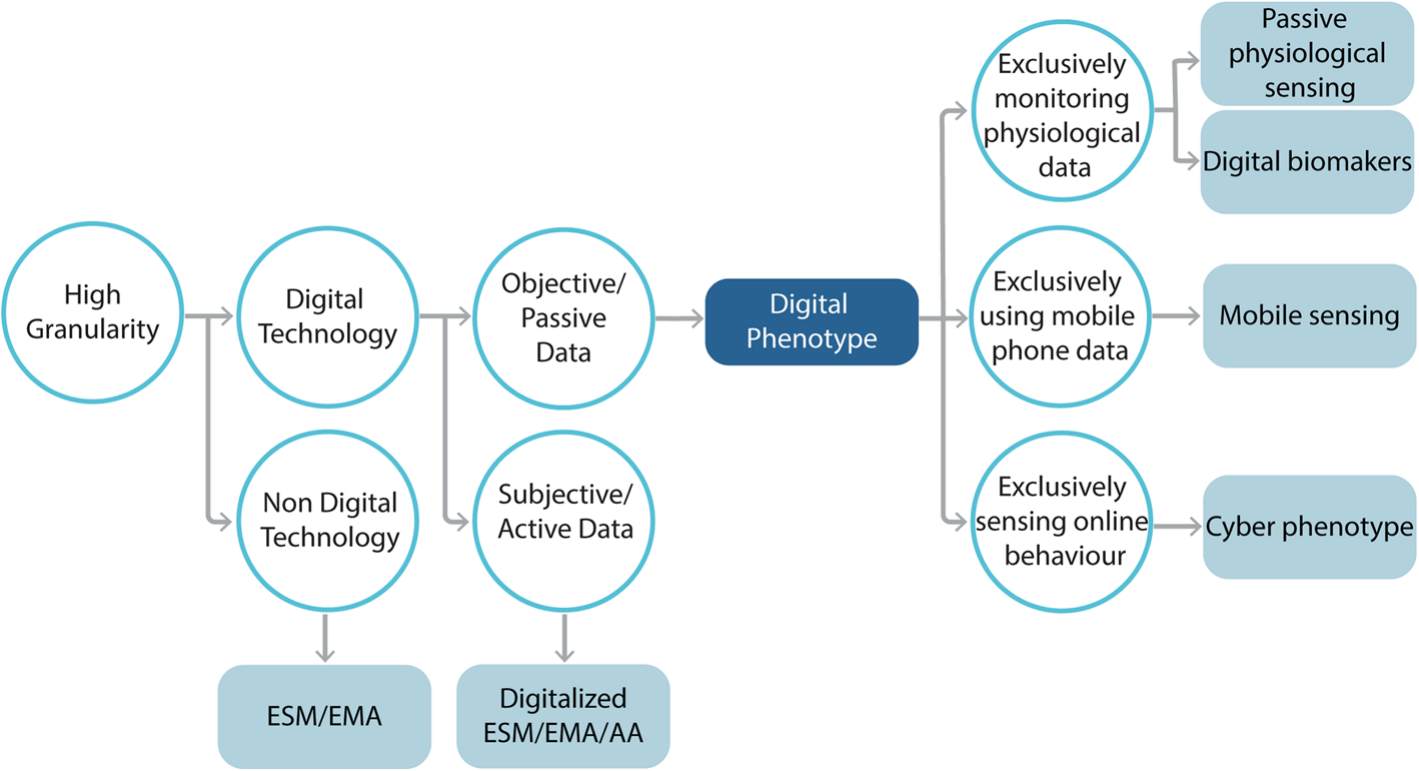
This conceptual flowchart clarifies the current taxonomy within the field and provides guidelines suggesting how to use each related term. For example, while all these terms refer to methodologies with high granularity, some may employ digital technology, and some may not

The original article [1] has been corrected.
