# Supplementary material for: Exploring the digital footprint of depression: a PRISMA systematic literature review of the empirical evidence
Source: BMC Psychiatry. 2022 Jun 22;22:421. doi: 10.1186/s12888-022-04013-y (PMC9214685; doi:10.1186/s12888-022-04013-y)
Supplement: Supplementary file 2 — Additional file 2. Supplementary Tables [file 12888_2022_4013_MOESM2_ESM.docx]

| \|  \|  \| Supplementary Table 1. Detailed Information of Studies, including Demographics, Measures employed to assess Depression-related Information and Major Findings. \| \| \| \| \| \| \| \| \| \| \| --- \| --- \| --- \| --- \| --- \| --- \| --- \| --- \| --- \| --- \| --- \| --- \| \| Study N. \| Study N. as listed in the ref. \| Author \| Title \| Country \| N \| Age range/ Mean \| White /  Male \| Population \| Duration \| Research Design \| Measures \| \| Findings \| \| 1 \| 1 \| Abela et al., 2007 \| Excessive reassurance seeking, self-esteem, and depressive symptoms in children of affectively ill parents: an experience sampling analysis. \| Canada \| 56 \| 6-14 / 10.6 \| 79% / 55% \| Children of parents with a history of depression. \| 6 weeks \| Quantitative, longitudinal \| SCID-I, CDI \| \| -Fluctuations in self-assessment of depressive symptoms was positively associated with baseline depression scores (*F* = 21.54, *p* < .001), the within-subject fluctuation in assessment of negative effects (*F* = 10.12, *p* < .01), and the interaction between reassurance seeking scores and fluctuations in assessment of negative effects (*F* = 6.44, *p* < .05). This suggest that those seeking reassurance and experiencing higher stress associated with events are more likely to develop depressive symptoms. \| \| 2 \| 2 \| Adams et al., 2009 \| Self-Criticism, Dependency, and Stress Reactivity: An Experience Sampling Approach to Testing Blatt and Zuroff’s (1992) Theory of Personality Predispositions to Depression in High-Risk Youth. \| Canada \| 56 \| 6-14 / 10.6 \| 79% / 55% \| Children of parents with a history of depression. \| 6 weeks \| Quantitative, longitudinal \| SCID-I, CDI \| \| -Fluctuations in self-assessment of depressive symptoms was positively associated with baseline depression scores (*F* = 30.64, *p* < .001), negative rating of daily events (*F* = 24.63, *p* < .001), the interaction between self-criticism and negative rating of daily events (*F* = 7.23, *p* < .01), and the interaction between children’s depressive experience and negative rating of daily events (*F* = 3.94, *p* < .05).  -Fluctuations in self-assessment of depressive symptoms was negatively associated with children’s depressive experiences (*F* = -5.89, *p* < .05). \| \| 3 \| 6 \| Bai et al., 2021 \| Tracking and Monitoring Mood Stability of Patients with Major Depressive Disorder by Machine Learning Models Using Passive Digital Data: Prospective Naturalistic Multicenter Study. \| China \| 334 \| 18-60 /-- \| --/-- \| Adults with major depressive disorder. \| 12 weeks \| Quantitative, longitudinal \| PHQ-9 \| \| -Predictive models with different combinations of features (i.e., phone usage, sleep quality, heart rate, and psychomotor activity) showed similar accuracy (ranging from 66% to 84%) in identifying whether participants had steady or variable depressive symptoms over time. Models best predicted comparisons between participants with low depressive symptoms and moderate variation.  -Random forest was (on average) the best machine learning algorithm for selected models compared to K nearest neighbour, support vector machine, Log regression, decision trees and naïve Bayes. \| \| 4 \| 8 \| Bartels et al., 2020 \| The necessity for sustainable intervention effects: lessons-learned from an experience sampling intervention for spousal carers of people with dementia. \| Netherlands \| 76 \| --/ 72.1 \| --/ 33% \| Spousal carers of people with dementia. \| 6 weeks / 6 months follow up \| Quantitative, longitudinal, randomized control trial \| CES-D \| \| -There was a significant interaction effect between treatment allocation (experimental, pseudo-experimental and controls) and time on depressive symptoms (*F* (6, 50) = 2.553, *p* = .028), suggesting that the treatment (face-to-face feedback with a coach and feedback) improved depressive symptoms.  -No significant differences in depressive symptoms between groups were observed at six months follow up. \| \| 5 \| 12 \| Ben-Zeev et al., 2009 \| Retrospective recall of affect in clinically depressed individuals and controls. \| USA \| 51 \| 18-65 / 37.7 \| 45% / 27% \| Adults with current diagnosis of major depressive disorder. \| 1 week \| Quantitative, longitudinal, non-randomized control trial \| BDI-II, SCID-I \| \| -A significant correlation between the variation in positive and negative mood (PA and NA) was observed in the depressed group (*r* = .21, *p* < .01), and not in the control group (i.e., healthy adults).  -Controls retrospectively exaggerated PA recall significantly more than NA (*t* (24) = 3.22, *p* = .014) but not the depressed group.  -While retrospective PA bias was greater than NA in depressed participants compared to controls, no significant differences were observed.  -Weekly NA average was a significant predictor of retrospective NA for controls (*F* (1, 43) = 6.72, *p* < .05) and not for depressed participants. This suggests that larger variations in NA were observed in depressed participants. \| \| 6 \| 13 \| Ben-Zeev et al., 2015 \| Next-Generation Psychiatric Assessment: Using Smartphone Sensors to Monitor Behavior and Mental Health. \| USA \| 47 \| 19-30 / 22.5 \| 47% / 79% \| (Under)graduate students. \| 10 weeks \| Quantitative, longitudinal \| PHQ-9 \| \| - Significant increase in depression symptoms (*p* < .001) over the course of the study.  - Speech duration (*p =* .048), geospatial activity (*p* =.022), and sleep duration (*p* = .028) were significant predictors of change in depression.  -Kinaesthetic activity not significantly associated with change in depression. \| \| 7 \| 15 \| Beute & de Kort, 2018 \| The natural context of wellbeing: Ecological momentary assessment of the influence of nature and daylight on affect and stress for individuals with depression levels varying from none to clinical. \| Netherlands \| 59 \| 20-60 / 33 \| 100% / 34% \| General population. \| 6 days \| Quantitative, longitudinal \| BDI-II \| \| - Models with either fixed or random effects for affect showed that depression was negatively correlated with hedonic tone (β = -.491; *p* < .001), and energy (β = -.193; *p* = .018), and positively correlated with tension (β = .259; *p* < .001).  - Depression was positively correlated with stress levels (β = .334; *p* < .001), rumination (β = .422; *p* < .001), and psychosomatic complaints (β = .258; *p* = .008), and negatively correlated with momentary health (β = -.432; *p* < .001). \| \| 8 \| 16 \| Bickham et al., 2015 \| Media use and depression: Exposure, household rules, and symptoms among young adolescents in the United States. \| USA \| 126 \| 12-15 / 14 \| 55% / 53% \| English speakers in grade 7 through grade 9. \| 1 year \| Quantitative, longitudinal \| BDI for Primary care \| \| - Depression was positively correlated at 1 year follow-up with TV use via EMAs (β = .206; *p* = .04), and with mobile phone use via EMAs (β = .223; *p* = .03)  - Depression was negatively correlated at 1 year follow-up with rules about TV (β = -.289; *p* = .04), and reaction to video games after controlling for baseline depression (β = -.298; *p* = .05) \| \| 9 \| 18 \| Bos et al., 2019 \| Affective variability in depression: Revisiting the inertia-instability paradox. \| Netherlands \| 100 \| 20-75 / 42.7 \| --/ 17% \| General population. \| 1 month \| Quantitative, longitudinal \| QIDS \| \| -Depression severity was positively associated with dispersion (Standard Deviation; *r* = .30, *p* = .003) and instability (RMSSD; *r* = .25, *p* = .011) of momentary affect.  -Mean levels of momentary negative affect were associated with depression severity (*r* = .68, *p* < .001).  -Inertia of affect (or resistance to change) and depression severity were not significantly associated.  -Hierarchical regression analyses determined that when controlling for mean levels of momentary affect, dispersion (β = .29, *p* = .004) and instability (β = .30, *p* = .004) were significant predictors of baseline depression severity. \| \| 10 \| 21 \| Bower et al., 2010 \| Poor reported sleep quality predicts low positive affect in daily life among healthy and mood-disordered persons. \| USA \| 96 \| --/ 28.3 \| 60% / 22% \| Adults diagnosed with major depression disorder. \| 3 days \| Quantitative, longitudinal \| BDI-II, SCID \| \| -Participants in the control group reported more positive affect (PA; *p* < .001) and less negative affect (NA; *p* < .001) compared with depressed participants.  -Severely depressed participants reported worse sleep quality (*p* < .001) compared with moderate depresses participants and controls, with higher depression severity related to lower PA (β = 0.42, *p* < 0.001) an not related to higher NA. \| \| 11 \| 23 \| Brose et al., 2017 \| Daily stressful experiences precede but do not succeed depressive symptoms: results from a longitudinal experience sampling study. \| Belgium \| 202 \| --/-- \| --/ 45% \| University students. \| 1 week / 1 year follow up \| Quantitative, longitudinal \| CES-D \| \| -Depressive symptoms after six months were significantly predicted by event intensity (β = .14, *p* < .05), stressed feelings (β = .10, *p* < .05), and depressed feelings (β = .13, *p* < .05). This suggests that participants experiencing more intense, stressful, and negative events, and more depressed feelings were more likely to develop depression. These relationships were observed in men and women. \| \| 12 \| 24 \| Brown et al., 2011 \| An Experience-Sampling Study of Depressive Symptoms and Their Social Context. \| USA \| 197 \| --/ 19.4 \| 72% / 24% \| University students. \| 1 week \| Quantitative, longitudinal \| BDI \| \| -There was a significant correlation between sex and depression severity (*r* = .21, *p* < .001), suggesting that female participants were more depressed than men.  -Depression severity was significantly predicted by decreased positive affect (*γ* = -.308, *p* < .001), event pleasantness (*γ* = -.162, *p* < .05), and ‘wellness’ (*γ* = .425, *p* < .001), and predicted by increased tiredness (*γ* = .291, *p* < .001) and negative affect (*γ* = .378, *p* < .001). There was also an interaction effect between negative affect and closeness, suggesting that depressed participants tended to feel less closeness as their negative affect increased.  -Depression severity was predicted by being alone (*γ* = -.032, *p* < .01), preferring to being alone (*γ* = .236, *p* < .001), and social distance index (*γ* = .171, *p* < .01). When participants were in company, those depressed scored lower in ‘liking’ (*γ* = -.142, *p* < .05) and ‘feeling close’ to the other person (*γ* = -.171, *p* < .05). When alone, depressed participants said they were alone because they were not wanted (*γ* = .200, *p* < .01), but preferred to be with others (*γ* = .236, *p* < .01).  -Depressed participants scored higher in the though impairment index (*γ* = .387, *p* < .001), had fewer clear thoughts (*γ* = -.397, *p* < .001), more concentration problems (*γ* = .378, *p* < .001), more suspicious thoughts (*γ* = .202, *p* < .01), disliked their activities (*γ* = -.215, *p* < .001), and preferred other activities (*γ* = .288, *p* < .001). \| \| 13 \| 25 \| Burns et al., 2011 \| Harnessing Context Sensing to Develop a Mobile Intervention for Depression. \| USA \| 8 \| 19-51 / 37.4 \| 87% / 12% \| English speakers with diagnosis of major depressive disorder. \| 8 weeks \| Quantitative, longitudinal, non-randomized  control trial \| MINI, QIDS-C, PHQ-9 \| \| -Self-reported depressive symptoms (via PHQ-9) decreased significantly over time (*t*_13_ = 7.02, beta_week_ = -.82, *p* < .001).  -Evaluator-rated depressive symptoms (via QIDS-C) decreased significantly over time (*t*_13_ = 8.22, beta_week_ = -.81, *p* < .001).  -Participants became less likely to meet diagnostic criteria for MDD after intervention (*Z* = 2.15, beta_week_ = -.65, *p* = .03). \| \| 14 \| 27 \| Bylsma et al., 2011 \| Emotional Reactivity to Daily Events in Major and Minor Depression. \| USA \| 99 \| --/ 28.3 \| 57% / 22% \| General population. \| 3 days \| Quantitative, longitudinal, non-randomized  control trial \| SCID, BDI-II \| \| -Participants with major and minor depression rated daily events as significantly (*p* < .05) less pleasant, more unpleasant, more stressful, less in control, less expected, lower frequency of positive events, and higher frequency of negative events when compared to controls.  -No significant differences between depressed participants and controls were observed for type of activity, location, and with whom they were interacting (except for significantly lower (*p* < .05) interaction with clients, customers, or students in the depressed groups).  -Similar relationships between variables were observed for observations conducted over one and three days. \| \| 15 \| 29 \| Cho et al., 2019 \| Mood Prediction of Patients with Mood Disorders by Machine Learning Using Passive Digital Phenotypes Based on the Circadian Rhythm: Prospective Observational Cohort Study. \| South Korea \| 55 \| -- / 25.9 \| -- / 51% \| Major depressive disorder and Bipolar patients. \| 2 years \| Quantitative, longitudinal \| Clinical interviews \| \| -Activity and light exposure during bedtime higher for High mood participants (HAMS) than low mood participants (LAMS)  -Total time sleep and sleep efficiency were not diff between HAMS and LAMS  -Regularity of sleep onset and offset were disrupted for HAMS and not for LAMS (regularity of sleep-wake cycle is closely related to mood state).  -Prediction of mood states in patients with MDD = 65% accuracy, 57% sensitivity, 68% specificity, and 0.69 AUC. Steps during bedtime was the most important predictive feature followed by deviation in sleep onset.  -Prediction of depressive episodes in patients with MDD = 71% accuracy, 41% sensitivity, 88% specificity, and 0.80 AUC. Light exposure during bedtime was the most important predictive feature followed by steps during bedtime. \| \| 16 \| 30 \| Chow et al., 2017 \| Using Mobile Sensing to Test Clinical Models of Depression, Social Anxiety, State Affect, and Social Isolation Among College Students \| USA \| 72 \| 18-23/ 19.8 \| 42/ 49% \| Undergraduate university students. \| 2 weeks \| Quantitative, longitudinal \| DASS-21 \| \| -There was a trend for individuals higher in depression to spend more time at home between 10:00am and 6:00pm, although the effect of depression on time spent-at home was non-significant during that window (β =.03, p=.06), as well as during 4-hour time intervals (β =.02, p=20)  -There was no main effect for depression on likelihood of spending time at-home (β =.14, p=.35), and unexpectedly depression was associated with lower likelihood of being at-home (β =-60, p=.001)  -There were no significant interactions between state affect and either depression or social anxiety in time spent at-home in same day short-windows (all p>.10), same day (all p>.10), previous day (all p>.10), and following day (all p>.10), when time spent at-home was measured both as a continuous or dichotomous variable. \| \| 17 \| 31 \| Chue et al., 2017 \| How Does Social Anger Expression Predict Later Depression Symptoms? It Depend on How Often One is Angry. \| USA \| 102 \| -- / -- \| 80% / 20% \| Undergraduate psychology students. \| 7 days / 4 month follow up \| Quantitative, longitudinal, non-randomized  control trial \| CES-D \| \| -Depression scores were positively correlated with mood reports via EMA – anger frequency (*r* = .32, *p* < .01), anger intensity (*r* = .29, *p* < .01), anxiety frequency (*r* = .37, *p* < .001), and sadness frequency (*r* = .51, *p* < .001).  - There was a significant interaction effect between reliance on social expression and anger frequency for depression scores at 4 months (i.e., follow-up) suggesting that high reliance on social expression of anger predicts a decrease in depression symptoms over time. \| \| 18 \| 33 \| Clasen et al.37, 2015 \| Mood-Reactive Self-Esteem and Depression Vulnerability: Person-Specific Symptom Dynamics via Smart Phone Assessment. \| USA \| 81 \| 19-55 / 28.7 \| 53% / 42% \| General population. \| 3 weeks \| Quantitative, longitudinal \| CES-D \| \| -Baseline depressive symptoms significantly predicted the likelihood of belonging to the mood reactive self-esteem group (*Z* = 2.15, *p* < .03). Once momentary assessment of rumination was included in the model it became the strongest predictor of mood reactive self-esteem group membership (*Z* = 2.80, *p* = .005), and baseline depression was no longer a significant predictor.  -Mood reactive self-esteem significantly predicted participants’ momentary depression (*t* = 4.55, *p* < .001). \| \| 19 \| 35 \| Colombo et al., 2020 \| Affect Recall Bias: Being Resilient by Distorting Reality. \| Spain \| 92 \| 11-36 / 22 \| --/ 25% \| Undergraduate students. \| 2 weeks \| Quantitative, longitudinal \| PHQ-9 \| \| -Retrospective bias in positive affect (PA) was negatively correlated with depression (*r* = -.440, *p* < .001) suggesting higher overestimation in PA for non-depressed participants.  - Retrospective bias in negative affect (NA) was positively correlated with depression (*r* = .291, *p* < .001) suggesting higher overestimation in NA for depressed participants.  -Together, PA and NA retrospective bias significantly predicted depression scores and explained 25% of the variance. \| \| 20 \| 37 \| Cormack et al., 2019 \| Wearable Technology for High-Frequency Cognitive and Mood Assessment in Major Depressive Disorder: Longitudinal Observational Study. \| USA \| 30 \| 19-63 / 37.2 \| --/ 36% \| Mild to moderate depressed adults taking medication for depression. \| 6 weeks \| Quantitative, longitudinal \| PHQ-9, semi structured interview \| \| -Depression scores were positively correlated with total mood scores (*r* =.69, *p* < .01), low mood (*r* =.56, *p* < .01), cognitive symptoms (*r* =.69, *p* < .01), and lack of interest (*r* =.70, *p* < .01).  -Depression scores were negatively correlated with mean values of cognitive performance task (*r* = -.38, *p* < .05).  -Gamification of tasks increased motivation – this improved cognitive task performance over time \| \| 21 \| 40 \| Cushing et al., 2018 \| Individual Differences in Negative Affectivity and Physical Activity in Adolescents: An Ecological Momentary Assessment Study. \| USA \| 26 \| 13-18 / 15.7 \| 69% / 58% \| Adolescents able to read and write in English. \| 20 days \| Quantitative, longitudinal \| POMS \| \| -A significant negative association was observed at the individual level between depressive mood and moderate to vigorous physical activity in the following 30 minutes window (β = -.13; *p* = .03), suggesting that depression depressive mood decreases after exercising.  -No associations were observed between depressive mood and moderate to physical activity in the previous 30 minutes. \| \| 22 \| 45 \| Dejonckheere et al., 2019 \| Poor emotion regulation ability mediates the link between depressive symptoms and affective bipolarity. \| Australia \| 100 \| --/ 24.1 \| --/ 23% \| General population. \| 2 weeks \| Quantitative, longitudinal \| CES-D, BDI-II \| \| -Emotion regulation ability significantly mediated the relationship between momentary affect and depression severity (measured either by CES-D or BDI-II).  -Rumination style significantly mediated the relationship between momentary affect and depression severity (measured either by CES-D or BDI-II). \| \| 23 \| 46 \| Demiralp et al., 2012 \| Feeling Blue or Turquoise? Emotional Differentiation in Major Depressive Disorder. \| USA \| 106 \| 18-40 / 27.8 \| --/ 28% \| Adults with Major Depressive Disorder. \| 1 week \| Quantitative, longitudinal, non-randomized control trial \| BDI-II \| \| -Depressed participants expressed significantly less differentiated negative emotions than controls (*F* [1, 98] = 7.18, *p* < .01) and no significant differences in differentiation of positive emotions.  -Gender was not a significant moderator for the relationship between depression severity and emotional differentiation.  -After controlling for intensity and variability of negative emotions, differentiation of negative (*F* [1, 96] = 6.53, *p* < .02) and positive emotions (*F* [1, 96] = 5.21, *p* < .03) were significant predictors of depression severity, \| \| 24 \| 47 \| Depp et al., 2015 \| Augmenting psychoeducation with a mobile intervention for bipolar disorder: A randomized control trial. \| USA \| 82 \| --/ 47.5 \| 69% / 41% \| Outpatient adults diagnosed with bipolar disorder I or II. \| 10 weeks / 24 weeks \| Quantitative, longitudinal, randomized control trial \| MADRS, MINI \| \| -Participants allocated to the intervention group (i.e., receiving personalised real time intervention for stabilising mood via smartphone; PRSIM) showed a not-significant group effect, suggesting that the type of intervention did not have a significant impact on depression scores. However, participants that scored >10 on the MADRs showed a significant effect for time (*F* (1, 30) = 6.7, *p* = .001) and a significant group*time interaction effect (*F* (3, 64) = 2.9, *p* = .038).  -There was no significant correlation between PRSIM intervention compliance and depression severity. \| \| 25 \| 49 \| Di Matteo et al., 2020 \| The Relationship Between Smartphone-Recorded Environmental Audio and Symptomatology of Anxiety and Depression: Exploratory Study. \| Canada \| 84 \| --/ 30 \| --/ 58% \| Canadian English speakers. \| 2 weeks \| Quantitative, longitudinal \| PHQ-8 \| \| -Depression scores were negatively correlated with daily similarity (*r* = -.37, *p* < .001) and speech presence ratio (*r* = -.37, *p* < .001) suggesting that depressed individuals experience irregular activity.  -Depression scores were positively correlated with sleep disturbance during weeknights (*r* = .23, *p* = .03) suggesting a link between depression and sleep quality. \| \| 26 \| 50 \| Di Matteo et al., 2021 \| Smartphone-Detected Ambient Speech and Self-Reported Measures of Anxiety and Depression: Exploratory Observational Study. \| Canada \| 86 \| --/ 30.1 \| --/ 57% \| Canadian English speakers. \| 2 weeks \| Quantitative, longitudinal \| PHQ-8 \| \| - Depression scores were correlated with the word *death* captured via audio features (*p* < .001 at Bonferroni adjusted α = .0002)  - The phrase *negative emotion* was most strongly and positively (but not significantly) correlated with depression scores compared to social anxiety, general anxiety, and impairment due to mental health.  - The phrase *positive emotion* was most strongly and negatively (but not significantly) correlated with depression scores compared to social anxiety, general anxiety, and impairment due to mental health. \| \| 27 \| 51 \| Dietvorst et al., 2021 \| Grumpy or depressed? Disentangling typically developing adolescent mood from prodromal depression using experience sampling methods. \| Netherlands \| 571  / 241 /286 \| 12-7/ 14.17 12-17/ 13.81  12-17/ 14.19 \| --/44.1%  --/37.8%  --/40.3% \| Dutch adolescents. \| Cross-sectional /  1 week ESM and 8-month follow-up \| Quantitative, mixed (cross-sectional and longitudinal) \| CDI-I,  CES-D \| \| -Three latent mood profiles were identified: “at-risk”, “typically developing”, and “happy”; based on their negative moods in three different contexts; school, friends and home.  -At-risk adolescents scored higher in depressive symptoms, followed by the typically developing profile and the happy profile across all samples.  -Typically developing and happy profiles were significantly different in terms of depression scores in S1A and S2, but not on S1B.  -At-risk mood profiles were significantly different in relation to their depressive symptoms in comparison with the typically developing profile across all samples.  -There was still a significant difference between at-risk and typically developing adolescents at the 3-month follow-up but not at the 7-month follow-up.  - At-risk adolescents more often than the happy and typically developing profiles, reported clinical levels of depression. \| \| 28 \| 52 \| Difrancesco et al., 2018 \| Sleep, circadian rhythm, and physical activity patterns in depressive and anxiety disorders: A 2-week ambulatory assessment study. \| Netherlands \| 359 \| --/ 49.5 \| --/ 36% \| Depressed adults. \| 2 weeks \| Quantitative, longitudinal, non-randomized control trial \| IDS \| \| -No significant differences in sleep duration or efficiency (as measured by actigraph) were observed between participants with current or remitted depression and controls.  -Depressed participants self-reported sleep duration and insomnia was significantly higher (*p* < .001) compared to those with remitted depression and controls.  -Depressed participants had lower circadian rhythm amplitude between daytime and night-time activity (*p* = .028), gross-motor activity (*p* = .021), and moderate-to-vigorous physical activity (*p* = .029) compared to controls. \| \| 29 \| 53 \| Eddington et al., 2017 \| The Effects of Psychotherapy for Major Depressive Disorder on Daily Mood and Functioning: A Longitudinal Experience Sampling Study. \| USA \| 55 \| --/ 37 \| --/ 20% \| Depressed adults. \| 1 week \| Quantitative, longitudinal, randomized control trial \| BDI-II, SCID \| \| -Participants in both treatment groups (self-system therapy and cognitive behavioural therapy) reported significant improvement in depression-related cognitions (*p* < .001), mood (*p* < .001), enjoyment of current activity (*p* < .001), physical functioning (*p* < .001), social functioning (*p* < .001), and appraisal of situation (*p* < .001).  -Time moderated the effect of positive situations only on two items (feeling sad and guilty), and items capturing depressive symptoms (feeling sad, out of control, bad about myself, guilty, irritable, overwhelmed, ruminating, trouble concentrating) in stressful situations. \| \| 30 \| 56 \| Elovainio et al.,  2020 \| Dynamic fluctuations of emotional states in adolescents with delayed sleep phase – A longitudinal network modelling approach. \| Finland \| 315 \| --/ 16.9 \| --/ -- \| Adolescents who are Finnish native speakers. \| 1 week \| Quantitative, longitudinal \| -- \| \| -Participants with delayed sleep phase (DSP) experienced significantly more feeling depressed (*p* < .001) than without DSP.  -Participants with DSP had a bidirectional loop between irritation and depression over time (0.12, *p* = .002).  -In individuals with DPS feelings of depression led to a decrease of happiness (-0.074, *p* = .032) and content (-0.100, *p* = .004), and an increase of irritation (0.09, *p* = .006) and anxiety (0.08, *p* = .005).  -In contemporaneous relationships (i.e., at a time point), DSP and non-DSP participants showed positive relationships between depression and anxiety, and depression and irritability. \| \| 31 \| 58 \| Fang et al., 2019 \| Do daily dynamics in rumination and affect predict depressive symptoms and trait rumination? An experience sampling study. \| Belgium \| 63 \| --/ 18.5 \| --/ 19% \| First year university students. \| 1 week / 6 weeks follow up \| Quantitative, longitudinal \| BDI-II \| \| -Momentary trait rumination did not significantly predict depression severity after six weeks.  -There was a non-significant tendency (*p* = .06) of depression severity to predict trait rumination at six weeks follow up.  -After controlling for baseline depression severity and trait rumination, affective variability (measured as entropy) predicted trait rumination at six weeks follow up (*p* < .05). \| \| 32 \| 59 \| Feiler et al., 2005 \| Using Interaction Graphs for Analysing the Therapy Process. \| Germany \| 57 \| --/ 51.4 \| --/ N.A. \| Female adults with fibromyalgia syndrome. \| 13 weeks \| Quantitative, longitudinal \| -- \| \| -Depression was significantly associated with pain, sleep, and self-efficacy (*p* < .01).  -The relationship between depression and sleep changed (i.e., became not-significant) after controlling for the effects of anxiety. \| \| 33 \| 65 \| Gansner et al., 2020 \| A pilot study using ecological momentary assessment via smartphone application to identify adolescent problematic internet use. \| USA \| 25 \| 12-23 / 15.5 \| --/ 32% \| Young adults receiving outpatient mental health care. \| 6 weeks \| Quantitative, longitudinal \| PHQ-9 \| \| -Not significant difference between depressed/non-depressed participants in reported increased awareness of the relationship between mental health and digital media use (*χ*^2^ = 2.88, *p* = .09).  -Not significant difference between depressed/non-depressed participants in PIU scores (*F* = 1.53, *p* = .14).  -Not significant difference in depression scores between PIU episodes or after PIU episodes (*F* = 2.40, *p* = .31) \| \| 34 \| 66 \| Geyer et al., 2018 \| I Did OK, but Did I like It? Using Ecological Momentary Assessment to Examine Perceptions of Social Interactions Associated with Severity of Social Anxiety and Depression. \| USA \| 60 \| 17-36 / 19.9 \| 40% / 45% \| Undergraduate psychology students. \| 2 weeks \| Quantitative, longitudinal \| DASS-21 \| \| -Not significant difference between participants being alone or in company (i.e., social situation) while completing EMAs and ratings of depression.  -Depression was significantly associated with negative affect (β = .14; *p* = .04), and not significantly associated with positive affect.  -No significant association between end of day ratings of perceived effectiveness in social interaction, enjoyment of social interactions and depression. Similarly, not significant relationships were observed when accounting for variability over time in EMAs (i.e., perceived effectiveness and social interaction).  -Significant interaction effect between perceived effectiveness and affect during social interactions for individuals with low and high depression scores (β = -.012; *p* = .002), indicating that depressed participants experienced more negative affect during social interactions and rated their interactions as less effective. Similarly, depressed participants experienced less positive affect and rated their interactions as less effective compared to non-depressed participants (β = .13; *p* = .001). \| \| 35 \| 67 \| Giesbrecht et al., 2012 \| Affective experience in ecologically relevant contexts is dynamic and not progressively attenuated during pregnancy. \| Canada \| 85 \| --/ 31.7 \| 91% / N.A. \| Women prior to 14 weeks gestation. \| 6 months \| Quantitative, longitudinal \| EPDS \| \| -Negative affect fluctuated similarly for depressed and non-depressed women (decreased between weeks 6 to 21 and increased between weeks 21 to 36). However, there was a significant intercept effect between depressed and non-depressed women (β = .215; *p* = .006).  -Positive affect (PA) fluctuation over time had a significant linear (β = .04; *p* < .05) and quadratic (β = -.001; *p* < .05) slope effect for depressed vs non-depressed women. That is, PA initially decreased (between week 6 and 21) and later increased (between week 21 and 36) for non-depressed women. Conversely, depressed women experienced an increase (weeks 6 to 21) and subsequent decrease (weeks 21 to 36) in PA. \| \| 36 \| 69 \| Goldschmidt et al., 2014 \| Affect and Eating Behavior in Obese Adults with and without Elevated Depression Symptoms. \| USA \| 50 \| 18-65 / 43 \| 76% / 16% \| Obese adults. \| 2 weeks \| Quantitative, longitudinal, non-randomized control trial \| BDI \| \| -Participants with higher depression scores had significantly greater body mass index (BMI; *t* = 2.3, *p* = .03).  -Depressed participants reported greater emotional eating (*F* (1, 50) = 4.3, *p* = .04), more frequent binge eating (Wald *χ*^2^ = 13.8, *p* < .001) and more negative affect (Wald *χ*^2^ = 7.7, *p* = .005) compared to controls.  -Emotional eating mediated the relationship between depressive symptoms and BMI (*p* < .05), and binge eating did not mediate the depression-BMI relationship. \| \| 37 \| 71 \| Graham-Engeland et al., 2016 \| Depressive Symptoms and Momentary Mood Predict Momentary Pain Among Rheumatoid Arthritis Patients. \| USA \| 31 \| --/ 50 \| 87% / N.A. \| Adults with rheumatoid arthritis. \| 7 days \| Quantitative, longitudinal \| CES-D \| \| -Significant relationships were observed between depression scores and pain (B = .09; *p* < .05), physical restrictions (B = .08; *p* < .05), positive mood (B = -.04; *p* < .05), and negative mood (B = .04; *p* < .05).  -Positive mood (B = -.17; *p* < .001), time of the day (B = -.11; *p* < .001), and negative mood (B = .14, *p* <.05) significantly predicted rheumatoid arthritis (RA) related pain in clinically depressed participants.  -Positive mood (B = -.16; *p* < .01), negative mood (B = .13, *p* < .05), weekend day (B = .34; *p* < .05) and time of day (B = -.08; *p* < .05) significantly predicted RA related physical restrictions in clinically depressed participants. \| \| 38 \| 72 \| Gruber et al., 2013 \| Real-World Emotion? An Experience-Sampling Approach to Emotion Experience and Regulation in Bipolar I Disorder. \| USA \| 84 \| 18-60 / 31.5 \| 94% / 34% \| Adults diagnosed with major depressive disorder or bipolar disorder. \| 6 days \| Quantitative, longitudinal, non-randomized control trial \| SCI, IDS \| \| -No significant differences were observed in positive emotionality (positive affect in reaction to specific events) across participants with bipolar (BD), major depressive disorder (MDD) or controls.  -BD and MDD participants scored similarly in negative emotionality but significantly higher compared to controls.  -Both BD and MDD reported significantly greater emotion regulation efforts compared to controls. \| \| 39 \| 73 \| Hahn et al., 2021 \| Early identification of postpartum depression using demographic, clinical, and digital phenotyping. \| Germany \| 501 \| --/ 32.1 \| --/N.A. \| Mothers giving birth. \| 12 weeks \| Quantitative, longitudinal, non-randomized control trial \| Clinical interview, HDRS, EPDS \| \| -Post-partum depression (PPD) was associated with personal (*p* < .001) and familial psychiatric history (*p* = .036).  -Higher occurrence of pre-menstrual syndrome was observed in women with PPD (*p* = .012) compared to controls.  -PPD group had significantly lower mood and higher stress compared to controls at all weeks except baseline.  -Evaluation of Edinburgh post-natal depression scores over time were best predictor of PPD compared to attachment and sociodemographic data. \| \| 40 \| 74 \| Hallensleben et al., 2017 \| Investigating the Dynamics of Suicidal Ideation. \| Germany \| 20 \| 23-58 / 35.9 \| --/ 20% \| Inpatients with unipolar depression. \| 6 days \| Quantitative, longitudinal \| SCID-I, DESC \| \| -Nonparametric correlations between fluctuations in suicidal ideation (SI) via mean squared successive difference (MSSD) and depression severity (DESC) was ρ = .362 (*p* = .117), and between SI and depressive episodes (SCID) was ρ = .069 (*p* = .780). Although non-significant, ρ values of > .30 suggest a relationship between measures of depression severity and suicidal ideation. \| \| 41 \| 76 \| Hamilton et al., 2020 \| Social media use predicts later sleep timing and greater sleep variability: An ecological momentary assessment study of youth at high and low familial risk for depression. \| USA \| 76 \| 9-13 / 11.3 \| --/ 54% \| Youth with parental history of psychopathology. \| 9 days \| Quantitative, longitudinal \| Clinical interview,  MFQ \| \| -No significant bivariate correlations were observed for depression and other variables (sleep onset mean, sleep onset variability, sleep duration mean, sleep duration variability, social media use, TV use, and videogame use).  -Multilevel regression models revealed no significant relationships between depression severity and variability in sleep timing and sleep duration. \| \| 42 \| 77 \| Hartmann et al., 2015 \| Experience Sampling-Based Personalized Feedback and Positive Affect: A Randomized Controlled Trial in Depressed Patients. \| Netherlands \| 102 \| 18-65 / 48 \| --/ 45% \| Pharmacologically treated outpatients with a diagnosis of major depressive disorder. \| 6 weeks \| Quantitative, longitudinal, randomized control trial. \| SCID-I, HDRS \| \| -Although the experimental group showed the largest increase in positive affect (PA) after the intervention, mean values across groups (i.e., experimental, pseudo-experimental and controls) were not significantly different (*p* = .846).  -There was a significant interaction effect between group and time (pre-post intervention) in negative affect (NA; *χ*^2^ (2) = 6.29, *p* = .043). This suggests that the experimental group showed greater decrease in NA after treatment compared to the experimental and control group. \| \| 43 \| 79 \| Heninga et al., 2019 \| The dynamical signature of anhedonia in major depressive disorder: positive emotion dynamics, reactivity, and recovery. \| Belgium \| 87 \| --/ 36.8 \| --/ 41% \| Adults diagnosed with major depressive disorder or bipolar disorder. \| 1 week \| Quantitative, longitudinal \| SCID \| \| -Anhedonic group did not experience lower frequency of psychological rewards (did something positive happen since the last beep?), nor a lower frequency of behavioural rewards compared to controls.  -Anhedonic group experienced lower levels of positive affect (PA; *p* < .001) and greater change in PA (*p* < .05), but did not experience more variability in PA, inert PA, or change in negative affect after behavioural rewards compared to controls.  -Anhedonic group did not return to baseline PA faster than controls after being with friends or doing an enjoyable activity. \| \| 44 \| 80 \| Hepp et al., 2019 \| Linking Daily-Life Interpersonal Stressors and Health Problems Via Affective Reactivity in Borderline Personality and Depressive Disorders. \| Germany \| 131 \| 18-65/ -- \| --/ 13% \| Adults with depression or borderline personality disorder. \| 28 days \| Quantitative, longitudinal \| SCID \| \| -Momentary negative affect (NA) was predicted by momentary interpersonal stress (IS; β = .012; *p* < .001), day level IS (β = .019; *p* < .001), and person level IS (β = .047; *p* < .001), but not by group membership (depressed vs borderline personality disorder).  -Depressive symptoms were predicted by momentary NA (β = .019; *p* < .001), day level NA (β = .08; *p* < .001), person level NA (β = .28; *p* = .032), momentary IS (β = .01; *p* = .048), day level IS (β = .03; *p* < .001), and interaction term between group and day level NA (β = .04; *p* < .001). \| \| 45 \| 82 \| Hershenberg et al., 2017 \| Anhedonia in the daily lives of depressed Veterans: A pilot report on experiential avoidance as a moderator of emotional reactivity. \| USA \| 50 \| --/56.5 \| 44% / 88% \| War veterans with a range of depression severity. \| 1 week \| Quantitative, longitudinal \| PHQ-9 \| \| -Depression status (depressed vs non-depressed) was negatively associated with positive affect (PA; *b* = -2.16, *p* = .002) and positively associated with negative affect (NA; *b* = 2.03, *p* < .001), accounting for 16.3% and 22.3% of the variance.  -Significant interaction effects were observed for event pleasantness, depression status and experiential avoidance considering PA (*b* = -.46, *p* = .01) and NA (*b* = .30, *p* = .02). This suggests that those with depression and high experiential avoidance experience less PA as the level of event pleasantness increases. Conversely, depressed participants with low experiential avoidance experience more NA as the level of event pleasantness increases. \| \| 46 \| 83 \| Holmes et al., 2016 \| Applications of time-series analysis to mood fluctuations in bipolar disorder to promote treatment innovation: a case series. \| UK \| 14 \| --/ 37 \| 100% / 14% \| Adults with bipolar disorder. \| 28 days / 28 weeks \| Quantitative, longitudinal, non-randomized control trial \| QIDS-SR \| \| -Significant reduction in depression severity was observed for pre and post treatment when baseline measurement of depression was computed as an aggregate of 4/5/6 weeks of pre-treatment assessment (*t* (13) = 3.86, *p* = .002) and computed as an aggregate of 28 days of pre-treatment assessment (*t* (13) = 2.99, *p* = .001).  -Markov chain analyses show that in 11 out of 14 patients there was a reduction in depression symptoms with a decreased occurrence in large depression scores (QIDS > 9). \| \| 47 \| 85 \| Huckins et al., 2020 \| Mental Health and Behavior of College Students During the Early Phases of the COVID-19 Pandemic: Longitudinal Smartphone and Ecological Momentary Assessment Study. \| USA \| 217 \| 18-22 / \| --/ 32% \| Undergraduate university students. \| 1 month \| Quantitative, longitudinal \| PHQ-4 \| \| -Depression scores spiked during COVID-19 weeks reaching a peak of 1.3 +SD compared to a peak of 1 +SD during normal academic terms.  -Modelling of the COVID-19 effects during the academic term revealed a significant increment in depressive symptoms (*p* < .001).  -Depressive symptoms were not significantly associated with an increasing proportion of COVID-19 news report. \| \| 48 \| 86 \| Huffziger et al., 2013(a) \| Effects of mood and rumination on cortisol levels in daily life: An ambulatory assessment study in remitted depressed patients and healthy controls. \| Germany \| 63 \| 18-55 / 44.9 \| --/ 33% \| Remitted depressed patients and control group. \| 2 days \| Quantitative, longitudinal, non-randomized control trial \| SCID-I, MADRS, BDI-II \| \| -No significant effects of depressive symptoms, brooding, and reflection were observed on momentary cortisol levels across remitted depressed patients and control group. \| \| 49 \| 87 \| Huffziger et al., 2013(b) \| Induced ruminative and mindful attention in everyday life: An experimental ambulatory assessment study. \| Germany \| 50 \| 19-31 / 22.9 \| --/ 40% \| Undergraduate university students. \| 3 days \| Quantitative, longitudinal \| BDI-II \| \| -Baseline depression levels did not predict immediate changes in ruminative self-focus or mood states on the days where participants were asked to focus on their feelings or accept their feelings.  - Baseline depression levels predicted changes throughout the day in ruminative self-focus (*B* = .3, *p* < .05) and mood polarity (*B* = -.23, *p* < .05). \| \| 50 \| 88 \| Hung et al., 2016 \| Smartphone-based ecological momentary assessment for Chinese patients with depression: An exploratory study in Taiwan. \| Taiwan \| 54 \| --/ 37.9 \| -- / 37% \| Chinese patients with depression. \| 8 weeks \| Quantitative, longitudinal \| HAM-D, PHQ-9 \| \| -Severity of depressive symptoms did not correlate with days of active use of app.  -Significant relationship between clinically assessed depression severity (HAM-D) and self-reported symptoms of depression (β = .53; *p* = .005), visual analogue scale of depression (β = .44; *p* = .003), anxiety (β = .53; *p* < .001) and poor sleep quality (β = .43; *p* = .023)  -Clinically assessed depression severity (HAM-D) was not significantly associated with sleep duration or errors in completing cognitive tasks. \| \| 51 \| 89 \| Husky et al., 2009 \| Past Depression and Gender Interact to Influence Emotional Reactivity to Daily Life Stress. \| USA \| 110 \| --/ 19.5 \| --/ 68% \| First year university students. \| 1 week \| Quantitative, longitudinal \| MINI \| \| -Female participants experienced higher momentary depressed mood (*γ* = .450, *p* < .01), and greater emotional reactivity to daily stressors (*γ* = .142, *p* < .001). And these differences were exacerbated in participants without prior history of depression compared to those with prior history of depression.  -Participants with prior depression were more emotionally reactive to stressful events (*γ* = .071, *p* < .05), but did not experience more depressed mood compared to participants with no previous history of depression. \| \| 52 \| 91 \| Jacobson et al., 2019 (a) \| Digital biomarkers of mood disorders and symptom change. \| USA \| 55 \| --/ 40.1 \| --/ 46% \| Patients diagnosed with mood disorders and control group. \| 2 weeks \| Quantitative, longitudinal, non-randomized control trial \| SCID, MADRS \| \| - Using actigraph features, there was a correct prediction of diagnoses 89% of the time (*Cohen’s kappa* = .773; *p* < .001).  -There was a significant correlation between predicted (using actigraph features) and actual change in depression severity (*r* = .78, *p* < .001). This indicates a strong prediction in patients’ depression severity in a period of up to two weeks. \| \| 53 \| 92 \| Jacobson et al., 2019 (b) \| Using Digital Phenotyping to Accurately Detect Depression Severity. \| Brazil / USA \| 15 \| --/ 47.6 \| --/ 13% \| Outpatients from mood disorder program. \| 1 week \| Quantitative, longitudinal \| HAM-D,  BDI-II, MINI \| \| -Strong correlation between observed (via actigraph) and predicted depression severity via BDI-II (*r* = .85, *p* < .001), and HAM-D (*r* = .60, *p* = .017). Controlling for false discovery rate did not affect observed significant relationships.  -After controlling for sex and age, the correlations between observed and predicted depression severity were significant (BDI-II, *r* = .91, *p* < .001; and HAM-D, *r* = .58, *p* = .039). \| \| 54 \| 93 \| Jacobson et al., 2020 (a) \| Digital Biomarkers of Social Anxiety Severity: Digital Phenotyping Using Passive Smartphone Sensors. \| USA \| 72 \| 18-23 / 19.8 \| 41% / 49% \| Undergraduate students. \| 1 week \| Quantitative, longitudinal \| DASS-21 \| \| -There was a significant positive correlation (*r* = .357, *p* = .005) between depression scores (assessed via DASS-21) and social anxiety disorder (SAD) symptoms assessed via a combination of accelerometer biomarkers, text and call biomarkers. This correlation was significantly lower (*Z* = 3.441, *p* < .001) compared to the correlation between depression and SAD scores assessed with validated measure (SIAS).  -Oscillations occurring every 6.35 seconds appeared to produce the most accurate representation of digital biomarkers. \| \| 55 \| 94 \| Jacobson et al., 2020 (b) \| Passive Sensing of Prediction of Moment-To-Moment Depressed Mood among Undergraduates with Clinical Levels of Depression Sample Using Smartphones. \| USA \| 31 \| 18-27 / 19.1 \| 68% / 35% \| Undergraduate students. \| 1 week \| Quantitative, longitudinal \| DASS-21, PANAS \| \| -Depressed mood scores assessed at baseline were highly correlated with modelled depressed scores including interindividual and intra-individual variability (*r* = .587).  -Looking specifically at correlations between baseline and modelled intraindividual depression scores resulted in significant correlations (average *r* = .376) for all but one participant (*r* = .18).  -Accounting for race determined non-significant effects, suggesting that race did not have an impact on the model’s predictive performance.  -The model continued to significantly predict depression scores when controlling for prior lagged depression. This indicates that results are not simply a carryover from the last measurement of depression scores. \| \| 56 \| 97 \| Jean et al., 2013 \| Daily Life Behaviors and Depression Risk Following Stroke: A Preliminary Study Using Ecological Momentary Assessment. \| France \| 36 \| --/ 63.31 \| --/ 53% \| Inpatients with first diagnosis of stroke. \| 3 months \| Quantitative, longitudinal \| HAM-D \| \| -Depression scores significantly decreased after 3 months in participants who reported being at their partner’s home (*γ* = -.210, *p* < .001) or a relative’s home (*γ* = -.147, *p* < .01) in the week after being discharged from hospital.  -Depression scores significantly increased after 3 months in participants who reported going to work (*γ* = .270, *p* < .05) and doing sports (*γ* = .127, *p* < .05) in the week after being discharged from hospital, while those reporting passive activities such as listening to music (*γ* = -.138, *p* < .05) reported significantly lower levels of depression. \| \| 57 \| 100 \| Kaufmann et al., 2016 \| Clinical significance of mobile health assessed sleep duration and variability in bipolar disorder. \| USA \| 41 \| --/ 46.9 \| 78% / 46% \| Outpatient receiving medication for bipolar disorder without disabilities. \| 11 weeks \| Quantitative, longitudinal \| MADRS \| \| -Self-reported depressive symptoms (i.e., how depressed are you?) was not significantly correlated with mean sleep duration.  -Self-reported SD of depression symptoms were significantly correlated with SD of sleep duration (ρ = .36, *p* = .021), suggesting that people with high variability in sleep duration also experienced high fluctuation in depressive moods.  -Participant who reported <5 hours (β = .27; *p* < .05) or >10 of sleep (β = .26; *p* < .05) experienced more depressive symptoms compared to participants who reported 7-8 hours of sleep. \| \| 58 \| 102 \| Khazanov et al., 2019 \| The “Brightening” Effect: Reactions to Positive Events in the Daily Lives of Individuals with Major Depressive Disorder and Generalized Anxiety Disorder. \| USA \| 145 \| --/ 32.70 \| 57% / 32% \| Adults with or without depression. \| 1 week \| Quantitative, longitudinal, non-randomized control trial \| DID, clinical interview \| \| -Mean ratings (i.e., positive to negative) of significant events since last EMA were significantly lower in clinical groups (i.e., participants with depression and anxiety scores above clinical thresholds) compared with control group (*F* (3, 141) = 13.78, *p* < .001).  -The mood brightening effect was observed as depressed participants reported significantly more positive affect (within group, *γ* = 1.20, *p* < .001; vs controls, *p* < .001), less negative affect (within group, *γ* = -0.60, *p* < .001; vs controls, *p* < .001), less rumination (within group, *γ* = -.65, *p* < .001; vs controls, *p* < .001), and less worry (within group, *γ* = -0.65, *p* < .001; vs controls, *p* < .001).  -Significant effects were observed at one signal (90 min) and two signals compared with control group (up to 180 min) after significant event. \| \| 59 \| 103 \| Kim et al., 2013 \| Co-Variation of Depressive Mood and Locomotor Dynamics Evaluated by Ecological Momentary Assessment in Healthy Humans. \| Japan \| 85 \| --/ 24.47 \| --/ 62% \| Healthy adolescents, undergraduate students, and office workers. \| 1 week \| Quantitative, longitudinal \| DAMS \| \| -Depressive mood was significantly correlated with fatigue (*r* = .26, *p* < .01) and anxious mood (*r* = .18, *p* < .01), and these significant correlations were consistent relationships across groups (adolescents, students, and office workers).  -Best fitting models to predict depression scores included local mean and detrended skewness of locomotor activity. The common feature observed in best fitting models was the intermittency of locomotor activity, characterised by a combination of decreased movement with occasional bursts of locomotor activity. \| \| 60 \| 104 \| Kim et al., 2014 \| Co-variation of Depressive Mood and Spontaneous Physical Activity Evaluated by Ecological Momentary Assessment in Major Depressive Disorder. \| Japan \| 14 \| 22-42 / 34 \| --/ 86% \| Patients with major depressive disorder. \| 37 days \| Quantitative, longitudinal \| HDRS, DAMS \| \| -Best fitting model identified with depressive mood scores as interaction between fixed and random effects.  -Skewness in locomotor activity was a significant predictor of depression scores (*γ* = 2.35, *p* = .013) suggesting an increased intermittent physical activity as depression scores increased. \| \| 61 \| 105 \| Kim et al., 2019 \| Depression Prediction by Using Ecological Momentary Assessment, Actiwatch Data, and Machine Learning: Observational Study on Older Adults Living Alone. \| South Korea \| 47 \| --/ 78 \| --/ 6% \| Older adults living alone in community settings. \| 2 weeks \| Quantitative, longitudinal \| HDRS \| \| -Depressed participants reported significantly lower mood scores (*p* = .004), reduced activity (*p* = .003), and interestingly, increased exposure to light (white, *p* = .008; red, *p* = .03; green, *p* = .005, and blue, *p* = .006) compared to non-depressed participants. Specifically, depressed participants were significantly more exposed to light (all colours) from 8am to 12pm and 4pm to 8pm compared to non-depressed participants.  -Sleep efficiency (measured via actigraph) did not differ across the depressed and non-depressed groups.  -Best fitting model predicting depression severity included self-reported mood, daily mean activity, light exposure from 4pm to 8pm, and daily sleep efficiency.  -Logistic regression model best predicted depression severity (accuracy = .91, precision = .93, specificity = .94) compared with boosted trees and random forest models. \| \| 62 \| 106 \| Kircanski et al., 2015 \| Rumination and worry in Daily Life: Examining the Naturalistic Validity of Theoretical Constructs. \| USA \| 70 \| 18-50 / 33.4 \| 61% / N.A. \| Adult women. \| 1 week \| Quantitative, longitudinal \| SCID, BDI-II \| \| -Level of rumination was predicted by perceived unpleasant thoughts (*t* = 8.33, *p* < .001, and significantly higher in the depressed group compared with the depressed and anxious group and controls), repetitiveness of thoughts (*t* = 7.85, *p* < .001), situational lack of control (*t* = 3.00, *p* < .01), uncontrollability (*t* = 2.94, *p* < .01), and past orientation (*t* = 2.62, *p* < .05).  -Level of worry was predicted by unpleasant thoughts (*t* = 3.23, *p* < .01), repetitiveness (*t* = 4.24, *p* < .001), abstractness (*t* = -3.27, *p* < .01), future orientation (*t* = 7.16, *p* < .001), verbal-linguistic focus (*t* = 3.84, *p* < .001), situational uncertainty (*t* = 5.11, *p* < .001), and situational control (*t* = -3.88, *p* < .001). \| \| 63 \| 109 \| Koval et al., 2013 \| Affect Dynamics in Relation to Depressive Symptoms: Variable, Unstable or Inert? \| Belgium \| 99 \| --/-- \| --/-- \| Undergraduate university students. \| 1 week \| Quantitative, longitudinal \| CES-D \| \| -Depression severity was positively correlated with momentary negative affect (NA; β = .62; *p* < .05), and negatively correlated with positive affect (PA; β = -.60; *p* < .05). Additionally, depression severity was positively correlated with variability in NA (β = .59; *p* < .05) and PA (β = .23; *p* < .05), inertia of NA (β = .34; *p* < .05), and instability of NA (β = .54; *p* < .05) and PA (β = .23; *p* < .05). \| \| 64 \| 110 \| Kramer et al., 2014 \| A therapeutic application of the experience sampling method in the treatment of depression: a randomized controlled trial. \| Netherlands \| 102 \| 18-65 / 48 \| --/ 82% \| Depressed outpatients attending mental health care facilities \| 7 weeks / 32 weeks follow-up \| Quantitative, longitudinal, randomized control trial \| HDRS, IDS-R \| \| -The experimental group (feedback via ESM only) showed significantly greater weekly decline in depressive symptoms compared to controls (HDRS: *B* = -.15, *p* < .001; IDS:  *B* = -.29, *p* = .002). This decline became significant at week 8 (IDS) and 11 (HDRS) and lasted until the end of the study.  -The pseudo experimental group (feedback and treatment as usual) experienced an initial decline in depressive symptoms that did not persist over time and became not-significant after weeks 26 (HDRS) and 28 (IDS). \| \| 65 \| 114 \| Lavender et al., 2013 \| Associations between retrospective versus ecological momentary assessment measures of emotion and eating disorder symptoms in anorexia nervosa. \| USA \| 116 \| --/ 25.4 \| --/ N.A. \| Women with anorexia nervosa. \| 2 weeks \| Quantitative, longitudinal \| BDI \| \| -Depression severity was significantly correlated with EMA affective lability (*r* = .27, *p* < .01), clinical dimensional assessment of personality pathology (DAPP; *r* = .51, *p* < .001), EMA anxiousness (*r* = .45, *p* < .001), DAPP anxiousness (*r* = .60, *p* < .001), and eating disorder clinical examination (*r* = .61, *p* < .001).  -Hierarchical regressions determined that depression severity was the strongest and significant predictor of eating disorders and dietary restriction compared with EMA anxiousness, DAPP anxiousness, EMA affective lability, DAPP affective lability, eating disorder diagnostic, age, and body mass index. \| \| 66 \| 121 \| Maher et al., 2018 \| Mean Level of Positive Affect Moderates Associations between Volatility in Positive Affect, Mental Health, and Alcohol Consumption among Mothers. \| USA \| 202 \| 24-27 / 40.9 \| 50% / N.A. \| Working mothers with young children. \| 8 days \| Quantitative, longitudinal \| CES-D \| \| -Depression symptoms were negatively correlated with positive affect (β = -.35; *p* < .001).  -The interaction between variability of positive affect and depression symptoms were positively correlated with mean values of positive affect (β = .20; *p* = .02). Specifically, low variability and high mean of positive affect was observed in participants with little depressive symptoms. Conversely, high variability and low mean of positive affect was observed in participants with high depressive symptoms. \| \| 67 \| 122 \| Mak & Schneider 2020 \| Individual differences in momentary pain-affect coupling and their associations with mental health in patients with chronic pan. \| USA \| 106  68  116 \| --/ 55.3  --/ 50.9  --/ 57.4 \| 91%/ 14%  94% / 15%  96%/ 15% \| Study 1 = Adults with chronic rheumatic disease.  Study 2 = Chronic pain sufferers with specific rheumatic disease.  Study 3 = Chronic pain sufferers from a community rheumatology practice. \| 29-31 days  1 week  1 week \| Quantitative, longitudinal  Quantitative, longitudinal  Quantitative, longitudinal \| BDI-II  BDI-II  BDI-II \| \| -Correlation for pain-negative affect and depression relationships were significant for all studies (*r* = .31, *p* < .001).  -Fixed-effects meta-analysis reported a not significant correlation between lagged pain-to-negative affect and depression (*r* = .14, *p* = .17).  -Fixed-effects meta-analysis reported a not significant correlation between lagged pain-to-positive affect and depression (*r* = -.11, *p* = .26).  -Results from meta-analysis (i.e. all three studies) reported that neither the pain-negative affect association (β = .02, *p* = .86), nor the pain-positive affect association (β = -.14, *p* = .13) predict depressive symptoms after controlling for individual differences in means, variability, residual autocorrelation, and time trends. Similarly, time lagged affective associations with pain resulted in not significant predictions of depressive symptoms after controlling for individual differences in means, variability, residual autocorrelation, and time trends. \| \| 68 \| 125 \| Mata et al., 2012 \| Walk on the Bright Side: Physical Activity and Affect in Major Depressive Disorder. \| USA \| 106 \| --/ 26.8 \| 68% / 30% \| Adults diagnosed with major depressive disorder. \| 7-8 days \| Quantitative, longitudinal \| SCID, BDI \| \| -Control group showed higher average positive affect (PA) compared to depressed group (*t* (103) = 5.67, *p* < .001) excluding physical activity measures. Both groups showed similar PA when physical activity was included.  -There was no change in negative affect (NA) as a function of physical activity across groups (depressed vs control).  -Both groups showed increased PA as a function of length of physical activity (*t* (905) = 2.58, *p* = .01) whit greater (and not significant) increase on PA in the depressed group compared to controls. No effects were observed in NA as a function of length of physical activity.  -There was increase in PA (at the day level) when participants were active compared to inactive, and this effect was greater (but not significant) for depressed group compared with controls.  -Control group showed significantly less NA on inactive days (*t* (103) = -9.64, *p* < .001 and similar NA on active days compared to depressed participants, \| \| 69 \| 126 \| McIntyre et al., 2021 \| Ecological momentary assessment of depressive symptoms using the mind.me application: Convergence with Patient Health Questionnaire (PHQ-9). \| Canada \| 200 \| 18-65 / 46 \| --/ -- \| Adults who self-identified as having clinically significant depressive symptoms. \| 90 days \| Quantitative, longitudinal \| PHQ-9 \| \| -Model including self-reported depression scores (i.e., PHQ-9), GPS derived data, SMS count and call count was used to identify depressed participants (with confirmed MDD diagnosis by health-care provider). The model showed a 91% accuracy with 98% sensitivity and 93% specificity. \| \| 70 \| 128 \| Melcher et al., 2021 \| Digital phenotyping of student mental health during COVID-19: an observational study of 100 college students. \| USA \| 100 \| 18-27 / 20.3 \| 63% / 24% \| Undergraduate students \| 28 days \| Quantitative, longitudinal \| PHQ-9, DASS-21, HDRS \| \| -PHQ-9 showed high correlation with self-rated depressive symptoms (ρ = .84)  -Sleep (measured in hours via accelerometer) was not significantly related to depression severity.  -No significant relationships were found between time spent at home and depression severity.  -Sleep variance (derived from accelerometer) was positively correlated with depression severity (ρ = .28), suggesting that participants with irregular sleep patterns experienced more symptoms of depression. \| \| 71 \| 130 \| Minaeva et al., 2020a \| Screening for Depression in Daily Life: Development and External Validation of a Prediction Model Based on Actigraphy and Experience Sampling Method. \| Netherlands \| 125/ 54 \| 18-65 / 52 / 34.3 \| --/ 40%  --/ 26% \| Adults with depressive disorders. \| 2 weeks \| Quantitative, longitudinal \| BDI-II, IDS \| \| -The model including self-reported momentary assessment of depressed affect and behaviour showed 95.2% predictive capacity. The model including actigraphy data showed 71.8% predictive capacity. Combining both models (self-reported assessments and actigraphy data) resulted in 95.2% predictive capacity.  -Both models showed similar area under the receiver operating characteristic curve (AUC) for development and validation dataset, with self-reported momentary assessment development AUC = .99, and validation AUC = .89, actigraphy model development AUC = .79, and validation AUC = .65, and both models combined development AUC = .99, and validation AUC = .89. \| \| 72 \| 131 \| Minaeva et al., 2020b \| Level and timing of physical activity during normal daily life in depressed and non-depressed individuals \| Netherlands \| 121 \| 28-72/ 52.1 \| --/36% \| General adult sample (mix between depressed individuals and healthy controls) \| 2 weeks \| Quantitative, longitudinal \| IDS \| \| -No significant differences in gender, age, marital status, BMI, and chronotype were found between the total depressed groups and controls. In addition, depressed individuals were more often unemployed, smokers, had lower education, higher mean depressive symptom scores, and used more medication compared to controls. This was similar in the acute depression group compared to controls.  -Effect sizes for the acute depression groups were larger than in the total depression group across activity mean levels (MESOR), the difference between peak and mean level (amplitude), and the timing of the activity peak (acrophase).  -In comparison to the healthy control group, both depression groups (total: β = −0.003, *p* = 0.033; acute: β =  −0.004, *p* = 0.005) had lower levels of physical activity. Amplitude was also reduced, but only in the acute depression group (total: β = −0.002, *p* = 0.065; acute: β = −0.003, *p* = 0.011). Similarly, the timing of activity was marginally significant towards a later timing of activity in the acute, but not total depression group (total: β = 0.206, *p* = 0.398; acute: β = 0.405, *p* = 0.084). \| \| 73 \| 134 \| Moreno et al., 2012 \| Depression and Internet Use among Older Adolescents: An Experience Sampling Approach. \| USA \| 189 \| 18-23 / 18.9 \| 90% / 41% \| Older adolescents (students). \| 1 week \| Quantitative, longitudinal \| PHQ-9 \| \| -There were no significant differences in depression scores across groups (low, medium and high internet use). However, adjusting the high internet use criteria from 2 to 3 hours per day, resulted in significantly higher depression scores for this group best represented by a quadratic trend (*p* = .004). \| \| 74 \| 135 \| Moshe et al., 2021 \| Predicting Symptoms of Depression and Anxiety Using Smartphone and Wearable Data. \| Finland \| 60 \| 24-68/ \| 93 % / 45% \| Healthy English speaker adults. \| 31 days \| Quantitative, longitudinal \| DASS-21 \| \| -Severity of depressive symptoms was correlated with GPS features (location variance, *r* = -.31, *p* = .035; location entropy, *r* = -.3, *p* = .035; but not correlated with total distance, normalised location entropy, and homestay).  -Severity of depressive symptoms was correlated with self-reported mood (arousal, *r* = -.30, *p* = .003; and valence *r* = -.48, *p* < .001).  -Severity of depressive symptoms was not correlated with smartphone usage features (time and frequency) or wearable device data (steps, metabolic equivalent for task, total sleep time, sleep onset latency, wake after sleep onset, time in bed, heart rate variability).  -Multilevel model determined that including self-reported mood, GPS derived data, and wearable derived data (i.e., heart rate) significantly improved prediction of depression severity. \| \| 75 \| 136 \| Moukaddam et al., 2019 \| Findings from a Trial of the Smartphone and Online Usage-based evaluation for Depression Application \| USA \| 22 \| 22-66 / 50.3 \| 41% / 76% \| Adults with diagnosis of Major Depressive Disorder. \| 8 weeks \| Quantitative, longitudinal \| PHQ-9, HAM-D \| \| -Sig correlation (*p* < .05) between self-evaluated mood (through app) and severity of depressive symptoms (PHQ-9, HAM-D)  -Depression scores for the moderately to severely depressed group (PHQ-9) were negatively correlated with frequency of SMS (*r* = -.51, *p* < .05), SMS length (*r* = -.57, *p* < .01), daily steps taken (*r* = -.69, *p* < .01), self-reported daily mood levels (*r* = -.62, *p* < .01), and current mood level (*r* = -.59, *p* < .01). PHQ-9 scores were not correlated with daily call duration.  -Depression scores for the moderately to severely depressed group (HAM-D) were negatively correlated with daily mood level (*r* = -.71, *p* < .01) and current mood level (*r* = -.59, *p* < .01), and not correlated with daily SMS count, average SMS length, daily steps, daily call counts, and daily call duration.  -Regression model using app collected info (daily mood, current mood, call freq., call duration, SMS freq., SMS duration, screen on %) correlated with depression scores, with higher correlation for moderate to severe scores. \| \| 76 \| 138 \| Narziev et al., 2020 \| STDD: Short-Term Depression Detection with Passive Sensing. \| South Korea \| 20 \| --/ -- \| --/ -- \| Undergraduate students. \| 4 weeks \| Quantitative, longitudinal \| PHQ-9, SCID, BDI-II \| \| -Sleep represented the highest contributing variable to model predictability (35%), followed by physical activity (23%), mood (20%), social activity (13%) and food intake (10%). Specifically, motion and step features were the most important variables within physical activity, and heart rate sensor the most important feature for mood detection.  -Depression related symptoms such as psychomotor retardation, social activity or sleep showed similar ratings whether these were collected via self-reports or passive sensing (i.e., heart rate).  -Model predictive accuracy was not reported. \| \| 77 \| 139 \| Nelson et al., 2018 \| Everyday Emotional Dynamics in Major Depression. \| Germany \| 80 \| --/ 34.6 \| --/ 37% \| Depressed adults and healthy control group. \| 4 days \| Quantitative, longitudinal, control trial \| BDI-II \| \| -Positive (PA) and negative (NA) affect significantly differed between depressed and control group. The depressed group showed lower PA mean (*γ* = -34.34, *p* < .001) explaining 42% of the total variance, and higher NA mean (*γ* = 14.96, *p* < .001) explaining 23% of the total variance.  -Emotional inertia (based on the ratings of events and activities; *t* = 2.75, *p* = .04), emotional variability (*t* = 7.50, *p* < .001), and emotional reactivity of positive activities (*t* = -3.62, *p* < .001) had significant effects on NA (and not on PA) for depressed participants. \| \| 78 \| 141 \| Nook et al., 2021 \| High Emotion Differentiation Buffers Against Internalizing Symptoms Following Exposure to Stressful Life Events in Adolescence: An Intensive Longitudinal Study. \| USA \| 30 \| 15-17/-- \| --/ N.A. \| Female adolescents. \| 12 week spread across four 3-week waves) \| Quantitative, longitudinal \| PHQ-9 \| \| -There were significant within-persons and between-persons positive associations between depressed affect and depression symptoms with all other measures (perceived stress, stress impact scores, anxious affect, anxiety symptoms, negative emotion differentiation, and positive emotion differentiation).  -Within-persons fluctuations in perceived stress were significantly linked with depressed affect, such that when adolescents reported higher stress than usual, they also reported higher depressed affect than usual. This was stronger in participants with low negative emotion differentiation (β = .38, *p* < .001).  -Within-persons fluctuations in perceived stress and depressed affect were also moderated by positive emotion differentiation.  -The relationship between within-persons fluctuations in stress impact scores and symptoms of depression at the monthly-level was non-significant.  -Although anxious affect was a strong predictor of depressed affect (β = .38, SE = 0.01, t = 27.65, *p* < .001), negative emotion differentiation significantly moderated this association (β = −0.07, SE = 0.01, t = −4.96, *p* < .001), such that anxious and depressed effect was more strongly related in participants with low negative emotion differentiation, in comparison with participants with high differentiation scores. \| \| 79 \| 142 \| Nylocks et al., 2019 \| Testing the influence of negative and positive emotion on future health-promoting behaviours in a community sample. \| USA \| 56 \| 18-65 / 33.8 \| 89% / 23% \| Depressed adults \| 2 weeks \| Quantitative, longitudinal \| SCID, CES-D \| \| -Positive affect predicted engagement in positive health behaviours such as doing a hobby or practicing sports in depressed and non-depressed groups (*B* = .25, *p* = .01).  -Group membership (depressed vs non-depressed) did not predict positive health behaviours. \| \| 80 \| 143 \| Odgers & Russell 2017 \| Violence exposure is associated with adolescents’ same- and next-day mental health symptoms. \| USA \| 151 \| 11-15 / 13 \| 57% /-- \| Adolescents at risk for exposure to violence and mental health problems. \| 30 days / 18 month follow up \| Quantitative, longitudinal \| BDI \| \| -Adolescents who are frequently exposed to violence had significantly higher odds of experiencing depression (β = 1.66, *p* < .001) and other difficulties (i.e., anger, irritability, conduct problems and health-risk behaviour).  -Person-level relationships showed that adolescents more frequently exposed to violence had significantly higher odds of experiencing depression (β = 1.03, *p* < .01) and other difficulties.  -Exposure to violence was significantly associated with experiencing depressive symptoms the following day (β = 2.57, *p* < .001). \| \| 81 \| 144 \| O’Leary et al., 2017 \| Sleep quality in healthy and mood- disordered persons predicts daily life emotional reactivity. \| USA \| 96 \| --/ 28.5 \| 60% / 22% \| Adults with major or minor depressive disorder. \| 3 days \| Quantitative, longitudinal \| SCID, BDI-II \| \| -Negative and neutral event appraisal were predicted by group membership (with depressed participants reporting higher negative rates; *B* = 9.84, *B* = 9.89, *p* < .001), and not predicted by self-reported quality of sleep. However, group membership and quality of sleep had a significant interaction effect on negative and neutral event appraisal (*B* = 3.15, *B* = 3.04, *p* < .05), with depressed participants reporting more negative affect in unpleasant and neutral events as sleep efficiency decreased.  -Negative and neutral event appraisal were predicted by group membership (*B* = 10.38, *B* = 10.32, *p* < .001) and sleep disturbances (*B* = -3.95, *B* = -4.09, *p* < .01). Group membership and sleep disturbances had a significant interaction effect on negative and neutral event appraisal (*B* = 6.19, *B* = 6.40, *p* < .001), with depressed participants reporting more negative affect in unpleasant and neutral events as sleep disturbances increased. \| \| 82 \| 147 \| Panaite et al., 2018 \| The role of appraisal in dysphoric affect reactivity to positive laboratory films and daily life events in depression. \| USA \| 74 \| 18-58 / 23.01 \| 67% / 24% \| Adults with Major Depressive Disorder. \| 3 days \| Quantitative, longitudinal \| BDI \| \| -Depressed participants reported higher dysphoric affect (DA; *t* = -10.37, *p* < .001), and larger decrease in DA for positive events (*B* = -.77, *p* = .009) compared to controls.  -Depressed participants appraised positive films and events similarly to controls. However, they rated neutral films and events as less important (*t* = 2.47, *p* = .016; *B* = -.51, *p* = .023) and less pleasant (*t* = -3.10, *p* = .003; *B* = -.44, *p* = .007), and more unpleasant films (*t* = -3.10, *p* = .003) but equally unpleasant events compared to controls. \| \| 83 \| 148 \| Panaite et al., 2019 \| Emotion regulation and mood brightening in daily life vary with depressive symptom levels. \| USA \| 95 \| --/ 19.1 \| --/ 38% \| Undergraduate university students. \| 1 week \| Quantitative, longitudinal \| CES-D \| \| -Depressed participants reported higher mean levels of negative affect (NA; *B* = 13.74, *p* < .001) compared to controls, including higher mean levels of momentary sadness, anger, and anxiety.  -Depressed participants were more likely to engage in rumination (*B* = 18.75, *p* < .001), expressive suppression (*B* = 12.23, *p* < .001), and distraction (*B* = 8.25, *p* = .017), but not in reflection, reappraisal, and social sharing.  -Results showed that positive events varied as a function of depressive symptoms, such that those with higher depression experienced larger decrease in negative affect (*γ* = -4.75, *p* <.001), sadness (*γ* = -5.07, *p* = .001), and anxiety (*γ* = -4.93, *p* < .001) as a reaction to positive events. \| \| 84 \| 150 \| Pasyugina et al., 2015 \| Distinguishing between level and impact of rumination as predictors of depressive symptoms: An experience sampling study. \| Belgium \| 101 \| 18-31 / 21.4 \| --/ 26% \| Native Dutch speakers who are university students. \| 9 days \| Quantitative, longitudinal \| CES-D \| \| -Depression scores at baseline were correlated with negative affect (NA, *r* = .31 *p* < .05), level of rumination at baseline (*r* = .29 *p* < .01) and end of study (*r* = .39 *p* < .001). Depression scores at the end of the study were correlated with NA (*r* = .31 *p* < .01), positive affect (PA, *r* = -.29 *p* < .01), impact of distract on NA (*r* = -.26 *p* < .01), level of rumination at baseline (*r* = .33 *p* < .01) and end of study (*r* = .31 *p* < .01).  -Several models were used to predict depression severity over time: when rumination and the impact of rumination on NA were included, only rumination was a predictor of depression. When PA, rumination, impact of distraction on NA or PA were included, only rumination was a predictor of changes in depression severity over time. \| \| 85 \| 151 \| Pe et al., 2014 \| Emotion-Network Density in Major Depressive Disorder. \| USA \| 104 \| 18-40 / 26.8 \| 67% / 30% \| English speaker adults who experienced no current or past mental health disorders. \| 1 week \| Quantitative, longitudinal \| BDI-II \| \| - Compared to controls, depressed participants had an overall denser emotional network (*t* (102) = 10.08, *p* < .001), experienced more negative emotions (*t* (102) = 12.34, *p* < .001), more anxious thoughts (*t* (102) = 5.43, *p* < .001), sadness (*t* (102) = 10.92, *p* < .001), disgust (*t* (102) = 10.44, *p* < .001) anger (*t* (102) = 6.63, *p* < .001), guilt (*t* (102) = 9.64, *p* < .001), shame (*t* (102) = 9.23, *p* < .001), and frustration (*t* (102) = 6.07, *p* < .001).  -Women in the depressed group experienced a denser emotional network than men (*t* (51) = -2.24, *p* = .03), and this difference was not observed in the control group.  -Compared to controls, participants in the depressed group experienced increased variability in the intensity of their emotions (*t* (102) = 9.83, *p* < .01), and increased variability in their negative emotions (*t* (102) = 12.34, *p* < .01), but not in their positive emotions. \| \| 86 \| 152 \| Pedrelli et al., 2020 \| Monitoring Changes in Depression Severity Using Wearable and Mobile Sensors \| USA \| 31 \| 19-73 / 33.7 \| 71%/ 26% \| Adults diagnosed with Major Depressive Disorder. \| 8 weeks \| Quantitative, longitudinal \| HDRS,  MINI \| \| -Model including features from mobile phone (i.e., calls, texts, and activity patterns) and excluding wearable features (electrodermal activity, skin temperature, heart rate, accelerometer, and actigraphy) best predicted depression severity when conducting a time-split in data (*F* [2, 12] = 19.04, *p* < .002).  -There was no difference across models (i.e., mobile only, wearable only, mobile + wearable) when conducting a user-split in data. \| \| 87 \| 153 \| Peterson et al., 2020 \| Comparing integrative cognitive-affective therapy and guided self-help cognitive-behavioral therapy to treat binge-eating disorder using standard and naturalistic momentary outcome measures: A randomized controlled trial. \| USA \| 112 \| 18-65 / 39.7 \| 91% / 18% \| Adults with diagnosed binge eating disorder. \| 17 weeks / 6 months follow up \| Quantitative, longitudinal, randomized control trial \| BDI \| \| -There was no significant main effect on depression for treatment group (i.e., integrative cognitive-affective therapy for binge-eating disorder, ICAT-BED, and cognitive behavioural therapy guided self-help, CBT). \| \| 88 \| 154 \| Place et al., 2017 \| Behavioral Indicators on a Mobile Sensing Platform Predict Clinically Validated Psychiatric Symptoms of Mood and Anxiety Disorders \| USA \| 73 \| 18-70 / -- \| 48% / 67% \| Adults who reported at least one symptom of PTSD or Depression. \| 12 weeks \| Quantitative, longitudinal \| PHQ-2 \| \| -Model predicting depressed mood including audio features showed a .74 AUC accuracy.  -Model predicting diminished interest or pleasure in activities including SMS data and distance travelled (via GPS) showed a .56 AUC accuracy.  -Model predicting fatigue or loss of energy including call and SMS data showed a .75 AUC accuracy.  -Model predicting avoidance of activities, places and people including call, SMS and an interaction term between these features showed a .83 AUC accuracy. \| \| 89 \| 155 \| Putnam & McSweeney, 2007 \| Depressive symptoms and baseline prefrontal EEG alpha activity: A study utilizing Ecological Momentary Assessment. \| USA \| 13 \| --/ 32.7 \| 85% / 31% \| Depressed adults and healthy control group. \| 1 week \| Quantitative, longitudinal, non-randomized control trial \| SCID \| \| -Group (i.e., depressed vs controls) x EEG interactions significantly predicted self-reported rumination (*p* < .02) for specific brain sites (i.e., FP1, FP2, F3, F7, and C4).  -Similarly, group x EEG interactions significantly predicted self-reported self-esteem (*p* < .05) for F4 and C4.  -Group alone predicted rumination (*F* [1, 362] = 3.95, *p* < .05), and self-esteem (*F* [1, 362] = 80.8, *p* < .001), with the depressed group reporting higher levels of rumination and lower self-esteem. \| \| 90 \| 157 \| Robbins et al., 2011 \| Naturalistically Observed Sighing and Depression in Rheumatoid Arthritis Patients: A Preliminary Study. \| USA \| 13 \| --/ 56 \| 92% /N.A. \| Female patients with Rheumatoid arthritis. \| 4 non-consecutive days / 6 months follow up. \| Quantitative, longitudinal \| BDI, CES-D \| \| -Sighing frequency was significantly related to participant’s depression severity via BDI (*r* = .71, *p* = .01) and CES-D (*r* = .68, *p* = .01).  -Sighing was significantly more strongly related to depression than frequency of pain flare (BDI: *p* = .04; CES-D: *p* = .02). \| \| 91 \| 158 \| Rodriguez et al., 2021 \| Idiographic Network Models of Social Media Use and Depression Symptoms. \| Netherlands \| 125 \| --/ 20.4 \| --/ 30% \| Dutch undergraduate students. \| 2 weeks \| Quantitative, longitudinal \| -- \| \| -Higher inter-individual variability in associations between variables were observed for active media use and: passive media use, feeling inferior, feeling hopeless, depressed mood. Similarly, there was high inter-individual variability in associations between passive social media use and loss of interest.  -Passive and active social media use were ‘very weakly’ to ‘weakly’ associated with depression symptoms for most individuals. This applies to both directions (from depression to social media, and from social media to depression).  -Social media use (either active or passive) resulted in higher stress and depressive symptoms in 45% of participants.  -Stress and depressive symptoms resulted in higher social media use (active or passive) in 62% of participants. \| \| 92 \| 183 \| Roekel et al., 2016 \| Depressive Symptoms and the Experience of Pleasure in Daily Life: An Exploration of Associations in Early and Late Adolescence. \| Netherlands \| 284 / 74 \| -- /14.2 / 20.9 \| --/ 41% / N.A. \| Early and late adolescents. \| 6-14 days \| Quantitative, longitudinal \| SCL-90 \| \| -Depressive symptoms were associated with less positive affect (PA; *B* = -.22, *p* < .001), higher levels of variability in PA (*B* = .04, *p* < .01), higher levels of instability (*B* = .05, *p* < .01), less positive events (*B* = -.04, *p* < .01) and less pleasure during positive events (*B* = -.09, *p* < .01).  -Anhedonia was also associated with less positive events (*B* = -.02, *p* < .05) but not level of pleasure during events, PA, variability in PA, or instability in PA.  -No differences were observed for the PA-depression relationship across age groups and gender. \| \| 93 \| 160 \| Sagar et al., 2016 \| Joint Effects: A Pilot Investigation of the Impact of Bipolar Disorder and Marijuana Use on Cognitive Function and Mood. \| USA \| 74 \| -- / 24.2 \| --/ 58% \| Patients with bipolar disorder who smoke marijuana. \| 4 weeks \| Quantitative, longitudinal \| MADRS \| \| -Significantly different levels of depression were observed across groups, with healthy controls (HC) and marijuana users (MJ) showing lower levels compared to participants diagnosed with bipolar (BP) that did (BPMJ) or did not smoke marihuana (HC vs BP, *p* < .001; HC vs MJBP, *p* < .001).  -Significant differences in depression scores across groups were still observed after controlling for age and pre/post marijuana use (HC vs BP, *p* < .001; HC vs MJBP, *p* < .001).  -Participants in the BPMJ group showed significant increment in depression severity after smoking marijuana (*p* < .05). This effect was not observed in the MJ group. \| \| 94 \| 162 \| Schultebraucks et al., 2020 \| Deep learning-based classification of posttraumatic stress disorder and depression following trauma utilizing visual and auditory markers of arousal and mood. \| USA \| 81 \| 18-70 / 37.9 \| 75% / 57% \| Trauma survivors admitted to the emergency department of a trauma centre. \| -- \| Quantitative, cross-sectional \| CES-D \| \| -Neural networks using facial features, speech content, voice prosody and movement features achieved good predictive power for symptoms of depression (AUC = .86, *R*^2^ = .62).  -The most important predictors of depression status (i.e., depressed vs non-depressed) were age, natural language processing features (such as ‘workhorse’, ‘organised’ and ‘friends focused’), voice prosody (such as audio intensity), facial features of emotion, and movement features (such as pupil dilation). \| \| 95 \| 163 \| Sears et al., 2018 \| Emotional and social reactivity as mechanisms of stress generation: a momentary assessment study. \| USA \| 110 \| --/ 20.5 \| 30% / N.A. \| Female undergraduate students. \| 5 days \| Quantitative, longitudinal \| CESD-D \| \| -Emotional distress was bidirectionally influenced by interpersonal stressors such as conflict, rejection, or criticism.  -Avoidant responses to stressors significantly contributed to negative affect and mood. Specifically, depressed individuals showed tendency to respond to negative moods with avoidance behaviour. \| \| 96 \| 166 \| Sheets & Armey, 2020 \| Daily Interpersonal and Non-interpersonal Stress reactivity in Current and Remitted Depression. \| USA \| 104 \| 18-22 / 18.5 \| 60% / 36% \| Adults with current and remitted depression. \| 2 weeks \| Quantitative, longitudinal, non-randomized control trial \| BDI-II, SCID \| \| -Participants with current depression reported significantly greater negative affect (NA) compared to those with remitted depression (*t* = 2.82, *p* = .006) and those with no history of depression (*t* = 4.48, *p* < .001). Participants with no history of depression reported less stress than those with current depression (*t* = 3.34, *p* = .001) and those with remitted depression (*t* = 2.10, *p* = .038).  -Currently depressed participants rated events as significantly more stressful (*t* = 2.01, *p* = .048), less sense of control over stressful events (*t* = -2.02, *p* = .046), and poorer coping with negative events (*t* = -2.99, *p* = .004) compared to those without a history of depression.  -All groups (i.e. current, remitted and no history of depression) reported significant stress reactivity (*p* < .001) such that increased stress predicted increased NA. However, depression status moderated stress reactivity as the effect of stress on NA was greater in participants with current and remitted depression compared to non-depressed. \| \| 97 \| 167 \| Snippe et al., 2016 \| Change in Daily Life Behaviors and Depression: Within-Person and Between-Person Associations. \| Netherlands \| 102 \| 18-65 / 48 \| --/ 46% \| Adults with a diagnosis of depression who received antidepressants or mood stabilisers. \| 6 weeks \| Quantitative, longitudinal, randomized control trial \| HDRS, SCL-90-R \| \| -On average, participants reported lower depressive symptoms at the end of the day when they had engaged in physical activity (*B =* .46, *p* < .01), were talking (*B =* .46, *p* < .01), in company (*B =* .44, *p* < .01), engaged in leisure activities (*B =* .44, *p* < .01), and less engaged in resting or doing nothing (*B =* .48, *p* < .01).  -Both experimental and pseudo-experimental groups showed significant post intervention improvement compared to controls in frequency of resting/doing nothing (*Z* = -3.16, *p* < .01). However, only the pseudo-experimental group showed significant improvement in talking (*Z* = 3.73, *p* < .01), and being alone (*Z* = -2.56, *p* = .01). \| \| 98 \| 168 \| Sperry et al., 2018 \| Psychopathology, everyday behaviors, and autonomic activity in daily life: An ambulatory impedance cardiography study of depression, anxiety, and hypomanic traits. \| USA \| 49 \| 18-25 / 19.3 \| 37% / 22% \| University students. \| 1 day \| Quantitative, longitudinal \| DASS-21 \| \| -Depression severity was not a significant predictor of posture (sitting/standing), eating, drinking, physical activity, or social activity.  -Depression severity was not a significant predictor of psychophysiological outcomes (time between ECG R peak and dZ peak, cardiac pre-ejection period, heart rate, respiration rate, and its variability measured by RMSSD).  -Posture, eating, and social activity mediated the relationship between depression and respiration rate. As depression scores increased, people were more likely to be alone (β = -.23, *p* = .001). In turn, as people interacted with others, their respiration rate decreased (β = -.23, *p* = .001). \| \| 99 \| 170 \| Stasak et al., 2019 \| Automatic depression classification based on affective read sentences: Opportunities for text-dependent analysis. \| Australia \| 70 \| 21-75 / 40 \| --/ 54% \| Depressed and healthy adults. \| -- \| Quantitative, cross-sectional \| QIDS \| \| - 65% Accuracy of speaker classification (depressed vs non-depressed, D and ND)  -Depressed speakers had sig greater number of ‘unvoiced’ speech frames. Similar number  of ‘voiced’ speech frames for D vs ND. Sig number of hesitations for D  -D speakers made 4 times the number of errors than non-D  -First person narratives produced better depression classification \| \| 100 \| 171 \| Steenkamp et al., 2019 \| The relationship between childhood abuse and severity of psychosis is mediated by loneliness: an experience sampling study. \| Netherlands \| 59 \| 19-57 / 31.8 \| --/ 58% \| Adults with non-affective psychotic disorder. \| 5 days \| Quantitative, longitudinal \| -- \| \| -Higher levels of loneliness were associated with higher depressive feelings at the within (β = .30, *p* < .001) and between subjects’ level (β = .81, *p* < .001). In turn more depressive feelings were associated with more positive feelings of psychosis because of childhood abuse at the within subjects’ level (β = .13, *p* < .001) and not at the between subjects’ level. This suggests that loneliness affects positive symptoms of psychosis through depression at the individual level. \| \| 101 \| 172 \| Thompson et al., 2015 \| Emotional Clarity as a Function of Neuroticism and Major Depressive Disorder. \| Belgium / USA \| 95 /106 \| 18-67 / 24 / 18-40/ 28.6 \| --/ 37%  68%/ 30% \| College students, and adults with diagnosis of major depressive disorder. \| 2 weeks \| Quantitative, longitudinal \| BDI-II, SCID \| \| -Compared to controls, the depressed group took longer in rating items eliciting negative emotions (*γ* = .136, *p* < .001) but not in items eliciting positive emotions. \| \| 102 \| 173 \| Thompson et al., 2016 \| The grass is not as green as you think: Affect evaluation in people with internalizing disorders. \| USA \| 70 \| 18-50 / 32.9 \| 61% / N.A. \| Adult women diagnosed with major depressive disorder. \| 1 week \| Quantitative, longitudinal \| BDI-II \| \| -All groups (depressed, MDD; anxious, GAD; depressed and anxious, MDD-GAD; and controls) reported higher than zero should positive affect (PA), suggesting that all groups should be feeling more PA. Clinical groups (MDD, GAD and MDD-GAD) reported significantly higher should PA than controls (*t*s (66) > 2.76, *p*s < .01), with no differences between clinical groups.  -Compared to controls, should NA was marginally lower in the MDD group (*t* (66) = 1.86, *p* = .06) and significantly lower in GAD and MDD-GAD (*t*s (66) > 3.54, *p*s < .001), with no differences across clinical groups.  -Mean NA was significantly lower in clinical groups compared to controls (*t*s (66) > 3.98, *p*s < .001). \| \| 103 \| 174 \| Thompson et al., 2017 \| Positive and Negative Affective Forecasting in Remitted Individuals with Bipolar I Disorder, and Major Depressive Disorder, and Healthy Controls. \| USA \| 84 \| 18-60 / 31 \| 89% / 38% \| Adults diagnosed with bipolar or major depressive disorder. \| 6 week \| Quantitative, longitudinal \| IDS-C \| \| -Depressive symptoms were not related to forecasted levels of positive (PA) or negative (NA) affect for either short term (how would you feel tomorrow?) or long-term affect (how would you feel next week?).  -Depressed participants reported significantly less short term and long term forecasted PA (*p* = .01) and more NA (*p* = .01) than controls, but similar ratings of PA and NA compared to participants diagnosed with bipolar disorder (BD).  -The relationship between short term and long term forecasted affect did not differ across groups. \| \| 104 \| 181 \| Trull et al., 2008 \| Affective Instability: Measuring a Core Feature of Borderline Personality Disorder with Ecological Momentary Assessment. \| USA \| 60 \| -- /35 \| 87% / 12% \| Outpatients with BPD and/or depressive disorder \| 1 month \| Quantitative, longitudinal \| SCID \| \| -Participants with BPD and MDD/DYS (dysthymia) did not significantly differed in their self-reported ratings of positive affect (PA), negative affect (NA), hostility, fear, or sadness.  -The model allowing varying variances across groups had a significant better fit for the BPD group, suggesting that participants with BPD displayed more variable affect compared to those with MDD/DYS.  -BPD group reported more PA and NA than MDD/DYS considering all time factors (i.e. linear and quadratic trends for changes across days, within the day, and interaction terms with weekends). However, differences across groups in hostility, fear or sadness were not significant. Moreover, BPD group reported significantly larger residuals for PA considering all time factors, suggesting that BPD individuals experience more variability in PA than MDD/DYS beyond observed time factors.  -Adjusting the data (with log link and binomial error distribution) to observe acute changes in mood resulted in significant variability for hostility scores in BPD group compared with MDD/DYS. \| \| 105 \| 184 \| Vansteelandt et al., 2019 \| Self-criticism and dependency predict affective variability in borderline personality disorder: An ecological momentary assessment study. \| Belgium \| 32 \| --/ 28 \| --/ 16% \| Patients with BPD \| 8 days \| Quantitative, longitudinal \| SCID \| \| -Higher self-criticism and dependency had a significant effect on depression mean scores (*t* = 3.59, *p* = .001; and *t* = 2.69, *p* < .01 respectively), and only self-criticism had a significant effect on within-subject variance for depression (*p* < .001) suggesting that those with higher self-criticism and dependence are more likely to experience depression. \| \| 106 \| 186 \| Verkuil et al., 2015 \| Gender differences in the impact of daily sadness on 24-h heart rate variability. \| USA \| 60 \| --/ 44.63 \| 78% / 32% \| Government workers \| 24 hours \| Quantitative, longitudinal \| CES-D \| \| -Female participants showed significantly higher depressive symptoms compared to male participants (*p* < .05).  -Depressive symptom (assessed via CES-D) were positively correlated with self-reported sadness (via EMAs; *r* = .43, *p* = .001).  -Controlling for time of the day, smoking, and physical fitness, the interaction effect between gender and momentary sadness in predicting depression symptoms was not significant. \| \| 107 \| 187 \| Vesel et al., 2020 \| Effects of mood and aging on keystroke dynamics metadata and their diurnal patters in large open-science sample: A BiAffect iOS study. \| USA \| 147 \| --/ 39.53 \| --/ 23% \| BiAffect users who reported demographic information. \| 15 months \| Quantitative, longitudinal \| PHQ-8 \| \| -Participants with severe depression (scores >20 in PHQ-8) corresponded to a 2.2% shortening of the 50^th^ percentile in inter-key delay (IKD) and 7.8% increase in pausing compared to participants with no depression. This suggests higher IKD in depressed participant.  -Participants with severe depression (scores >20 in PHQ-8) showed 7.2% increase in typing mistakes, and 7.95% shorter sessions compared to participants with no depression. \| \| 108 \| 188 \| Vranceanu et al., 2009 \| Depressive symptoms and momentary affect: the role of social interaction variables. \| USA \| 108 \| --/ 41.07 \| 86% / N.A. \| Middle-aged female staff member of a university. \| 2 days \| Quantitative, longitudinal \| CES-D \| \| -Depression symptoms were positively related to negative affect (*t* = 3.78, *p* < .01), social conflict (*t* = 4.69, *p* < .01), and negatively related to positive affect (*t* = -3.11, *p* < .05), but did not significantly predict variability in social support.  - Depression symptoms were positively related to negative affect (*t* = 2.64, *p* < .01), and negatively related to positive affect (*t* = -1.53, *p* < .01) after controlling for social conflict. \| \| 109 \| 189 \| Wahle et al., 2016 \| Mobile Sensing and Support for People with Depression: A Pilot Trial in the Wild. \| Switzerland \| 126 \| 20-57 / -- \| --/-- \| Adults. \| 2 weeks \| Quantitative, longitudinal, non-randomized control trial \| PHQ-9 \| \| -Subjects were divided in intervention (using a mobile sensing and support app providing in-time interventions) and control. Those who showed symptoms of clinical depression at baseline and used the intervention app for at least 8 weeks showed a significant drop in depression symptoms (*p* = .01).  -There was a negative but not significant correlation between adherence to intervention app and change in depression scores (ρ = -.498, *p* = .099). \| \| 110 \| 190 \| Wang et al., 2021 \| Variability in emotion regulation strategy use is negatively associated with depressive symptoms. \| China \| 213 \| 18-26 / 19.8 \| --/ 45% \| Undergraduate university students \| 10 days \| Quantitative, longitudinal \| BDI-II \| \| -Emotion regulation strategies (*B* = .144, *p* = .018), between-strategy SD (*B* = -.220, *p* = .025), and NA inertia (*B* = 6.12, *p* = .006) were significant predictors of depression severity. \| \| 111 \| 191 \| Wenze et al., 2006 \| Influence of dysphoria on positive and negative cognitive reactivity to daily mood fluctuations. \| USA \| 102 \| --/-- \| 80% / 23% \| Undergraduate university students \| 1 week \| Quantitative, longitudinal \| CES-D \| \| -Participants with higher initial dysphoria reported more negative thoughts (*b* = .934, *p* < .001), negative affect (*b* = .695, *p* < .001), less positive thoughts (*b* = -.591, *p* = .01), and less positive affect (*b* = -.513, *p* = .008) during the study.  -Higher levels of negative thoughts were associated with higher levels of negative affect, and higher levels of positive thoughts were associated with positive affect. Dysphoria moderated these relationship such that the negative thoughts-affect associations were stronger and positive thoughts-affect associations lesser in participants with higher levels of dysphoria.  -Participants with higher negative cognitions tend to increase following an increase in negative affect. This relationship was stronger as levels of dysphoria increased. \| \| 112 \| 192 \| Wenze et al., 2009 \| The Influence of Dysphoria on Reactivity to Naturalistic Fluctuations in Anger. \| USA \| 102 \| --/-- \| 80% / 77% \| Psychology university students \| 1 week \| Quantitative, longitudinal \| CES-D \| \| -Participants with higher initial dysphoria reported more momentary anger (*b* = 032, *p* < .01), depressed mood (*b* = .054, *p* < .001), rumination (*b* = .060, *p* < .001), dependency (*b* = .060, *p* < .001), and negative interpersonal events (*b* = 008, *p* < .05) compared to those with lower dysphoria. Negative interpersonal events did not vary as a function of dysphoria.  -Dysphoria moderated the relationship between anger and depressed mood, and the relationship between anger and subsequent mood, such that in individuals with higher dysphoria this association was stronger.  -Dysphoria did not moderate the relationship between anxiety and depressed mood, or between guilt and depressed mood.  -Rumination and dependency mediated the relationship between anger and depressed mood (*Z* = 5.2, *p* < .001; *Z* = 4.27, *p* < .001). While controlling for rumination, the path between anger and depressed mood was stronger for participants with high dysphoria. Similarly, participants with high dysphoria experiencing a rise in dependency reported an even stronger increase in depressed mood. \| \| 113 \| 193 \| Wenze et al., 2012 \| Biases in Affective Forecasting and Recall in Individuals with Depression and Anxiety Symptoms. \| USA \| 120 \| 18-25 / 19.7 \| 75% / 32% \| Psychology university students \| 1 week \| Quantitative, longitudinal \| MASQ \| \| -Higher depression levels were associated with more negative mood (β = .36, *p* < .001), less positive mood (β = -.76, *p* < .001), more negative mood recall (β = .36, *p* < .001), less positive mood recall (β = -.57 *p* < .001)  -Depressive symptoms predicted bias in the prediction of depressed mood (β = .38, *p* < .001) and recall depressed mood (β = .18, *p* = .01) but not in the prediction of anxious nor recall anxious mood.  -Participants with high depressive symptoms expected the occurrence of more negative events (β = .22, *p* = .02) and less positive events (β = -.38, *p* < .001). Similarly, participants with higher depressive symptoms experienced less positive events (β = -.37, *p* < .001) but not more negative events (β = .07, *p* = .49). \| \| 114 \| 194 \| Wenze et al., 2018 \| Momentary experiential avoidance: Within-person correlates, antecedents, and consequences and between-person moderators. \| USA \| 104 \| --/ 19.15 \| 64% / 22% \| Undergraduate university students \| 1 week \| Quantitative, longitudinal \| CES-D \| \| -Depression severity was positively correlated with negative mood (*r* = .59, *p* < .001), negative thoughts (*r* = .63, *p* < .001), experiential avoidance (EA; *r* = .63, *p* < .001), perceived stress (*r* = .41, *p* < .001), and negatively correlated with (*r* = -.45, *p* < .001) positive mood, and positive thoughts (*r* = -.50, *p* < .001).  -Depressed participants were more likely to report EA when they were experiencing higher negative mood, negative thoughts, and stress. Conversely depressed participants were less likely to report EA when experiencing higher positive mood and thoughts. Similar effects were observed on lagged self-reports (i.e., from one EMA to the next one).  -Classification models including GPS, accelerometer, and communication logs predicting group classification (depressed vs non-depressed) showed 60.1% accuracy with a random forest algorithm and 59.1% with support vector machine. \| \| 115 \| 196 \| Worten-Chaudhari et al., 2017 \| Reducing concussion symptoms among teenage youth: Evaluation of a mobile health app. \| USA \| 20+19 \| 13-18 / 15.6 \| --/ 28% \| Adolescents with unresolved concussion symptoms. \| 8 weeks \| Quantitative, longitudinal, non-randomized control trial \| CES-D \| \| -Baseline depression scores across groups were not significantly different (experimental using an app designed to ameliorate unresolved post-concussion symptoms, and control group).  -Change in depression scores after intervention was not significantly different across groups (*p* = .156). This suggests that post-concussion secondary symptoms such as depression are not well targeted by the app. \| \| 116 \| 197 \| Wu et al., 2016 \| Anticipatory and Consummatory Pleasure and Displeasure in Major Depressive Disorder: An Experience Sampling Study. \| USA \| 80 \| 18-55 / 33.6 \| 62% / 20% \| English speakers who could undergo fMRI. \| 1 week \| Quantitative, longitudinal \| SCID-I \| \| -Depressed participants reported lower levels of anticipatory (*γ* = -7.30, *t* (78) = -3.09, *p* =.003) and consummatory pleasure (*γ* = -7.82, *t* (73) = -3.02, *p* =.004), and higher levels of anticipatory (*γ* = -9.18, *t* (78) = -3.67, *p* <.001) and consummatory (*γ* = -8.41, *t* (78) = -3.25, *p* =.01) displeasure compared to controls.  -Both groups (depressed and non-depressed) showed similar decrease in anticipatory pleasure as the day progressed (*γ* = .008, *t* (2121) = 3.30, *p* =.001). After controlling for time-of-day effects, previously significant differences between groups remained.  -Depressed participants reported greater overestimation of anticipatory pleasure (*γ* = 3.41, *t* (73) = 2.17, *p* =.03) and not displeasure compared to controls. \| \| 117 \| 198 \| Zhang et al., 2019 \| Automated voice biomarkers for depression symptoms using an online cross-sectional data collection initiative. \| USA \| 222 \| 11-65+ / -- \| 34% / 19% \| Users of the Mental Health America website. \| -- \| Quantitative, cross-sectional \| PHQ-9 \| \| -Acoustic, prosodic, and linguistic features similarly and significantly predicted depression severity (*p* < .05). Prediction indices were better for the free-speech task compared to the self-introduction task. \| \| 118 \| 200 \| Zulueta et al., 2018 \| Predicting Mood Disturbance Severity with Mobile Phone Keystroke Metadata: A BiAffect Digital Phenotyping Study \| USA \| 9 \| --/ 48.7 \| --/ 11% \| Adults with diagnosis of Bipolar \| 8 weeks \| Quantitative, longitudinal \| HDRS \| \| -The full model showed superior fit (*χ*^2^ = 17.6, *p* = .01) indicating that all typing features contributed to predicting depression severity.  -Accelerometer displacement (*p* = .002), average inter-key delay (*p* = .02), session count (i.e. how many phone sessions; *p* = .003) and autocorrect rate (considered a measure of cognitive performance; *p* = .002) were positively correlated with depression severity.  -Diurnal activity and circadian baseline similarity (obtained through hourly distribution of keypresses/week and the hourly distribution) not correlated with depression severity. \| \|  \| \| *Note: BDI-II = Beck Depression Inventory-II (Beck et al., 1996); CDI = Children’s Depression Inventory (Kovacs, 1981); CES-D = 20-item Center for Epidemiological Studies Depression Questionnaire (Radloff, 1977); DAMS = Depression and Anxiety Measurement Scale (Fukui, 1997); DASS-21 = Depression, Anxiety and Stress Scale 21-items (Lovibond & Lovibond, 1995); DESC = Rasch-based Depression Screening (Forkmann et al., 2009); DID = Diagnostic Inventory for Depression (Zimmerman et al., 2003);EPDS = Edinburgh Postnatal Depression Scale (Cox et al., 1987); HDRS/ HAM-D = Hamilton Depression Rating Scale (Hamilton, 1967); IDS = Inventory of Depressive Symptomatology (Rush et al., 2009); MADRS = Montgomery and Asberg Depression Scale (Montgomery & Asberg, 1979); MASQ = Mood and Anxiety Symptom Questionnaire (Clark & Watson, 1991); MINI 5.0 = Mini-International Neuropsychiatric Interview (Sheehan et al., 1998); PDQ-D = Perceived Deficits Questionnaire – Depression; PHQ-8/9 = Patient Health Questionnaire 8-item (Kroenke et al., 2001); POMS = Profile of Mood States (McNair et al., 1981); al., 1989); QIDS-C/QIDS-SR = Quick Inventory of Depressive Symptomatology clinician rating (Rush et al., 2003); RAND-36 = 36-item Short Form Survey (Hays & Morales, 2001); SCID = Structured Clinical Interview for DSM-IV-TR (First et al., 2002); SCL = Symptom Checklist (Derogatis, 1977)* \| \| \| \| \| \| \| \| \| \| \| \| |
| --- | --- | --- | --- | --- | --- | --- | --- | --- | --- | --- | --- | --- | --- | --- | --- | --- | --- | --- | --- | --- | --- | --- | --- | --- | --- | --- | --- | --- | --- | --- | --- | --- | --- | --- | --- | --- | --- | --- | --- | --- | --- | --- | --- | --- | --- | --- | --- | --- | --- | --- | --- | --- | --- | --- | --- | --- | --- | --- | --- | --- | --- | --- | --- | --- | --- | --- | --- | --- | --- | --- | --- | --- | --- | --- | --- | --- | --- | --- | --- | --- | --- | --- | --- | --- | --- | --- | --- | --- | --- | --- | --- | --- | --- | --- | --- | --- | --- | --- | --- | --- | --- | --- | --- | --- | --- | --- | --- | --- | --- | --- | --- | --- | --- | --- | --- | --- | --- | --- | --- | --- | --- | --- | --- | --- | --- | --- | --- | --- | --- | --- | --- | --- | --- | --- | --- | --- | --- | --- | --- | --- | --- | --- | --- | --- | --- | --- | --- | --- | --- | --- | --- | --- | --- | --- | --- | --- | --- | --- | --- | --- | --- | --- | --- | --- | --- | --- | --- | --- | --- | --- | --- | --- | --- | --- | --- | --- | --- | --- | --- | --- | --- | --- | --- | --- | --- | --- | --- | --- | --- | --- | --- | --- | --- | --- | --- | --- | --- | --- | --- | --- | --- | --- | --- | --- | --- | --- | --- | --- | --- | --- | --- | --- | --- | --- | --- | --- | --- | --- | --- | --- | --- | --- | --- | --- | --- | --- | --- | --- | --- | --- | --- | --- | --- | --- | --- | --- | --- | --- | --- | --- | --- | --- | --- | --- | --- | --- | --- | --- | --- | --- | --- | --- | --- | --- | --- | --- | --- | --- | --- | --- | --- | --- | --- | --- | --- | --- | --- | --- | --- | --- | --- | --- | --- | --- | --- | --- | --- | --- | --- | --- | --- | --- | --- | --- | --- | --- | --- | --- | --- | --- | --- | --- | --- | --- | --- | --- | --- | --- | --- | --- | --- | --- | --- | --- | --- | --- | --- | --- | --- | --- | --- | --- | --- | --- | --- | --- | --- | --- | --- | --- | --- | --- | --- | --- | --- | --- | --- | --- | --- | --- | --- | --- | --- | --- | --- | --- | --- | --- | --- | --- | --- | --- | --- | --- | --- | --- | --- | --- | --- | --- | --- | --- | --- | --- | --- | --- | --- | --- | --- | --- | --- | --- | --- | --- | --- | --- | --- | --- | --- | --- | --- | --- | --- | --- | --- | --- | --- | --- | --- | --- | --- | --- | --- | --- | --- | --- | --- | --- | --- | --- | --- | --- | --- | --- | --- | --- | --- | --- | --- | --- | --- | --- | --- | --- | --- | --- | --- | --- | --- | --- | --- | --- | --- | --- | --- | --- | --- | --- | --- | --- | --- | --- | --- | --- | --- | --- | --- | --- | --- | --- | --- | --- | --- | --- | --- | --- | --- | --- | --- | --- | --- | --- | --- | --- | --- | --- | --- | --- | --- | --- | --- | --- | --- | --- | --- | --- | --- | --- | --- | --- | --- | --- | --- | --- | --- | --- | --- | --- | --- | --- | --- | --- | --- | --- | --- | --- | --- | --- | --- | --- | --- | --- | --- | --- | --- | --- | --- | --- | --- | --- | --- | --- | --- | --- | --- | --- | --- | --- | --- | --- | --- | --- | --- | --- | --- | --- | --- | --- | --- | --- | --- | --- | --- | --- | --- | --- | --- | --- | --- | --- | --- | --- | --- | --- | --- | --- | --- | --- | --- | --- | --- | --- | --- | --- | --- | --- | --- | --- | --- | --- | --- | --- | --- | --- | --- | --- | --- | --- | --- | --- | --- | --- | --- | --- | --- | --- | --- | --- | --- | --- | --- | --- | --- | --- | --- | --- | --- | --- | --- | --- | --- | --- | --- | --- | --- | --- | --- | --- | --- | --- | --- | --- | --- | --- | --- | --- | --- | --- | --- | --- | --- | --- | --- | --- | --- | --- | --- | --- | --- | --- | --- | --- | --- | --- | --- | --- | --- | --- | --- | --- | --- | --- | --- | --- | --- | --- | --- | --- | --- | --- | --- | --- | --- | --- | --- | --- | --- | --- | --- | --- | --- | --- | --- | --- | --- | --- | --- | --- | --- | --- | --- | --- | --- | --- | --- | --- | --- | --- | --- | --- | --- | --- | --- | --- | --- | --- | --- | --- | --- | --- | --- | --- | --- | --- | --- | --- | --- | --- | --- | --- | --- | --- | --- | --- | --- | --- | --- | --- | --- | --- | --- | --- | --- | --- | --- | --- | --- | --- | --- | --- | --- | --- | --- | --- | --- | --- | --- | --- | --- | --- | --- | --- | --- | --- | --- | --- | --- | --- | --- | --- | --- | --- | --- | --- | --- | --- | --- | --- | --- | --- | --- | --- | --- | --- | --- | --- | --- | --- | --- | --- | --- | --- | --- | --- | --- | --- | --- | --- | --- | --- | --- | --- | --- | --- | --- | --- | --- | --- | --- | --- | --- | --- | --- | --- | --- | --- | --- | --- | --- | --- | --- | --- | --- | --- | --- | --- | --- | --- | --- | --- | --- | --- | --- | --- | --- | --- | --- | --- | --- | --- | --- | --- | --- | --- | --- | --- | --- | --- | --- | --- | --- | --- | --- | --- | --- | --- | --- | --- | --- | --- | --- | --- | --- | --- | --- | --- | --- | --- | --- | --- | --- | --- | --- | --- | --- | --- | --- | --- | --- | --- | --- | --- | --- | --- | --- | --- | --- | --- | --- | --- | --- | --- | --- | --- | --- | --- | --- | --- | --- | --- | --- | --- | --- | --- | --- | --- | --- | --- | --- | --- | --- | --- | --- | --- | --- | --- | --- | --- | --- | --- | --- | --- | --- | --- | --- | --- | --- | --- | --- | --- | --- | --- | --- | --- | --- | --- | --- | --- | --- | --- | --- | --- | --- | --- | --- | --- | --- | --- | --- | --- | --- | --- | --- | --- | --- | --- | --- | --- | --- | --- | --- | --- | --- | --- | --- | --- | --- | --- | --- | --- | --- | --- | --- | --- | --- | --- | --- | --- | --- | --- | --- | --- | --- | --- | --- | --- | --- | --- | --- | --- | --- | --- | --- | --- | --- | --- | --- | --- | --- | --- | --- | --- | --- | --- | --- | --- | --- | --- | --- | --- | --- | --- | --- | --- | --- | --- | --- | --- | --- | --- | --- | --- | --- | --- | --- | --- | --- | --- | --- | --- | --- | --- | --- | --- | --- | --- | --- | --- | --- | --- | --- | --- | --- | --- | --- | --- | --- | --- | --- | --- | --- | --- | --- | --- | --- | --- | --- | --- | --- | --- | --- | --- | --- | --- | --- | --- | --- | --- | --- | --- | --- | --- | --- | --- | --- | --- | --- | --- | --- | --- | --- | --- | --- | --- | --- | --- | --- | --- | --- | --- | --- | --- | --- | --- | --- | --- | --- | --- | --- | --- | --- | --- | --- | --- | --- | --- | --- | --- | --- | --- | --- | --- | --- | --- | --- | --- | --- | --- | --- | --- | --- | --- | --- | --- | --- | --- | --- | --- | --- | --- | --- | --- | --- | --- | --- | --- | --- | --- | --- | --- | --- | --- | --- | --- | --- | --- | --- | --- | --- | --- | --- | --- | --- | --- | --- | --- | --- | --- | --- | --- | --- | --- | --- | --- | --- | --- | --- | --- | --- | --- | --- | --- | --- | --- | --- | --- | --- | --- | --- | --- | --- | --- | --- | --- | --- | --- | --- | --- | --- | --- | --- | --- | --- | --- | --- | --- | --- | --- | --- | --- | --- | --- | --- | --- | --- | --- | --- | --- | --- | --- | --- | --- | --- | --- | --- | --- | --- | --- | --- | --- | --- | --- | --- | --- | --- | --- | --- | --- | --- | --- | --- | --- | --- | --- | --- | --- | --- | --- | --- | --- | --- | --- | --- | --- | --- | --- | --- | --- | --- | --- | --- | --- | --- | --- | --- | --- | --- | --- | --- | --- | --- | --- | --- | --- | --- | --- | --- | --- | --- | --- | --- | --- | --- | --- | --- | --- | --- | --- | --- | --- | --- | --- | --- | --- | --- | --- | --- | --- | --- | --- | --- | --- | --- | --- | --- | --- | --- | --- | --- | --- | --- | --- | --- | --- | --- | --- | --- | --- | --- | --- | --- | --- | --- | --- | --- | --- | --- | --- | --- | --- | --- | --- | --- | --- | --- | --- | --- | --- | --- | --- | --- | --- | --- | --- | --- | --- | --- | --- | --- | --- | --- | --- | --- | --- | --- | --- | --- | --- | --- | --- | --- | --- | --- | --- | --- | --- | --- | --- | --- | --- | --- | --- | --- | --- | --- | --- | --- | --- | --- | --- | --- | --- | --- | --- | --- | --- | --- | --- | --- | --- | --- | --- | --- | --- | --- | --- | --- | --- | --- | --- | --- | --- | --- | --- | --- | --- | --- | --- | --- | --- | --- | --- | --- | --- | --- | --- | --- | --- | --- | --- | --- | --- | --- | --- | --- | --- | --- | --- | --- | --- | --- | --- | --- | --- | --- | --- | --- | --- | --- | --- | --- | --- | --- | --- | --- | --- | --- | --- | --- | --- | --- | --- | --- | --- | --- | --- | --- | --- | --- | --- | --- | --- | --- | --- | --- | --- | --- | --- | --- | --- | --- | --- | --- | --- | --- | --- | --- | --- | --- | --- | --- | --- | --- | --- | --- | --- | --- | --- | --- | --- | --- | --- | --- | --- | --- | --- | --- | --- | --- | --- | --- | --- | --- | --- | --- | --- | --- | --- | --- | --- | --- | --- | --- | --- | --- | --- | --- | --- | --- | --- | --- | --- | --- | --- | --- | --- | --- | --- | --- | --- | --- | --- | --- | --- | --- | --- | --- | --- | --- | --- | --- | --- | --- | --- | --- | --- | --- | --- | --- | --- | --- | --- | --- | --- | --- | --- | --- | --- | --- | --- | --- | --- | --- | --- | --- | --- | --- | --- | --- | --- | --- | --- | --- | --- | --- | --- | --- | --- | --- | --- | --- | --- | --- | --- | --- | --- | --- | --- | --- | --- | --- | --- | --- | --- | --- | --- | --- | --- | --- | --- | --- | --- | --- | --- | --- | --- | --- | --- | --- | --- | --- | --- | --- | --- | --- | --- | --- | --- | --- | --- | --- | --- | --- | --- | --- | --- | --- | --- | --- | --- | --- | --- | --- | --- | --- | --- | --- | --- | --- | --- | --- | --- | --- | --- | --- | --- | --- | --- | --- | --- | --- | --- | --- | --- | --- | --- | --- | --- | --- | --- | --- | --- | --- | --- | --- | --- | --- | --- | --- | --- | --- | --- | --- | --- | --- | --- | --- | --- | --- | --- | --- | --- | --- | --- | --- | --- | --- | --- | --- | --- | --- | --- | --- | --- | --- | --- | --- | --- | --- | --- | --- | --- | --- | --- | --- | --- | --- | --- | --- | --- | --- | --- | --- | --- | --- | --- | --- | --- | --- | --- | --- | --- | --- | --- | --- | --- | --- | --- | --- | --- | --- | --- | --- | --- | --- | --- | --- | --- | --- | --- | --- | --- | --- | --- | --- | --- | --- | --- | --- | --- | --- | --- | --- | --- | --- | --- | --- | --- | --- | --- | --- | --- |

Supplementary Table 2. *Risk of bias assessment*

| Study Number | Study Number as listed in the references |  | Sample randomization / self-selected sampling^1^ | Participant eligibility criteria^2^ | Confounding factor identified^3^ | Measurement bias^4^ | Adequate participant description^5^ | Follow up longer than one year^6^ | Participant attrition^7^ | Symptom severity^8^ | Bias in analysis^9^ | Total | Comments |
| --- | --- | --- | --- | --- | --- | --- | --- | --- | --- | --- | --- | --- | --- |
| 1 | 1 | Abela et al., 2007 | ● | ○ | ● | ○ | ○ | ● | ● | ○ | ○ | 4 | -No potential confounding factor identified (PCFI). |
| 2 | 2 | Adams et al., 2009 | ● | ○ | ● | ○ | ○ | ● | ● | ○ | ○ | 4 | -No PCFI. |
| 3 | 6 | Bai et al., 2021 | ● | ○ | ○ | ○ | ● | ● | ● | ○ | ○ | 4 | -PCFI = Several passive features. |
| 4 | 8 | Bartels et al., 2020 | ○ | ○ | ● | ○ | ○ | ● | ○ | ○ | ○ | 2 | -No PCFI. |
| 5 | 12 | Ben-Zeev et al., 2009 | ● | ○ | ● | ○ | ○ | ● | ○ | ○ | ○ | 3 | -No PCFI. |
| 6 | 13 | Ben-Zeev et al., 2015 | ○ | ● | ● | ○ | ○ | ● | ● | ○ | ○ | 4 | -Minimal bias detected in sample selection process. No selection criteria outlined, and no confounding effects identified. No description of participant attrition. |
| 7 | 15 | Beute & Kort, 2018 | ○ | ○ | ○ | ○ | ○ | ● | ● | ○ | ○ | 2 | -PCFI = time of day. Bias in analysis mitigated by evaluating fixed, random and covariance of effects. |
| 8 | 16 | Bickham et al., 2015 | ● | ○ | ○ | ○ | ○ | ○ | ○ | ○ | ○ | 1 | -PCFI = EMA vs time diary and survey recall. Bias in analysis addressed with follow up at one year. |
| 9 | 18 | Bos et al., 2019 | ● | ○ | ● | ○ | ○ | ● | ○ | ○ | ○ | 3 | -No PCFI. |
| 10 | 21 | Bower et al., 2010 | ● | ○ | ○ | ○ | ○ | ● | ○ | ○ | ○ | 2 | -PCFI = Effect of depression on the relationship between sleep quality and affect. |
| 11 | 23 | Brose et al., 2017 | ● | ○ | ○ | ○ | ● | ○ | ○ | ○ | ○ | 2 | -PCFI = Controlling for gender. |
| 12 | 24 | Brown et al., 2011 | ● | ○ | ○ | ○ | ○ | ● | ● | ○ | ○ | 3 | -PCFI = Controlling for gender. |
| 13 | 25 | Burns et al., 2011 | ● | ○ | ○ | ○ | ○ | ● | ○ | ○ | ○ | 1 | -PCFI = Self-rated and clinician rated assessment of depression. |
| 14 | 27 | Bylsma et al., 2011 | ● | ○ | ○ | ○ | ○ | ● | ○ | ○ | ○ | 2 | -PCFI = Comparison between one and three days of EMAs. |
| 15 | 29 | Cho et al., 2019 | ● | ○ | ○ | ● | ○ | ○ | ○ | ○ | ○ | 2 | -No standardised measures were employed to validate EMA mood logs. PCFI = personalised vs generalised models. Symptom severity assessed by clinician interview. |
| 16 | 30 | Chow et al., 2017 | ● | ○ | ○ | ○ | ○ | ● | ○ | ● | ○ | 3 | -A point in symptom severity was given due to the lack of measurement of symptomatology in the DASS-21.  -The PCFI was time interval, however this was addressed by the use of multiple types of analyses. |
| 17 | 31 | Chue et al., 2017 | ● | ● | ○ | ○ | ● | ● | ○ | ○ | ○ | 4 | -PCFI = Variation of symptoms over time (follow up 4 months). |
| 18 | 33 | Clasen et al., 2015 | ○ | ○ | ○ | ○ | ○ | ● | ○ | ○ | ○ | 1 | -PCFI = The effect of baseline depression on ruminative style. |
| 19 | 35 | Colombo et al., 2020 | ● | ○ | ○ | ○ | ○ | ● | ● | ○ | ○ | 3 | -PCFI = Validation of PA/NA via EMA against self-reported PA/NA. |
| 20 | 37 | Cormack et al., 2019 | ● | ○ | ○ | ○ | ○ | ● | ● | ○ | ○ | 3 | -Participant poor compliance of task completion excluded from study (however, addressed in study). PCFI = Effect of mood and cognitive ability. |
| 21 | 40 | Cushing et al., 2018 | ○ | ○ | ○ | ● | ○ | ● | ● | ● | ○ | 4 | -PCFI = Pre-post-physical activity. Depression severity assessed via mood questionnaire. |
| 22 | 45 | Dejonckheere | ○ | ○ | ● | ○ | ○ | ● | ○ | ○ | ○ | 2 | -No PCFI |
| 23 | 46 | Demiralp et al., 2012 | ● | ○ | ○ | ○ | ○ | ● | ○ | ○ | ○ | 2 | -PCFI = Effect of emotion intensity and variability on depression severity. |
| 24 | 47 | Depp et al., 2015 | ○ | ○ | ● | ○ | ○ | ● | ○ | ○ | ○ | 2 | -No PCFI |
| 25 | 49 | Di Matteo et al., 2020 | ● | ○ | ● | ○ | ○ | ● | ○ | ○ | ○ | 3 | -No PCFI. |
| 26 | 50 | Di Matteo et al., 2021 | ● | ○ | ● | ○ | ○ | ● | ○ | ○ | ○ | 3 | -No PCFI. |
| 27 | 51 | Dietvorst et al., 2021 | ● | ● | ● | ○ | ○ | ● | ○ | ○ | ○ | 4 | -No PCFI. |
| 28 | 52 | Difrancesco et al., 2018 | ● | ○ | ○ | ○ | ○ | ○ | ○ | ○ | ○ | 1 | -PCFI = Age, sex and education level. |
| 29 | 53 | Eddington et al., 2017 | ○ | ○ | ○ | ○ | ○ | ● | ○ | ○ | ○ | 1 | -PCFI = Effect of time on depressive symptoms in stressful and positive situations. |
| 30 | 56 | Elovainio et al., 2020 | ● | ○ | ○ | ● | ● | ● | ○ | ● | ○ | 5 | -PCFI = Temporal features. |
| 31 | 58 | Fang et al., 2019 | ● | ● | ○ | ○ | ○ | ● | ○ | ○ | ○ | 3 | -PCFI = Controlling for the effect of depression and trait rumination. |
| 32 | 59 | Feiler et al., 2015 | ● | ○ | ○ | ● | ○ | ● | ○ | ● | ○ | 4 | -PCFI = Effects of anxiety. |
| 33 | 65 | Gansner et al., 2020 | ● | ○ | ○ | ○ | ○ | ● | ● | ○ | ○ | 3 | -PCFI = Using app to modify relationship between mental health and problematic internet use. |
| 34 | 66 | Geyer et al., 2018 | ● | ○ | ○ | ○ | ○ | ● | ○ | ○ | ○ | 5 | -PCFI = Evaluation of variability (over time) in interactions effects between enjoyment of social interaction, ratings of effective social interaction and depression. |
| 35 | 67 | Giesbrecht et al., 2012 | ● | ○ | ○ | ○ | ○ | ● | ○ | ○ | ○ | 2 | -PCFI = Gestational age. |
| 36 | 69 | Goldschmidt et al., 2014 | ○ | ○ | ○ | ○ | ○ | ● | ○ | ○ | ○ | 2 | -PCFI = Mediation effect of emotional eating and binge eating. |
| 37 | 71 | Graham-Engeland et al., 2016 | ● | ○ | ○ | ○ | ○ | ● | ● | ○ | ○ | 3 | -PCFI = Age and gender as covariates. Bias in analysis mitigated through between and within person analyses. |
| 38 | 72 | Gruber et al., 2013 | ● | ○ | ● | ○ | ○ | ● | ○ | ○ | ○ | 3 | -No PCFI. |
| 39 | 73 | Hahn et al., 2021 | ● | ○ | ○ | ○ | ○ | ● | ● | ○ | ○ | 3 | -PCFI = Effects of stress |
| 40 | 74 | Hallensleben et al., 2017 | ● | ○ | ● | ○ | ○ | ● | ○ | ○ | ● | 4 | -No PCFI. Bias in analysis due to analysing scores over time with MSSD only. |
| 41 | 76 | Hamilton et al., 2020 | ● | ○ | ○ | ○ | ○ | ● | ● | ○ | ○ | 3 | -PCFI = Age, sex, current depressive symptoms, and school-break timing as covariates. |
| 42 | 77 | Hartmann et al., 2015 | ○ | ○ | ○ | ○ | ○ | ● | ○ | ○ | ○ | 2 | -PCFI = Effect of time. |
| 43 | 79 | Heninga et al., 2019 | ● | ○ | ○ | ○ | ○ | ● | ○ | ○ | ○ | 2 | -PCFI = ‘Trimmed model’ adjusting for familywise error. |
| 44 | 80 | Hepp et al., 2019 | ● | ○ | ○ | ○ | ○ | ● | ○ | ○ | ○ | 2 | -PCFI = Day and person effects on momentary assessments. |
| 45 | 82 | Hershenberg et al., 2017 | ● | ○ | ○ | ○ | ○ | ● | ● | ○ | ○ | 3 | -PCFI = Level of event pleasantness. |
| 46 | 83 | Holmes et al., 2016 | ● | ○ | ○ | ○ | ○ | ● | ○ | ○ | ○ | 3 | -PCFI = Length of baseline assessment of depression. |
| 47 | 85 | Huckins et al., 2020 | ● | ○ | ○ | ○ | ● | ● | ○ | ○ | ○ | 3 | -PCFI = Linear and quadratic random effects. |
| 48 | 86 | Huffziger et al., 2013(a) | ● | ○ | ○ | ○ | ○ | ● | ○ | ○ | ○ | 2 | -PCFI = Time lagged cortisol levels. |
| 49 | 87 | Huffziger et al., 2013(b) | ● | ○ | ○ | ○ | ○ | ● | ○ | ○ | ○ | 2 | -PCFI = Proximal and distant effect with respect to time. |
| 50 | 88 | Hung et al., 2016 | ● | ○ | ● | ○ | ○ | ● | ○ | ○ | ○ | 3 | - No PCFI |
| 51 | 89 | Husky et al., 2009 | ● | ○ | ○ | ○ | ○ | ● | ○ | ○ | ○ | 2 | -PCFI = Cognitive vulnerability for depression and substance use. |
| 52 | 91 | Jacobson et al., 2019 (a) | ● | ○ | ○ | ○ | ○ | ● | ● | ○ | ○ | 3 | -PCFI = Temporal oscillation in symptom severity. |
| 53 | 92 | Jacobson et al., 2019 (b) | ● | ○ | ○ | ○ | ○ | ● | ● | ○ | ○ | 3 | -PCFI = Age and sex. |
| 54 | 93 | Jacobson et al., 2020 (a) | ● | ○ | ○ | ○ | ○ | ● | ○ | ○ | ○ | 2 | -PCFI = Validation of social anxiety digital biomarkers and administered validated questionnaire. |
| 55 | 94 | Jacobson et al., 2020 (b) | ● | ○ | ○ | ○ | ○ | ● | ● | ○ | ○ | 3 | -PCFI = Nomothetic and idiographic variability. |
| 56 | 97 | Jean et al., 2013 | ● | ○ | ● | ○ | ○ | ● | ○ | ○ | ● | 4 | - No PCFI. Only used linear regression. |
| 57 | 100 | Kaufmann et al., 2016 | ● | ○ | ○ | ○ | ○ | ● | ● | ○ | ○ | 3 | -PCFI = Variability of sleep affected by sex and race. |
| 58 | 102 | Khazanov et al., 2019 | ○ | ○ | ○ | ○ | ○ | ● | ○ | ○ | ○ | 1 | -PCFI = Age and sex as covariates. |
| 59 | 103 | Kim et al., 2013 | ● | ● | ○ | ○ | ○ | ● | ○ | ○ | ○ | 3 | -Recruitment process and eligibility criteria not outlined. PCFI = a combination of several fixed and random effects. |
| 60 | 104 | Kim et al., 2014 | ● | ○ | ○ | ○ | ○ | ● | ● | ○ | ○ | 3 | -PCFI = Fixed and random effects. |
| 61 | 105 | Kim et al., 2019 | ● | ○ | ● | ○ | ○ | ● | ○ | ○ | ○ | 3 | -No PCFI. |
| 62 | 106 | Kircanski et al., 2015 | ● | ○ | ● | ○ | ○ | ● | ○ | ○ | ○ | 3 | -No PCFI. |
| 63 | 109 | Koval et al., 2013 | ● | ○ | ● | ○ | ● | ● | ○ | ○ | ○ | 4 | -No PCFI. |
| 64 | 110 | Kramer et al., 2014 | ○ | ○ | ○ | ○ | ○ | ● | ○ | ○ | ○ | 1 | -PCFI = Experimental and pseudo experimental groups to partial out treatment effect. |
| 65 | 114 | Lavender et al., 2013 | ● | ○ | ○ | ○ | ○ | ● | ○ | ○ | ○ | 2 | -PCFI = Controlling for depression. |
| 66 | 121 | Maher et al., 2018 | ● | ○ | ○ | ○ | ○ | ● | ○ | ○ | ○ | 2 | -PCFI = Variability of positive affect. |
| 67 | 122 | Mak & Schneider, 2020 | ● | ○ | ○ | ○ | ○ | ● | ● | ○ | ○ | 3 | -PCFI = Time lagged effects, mean and variability in individual differences, and autoregressive correlations. |
| 68 | 125 | Mata et al., 2012 | ● | ○ | ○ | ○ | ○ | ● | ○ | ○ | ○ | 2 | -PCFI = Effect of length of physical activity on affect. |
| 69 | 126 | McIntyre et al., 2021 | ○ | ○ | ● | ○ | ○ | ● | ○ | ○ | ● | 3 | -No PCFI. Only predictive models were employed. |
| 70 | 128 | Melcher et al., 2021 | ○ | ○ | ● | ○ | ○ | ● | ○ | ○ | ○ | 3 | -No PCFI due to using correlations. |
| 71 | 130 | Minaeva et al., 2020a | ○ | ○ | ○ | ○ | ○ | ● | ○ | ○ | ○ | 1 | -PCFI = Age, gender, and education. |
| 72 | 131 | Minaeva et al., 2020b | ● | ○ | ○ | ○ | ○ | ● | ○ | ○ | ○ | 2 | -PCFI = Age, gender, marital status, socioeconomic status, smoking, BMI, current medication use, and depression severity. |
| 73 | 134 | Moreno et al., 2012 | ○ | ○ | ● | ○ | ○ | ● | ● | ○ | ○ | 3 | -No PCFI. |
| 74 | 135 | Moshe et al., 2021 | ● | ○ | ○ | ○ | ○ | ● | ○ | ○ | ○ | 2 | -PCFI = Hierarchical models controlling for the effects of predictors. |
| 75 | 136 | Moukaddam et al., 2019 | ● | ○ | ○ | ○ | ○ | ● | ○ | ○ | ○ | 2 | -PCFI = Controlling for depression severity to observe bivariate correlations between variables. |
| 76 | 138 | Narziev et al., 2020 | ○ | ○ | ○ | ○ | ● | ● | ○ | ○ | ● | 4 | -No PCFI. |
| 77 | 139 | Nelson et al., 2020 | ● | ○ | ○ | ○ | ○ | ● | ● | ○ | ○ | 3 | -PCFI = Multilevel in modelling. |
| 78 | 141 | Nook et al., 2021 | ○ | ○ | ○ | ○ | ● | ● | ○ | ○ | ○ | 2 | -PCFI = Monthly and moment-level measurements of depression and observing bivariate correlations across variables. |
| 79 | 142 | Nylocks et al., 2019 | ○ | ○ | ○ | ○ | ● | ● | ○ | ○ | ● | 3 | -No PCFI. No subsequent analyses. |
| 80 | 143 | Odgers & Russell 2017 | ● | ○ | ○ | ○ | ○ | ● | ● | ○ | ○ | 3 | -PCFI = Observing effects on group and person level at same day and next day. |
| 81 | 144 | O’Leary et al., 2017 | ○ | ○ | ○ | ○ | ● | ● | ○ | ○ | ○ | 2 | -No PCFI. |
| 82 | 147 | Panaite et al., 2018 | ○ | ○ | ○ | ○ | ○ | ● | ● | ○ | ○ | 3 | -PCFI = Dysphoric affect at baseline as covariate. |
| 83 | 148 | Panaite et al., 2019 | ○ | ○ | ○ | ○ | ○ | ● | ○ | ○ | ○ | 2 | -PCFI = Effect of depressive symptoms on reactivity to event appraisal. |
| 84 | 150 | Pasyugina et al., 2015 | ● | ○ | ○ | ○ | ○ | ● | ○ | ○ | ○ | 2 | -PCFI = Effect of rumination style on depression and affect. |
| 85 | 151 | Pe et al., 2014 | ● | ○ | ○ | ○ | ○ | ● | ○ | ○ | ○ | 2 | -PCFI = Role of gender and variability of emotions. |
| 86 | 152 | Pedrelli et al., 2020 | ● | ○ | ● | ○ | ○ | ● | ○ | ○ | ○ | 3 | -No PCFI. |
| 87 | 153 | Peterson et al., 2020 | ○ | ○ | ● | ○ | ○ | ● | ○ | ○ | ○ | 2 | -No PCFI. |
| 88 | 154 | Place et al., 2017 | ● | ○ | ● | ○ | ○ | ● | ○ | ○ | ○ | 3 | -No PCFI. |
| 89 | 155 | Putnam & McSweeney, 2007 | ● | ○ | ○ | ○ | ○ | ● | ● | ○ | ○ | 3 | -PCFI = EEG. |
| 90 | 157 | Robbins et al., 2011 | ● | ○ | ● | ○ | ○ | ● | ○ | ○ | ○ | 3 | -No PCFI. |
| 91 | 158 | Rodriguez et al., 2021 | ○ | ○ | ○ | ● | ○ | ● | ○ | ○ | ● | 3 | -PCFI = Active and passive social media use. |
| 92 | 183 | Roekel et al., 2016 | ● | ○ | ○ | ○ | ○ | ● | ○ | ○ | ○ | 2 | -PCFI = Age and gender. |
| 93 | 160 | Sagar et al., 2016 | ● | ○ | ○ | ○ | ○ | ● | ● | ○ | ○ | 3 | -PCFI = Marihuana use. |
| 94 | 162 | Schultebraucks et al., 2020 | ● | ○ | ○ | ○ | ○ | N.A. | N.A. | ○ | ○ | 2 | -PCFI = Age. |
| 95 | 163 | Sears et al., 2018 | ○ | ○ | ○ | ○ | ○ | ● | ○ | ○ | ○ | 1 | -PCFI = Time of day. |
| 96 | 166 | Sheets and Armey, 2020 | ● | ○ | ○ | ○ | ○ | ● | ○ | ○ | ○ | 2 | -PCFI = Multilevel modelling. |
| 97 | 167 | Snippe et al., 2016 | ● | ○ | ○ | ○ | ○ | ● | ○ | ○ | ○ | 2 | -PCFI = Personalised feedback. |
| 98 | 168 | Sperry et al., 2018 | ● | ○ | ● | ○ | ○ | ● | ○ | ○ | ○ | 3 | -No PCFI. |
| 99 | 170 | Stasak et al., 2019 | ● | ○ | ○ | ○ | ○ | N.A. | N.A. | ○ | ○ | 1 | -PCFI = Multiple linguistic features. |
| 100 | 171 | Steenkamp et al., 2019 | ● | ○ | ○ | ● | ○ | ● | ○ | ● | ○ | 4 | -PCFI = mediating role of depression on the loneliness-abuse symptoms relationship. |
| 101 | 172 | Thompson et al., 2015 | ● | ○ | ○ | ○ | ○ | ● | ○ | ○ | ○ | 2 | -PCFI = Baseline reaction time as covariate. |
| 102 | 173 | Thompson et al., 2016 | ● | ○ | ○ | ○ | ○ | ● | ○ | ○ | ○ | 2 | -PCFI = Multilevel including person mean and grand mean centered. |
| 103 | 174 | Thompson et al., 2017 | ● | ○ | ○ | ○ | ○ | ● | ● | ○ | ○ | 3 | -PCFI = Short and long term forecasted affect. |
| 104 | 181 | Trull et al., 2008 | ● | ○ | ○ | ○ | ○ | ● | ○ | ○ | ○ | 2 | -PCFI = Time variants and detrended changes. |
| 105 | 184 | Vansteelandt et al., 2019 | ● | ○ | ○ | ○ | ○ | ● | ○ | ● | ○ | 3 | -PCFI = Analysis evaluating observed variance between and within participants. Depression symptom severity only assessed via self-report. |
| 106 | 186 | Verkuil et al., 2015 | ● | ○ | ○ | ○ | ○ | ● | ○ | ○ | ○ | 2 | -PCFI = Time of day, smoking and physical activity. |
| 107 | 187 | Vesel et al., 2020 | ○ | ● | ○ | ○ | ○ | ○ | ● | ○ | ○ | 2 | -PCFI = Multilevel modelling |
| 108 | 188 | Vranceanu et al., 2009 | ● | ○ | ○ | ○ | ○ | ● | ○ | ○ | ○ | 2 | -PCFI = Effect of social conflict. |
| 109 | 189 | Wahle et al., 2016 | ○ | ○ | ○ | ○ | ● | ● | ○ | ○ | ○ | 2 | -PCFI = Treatment via app. |
| 110 | 190 | Wang et al., 2021 | ● | ● | ○ | ○ | ○ | ● | ● | ○ | ○ | 4 | -PCFI = Age and gender. |
| 111 | 191 | Wenze et al., 2006 | ● | ○ | ○ | ○ | ● | ● | ● | ○ | ○ | 4 | -PCFI = Moderating effect of dysphoria on automatic thoughts and affect. |
| 112 | 192 | Wenze et al., 2009 | ● | ○ | ○ | ○ | ○ | ● | ○ | ○ | ○ | 2 | -PCFI = Moderating effect of dysphoria on affect (anger and depressed mood). |
| 113 | 193 | Wenze et al., 2012 | ● | ○ | ● | ● | ○ | ● | ○ | ● | ○ | 5 | -Measurement of depressive symptoms via mood questionnaire. |
| 114 | 194 | Wenze et al., 2018 | ● | ○ | ○ | ○ | ○ | ● | ● | ○ | ○ | 3 | -PCFI = Multilevel modelling controlling for individual differences and time lagged effects. |
| 115 | 196 | Worten-Chaudhari et al., 2017 | ● | ○ | ● | ○ | ○ | ● | ○ | ○ | ○ | 3 | -No PCFI |
| 116 | 197 | Wu et al., 2016 | ● | ○ | ○ | ○ | ○ | ● | ○ | ○ | ○ | 2 | -PCFI = Time of day. |
| 117 | 198 | Zhang et al., 2019 | ○ | ○ | ● | ○ | ○ | N.A. | N.A. | ○ | ○ | 2 | -No PCFI. |
| 118 | 200 | Zulueta et al., 2018 | ● | ○ | ○ | ○ | ○ | ● | ○ | ○ | ○ | 2 | -PCE = Circadian baseline similarity |
|  |  | Total | 89 | 7 | 28 | 7 | 12 | 111 | 31 | 7 | 6 |  |  |
|  |  |  | Sample randomization / self-selected sampling^1^ | Participant eligibility criteria^2^ | Confounding factor identified^3^ | Measurement bias^4^ | Adequate participant description^5^ | Follow up longer than one year^6^ | Participant attrition^7^ | Symptom severity^8^ | Bias in alaysis^9^ | Total |  |

***Scoring system*** *=* A point was given for each criterion not addressed, with possible scores ranging from 0-9 for longitudinal studies and 0-7 for cross-sectional studies, and higher scores represent higher risk of bias.

^1^*Sample randomization* (sampling bias): ○ randomized sample (0 point), ● researcher selected sample (1 point). This point refers to sample selection, with randomization perceived as a more robust method to select participants and thus minimize the probability of researcher bias.

^2^*Participant eligibility criteria*: ○ yes (0 point), ● no (1 point). Clearly stated participant eligibility criteria enable readers to understand the method employed and thus increases transparency. Articles included in this review failing to provide such information were awarded one point suggesting an increased risk of bias.

^3^*Potential confounding factor identified* (PCFI): ○ yes (adjusted data; 0 point), ● yes (non-adjusted data), ● no (1 point). Identification of confounding factors may mitigate implicit risk of bias. While some studies identified potential confounding factors, minimization of bias was attained only when results were adjusted to reflect confounding effects.

^4^*Measurement bias*: ○ standardised disorder specific measures (0 point), ● non-standardised measures (1 point). The use of widely adopted and standardized measures (such as BDI or CES-D) to capture depression minimizes risk of bias.

^5^*Adequate participant description*: ○ yes (0 point), ● no (1 point). Clear description of participants’ demographic increases transparency. For example, failing to include mean age, or gender distribution in employed samples would award a specific study included in this review with one point.

^6^*Follow up longer than one year*: ○ yes (0 point), ● no (1 point)

^7^*Participant attrition*: ○ explained (0 point), ● not explained (1 point). Including information describing participant attrition (and how it was handled) increases research transparency.

^8^*Appropriate measurement of symptom severity*: ○ yes (0 point), ● no (1 point). Studies were awarded one point when depression severity was only assessed via non-standardized self-rated questionnaires. For example, studies asking participants to rate their levels of depression was not considered an ‘appropriate’ measurement of symptom severity. However, no points were awarded when studies employed standardized measures to capture depression.

^9^*Bias in analysis mitigated conducting several statistical analyses*: ○ yes (0 point), ● no (1 point).
